# Delivery of Pleckstrin‐Homology Domains Suppresses PI3K/Akt Signaling and Breast Cancer Metastasis

**DOI:** 10.1002/advs.202518339

**Published:** 2026-03-30

**Authors:** Matthew Eason, Anindya Sen, Suhan Cho, Se Jong Lee, Keyata Thompson, Md Musavvir Mahmud, Poornima Dubey, Talia Guardia, Anthony Kim, Stuart Martin, Nathan Wright, Konstantinos Konstantopoulos, Aikaterini Kontrogianni‐Konstantopoulos

**Affiliations:** ^1^ Department of Biochemistry and Molecular Biology University of Maryland School of Medicine Baltimore Maryland USA; ^2^ Marlene and Stewart Greenebaum Comprehensive Cancer Center Baltimore Maryland USA; ^3^ Department of Chemical and Biomolecular Engineering The Johns Hopkins University Baltimore Maryland USA; ^4^ Institute For NanoBioTechnology Johns Hopkins University Baltimore Maryland USA; ^5^ Department of Pharmacology and Physiology University of Maryland School of Medicine Baltimore Maryland USA; ^6^ Department of Neurosurgery University of Maryland School of Medicine Baltimore Maryland USA; ^7^ Fischell Department of Bioengineering A. James Clarke School of Engineering University of Maryland College Park Maryland USA; ^8^ Department of Chemistry and Biochemistry James Madison University Harrisonburg Virginia USA; ^9^ Department of Oncology The Sidney Kimmel Comprehensive Cancer Center The Johns Hopkins University School of Medicine Baltimore Maryland USA; ^10^ Department of Biomedical Engineering Johns Hopkins University Baltimore Maryland USA

**Keywords:** breast cancer, metastasis suppressors, obscurin, PI3K/Akt signaling, PI3K inhibitors, pleckstrin homology domains

## Abstract

Current cancer therapies inhibit tumor growth but fail to target metastatic dissemination. Obscurin (720–870 kDa), a giant signaling protein localizing to the breast epithelial cell membrane, is a metastasis suppressor commonly lost in breast cancer. Obscurin loss upregulates the oncogenic PI3K/Akt axis. While restoring obscurin expression is crucial from a translational standpoint, it poses major challenges due to its immense size. Herein, we overcome this hurdle by delivering a mini‐obscurin—comprising the obscurin‐pleckstrin homology (PH) domain, which is ∼50‐times smaller than the full‐length protein—into aggressive breast cancer cells via adenovirus and lipid nanoparticles. Mechanistically, the obscurin‐PH‐domain interacts with the PI3K‐p85 regulatory subunit. Membrane‐targeted obscurin‐PH sequesters p85, suppressing PI3K/Akt activity. p85‐sequestration eliminates filopodia, hampering migration and adhesion to pre‐metastatic niche extracellular matrix substrates. This intervention further eradicates invadopodia and reduces matrix metalloproteinase expression, blocking invasion, dissemination, and metastasis. We recapitulate this phenotype using the structurally homologous kalirin and PLCγ1 PH‐domains and ultimately uncover a family of nine PH‐domains that may act as PI3K inhibitors, unified by the “*p*85 *i*nhibitory *m*etastasis *s*uppressor” (PIMS) motif, mediating this effect. This work engineers a first‐in‐class group of non‐chemical PI3K inhibitors, uniquely targeting the PI3K‐p85 subunit, galvanizing novel gene therapies for treating metastatic breast cancer.

## Graphical abstract

Current therapies curb tumor growth but not metastasis. Obscurin, a giant metastasis suppressor lost in breast cancer, restrains PI3K/Akt signaling but is impractical to restore. We deploy a mini‐obscurin, comprising the obscurin‐PH‐domain, which sequesters PI3K‐p85, potently suppressing invasion and metastasis. Strikingly, we identify nine PH‐domains unified by the presence of the “p85 inhibitory metastasis suppressor” (PIMS) motif, establishing a first‐in‐class family of non‐chemical PI3K inhibitors and a new framework for metastatic breast cancer gene therapy.

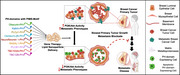

## 1 Introduction

Obscurin (720–870 kDa) is a giant cytoskeletal protein encoded by the single *OBSCN* gene [1, 2]. *OBSCN* loss has been implicated in breast cancer formation and progression [1], as well as in chemotherapy and radiotherapy resistance [3, 4, 5, 6, 7, 8, 9]. Accordingly, analysis of Kaplan‐Meier datasets indicates that low *OBSCN* levels correlate with significantly reduced survival and relapse‐free survival in breast cancer patients [4]. Conversely, higher *OBSCN* levels correlate with increased patient responsiveness to anthracyclines, commonly used in metastatic breast cancer treatment [4]. Consistent with these observations, obscurin is abundantly expressed in normal breast epithelium, where it preferentially concentrates at the cell membrane and perinuclearly at the Golgi, but is dramatically diminished in advanced stage breast cancer biopsies and cell lines [3, 4, 5, 6, 8]. Knockdown of obscurin in normal breast epithelium induces epithelial to mesenchymal transition (EMT) and stemness [8], reduces cell sensitivity to common chemotherapies (e.g., paclitaxel) [6], enhances migration and invasion, and promotes metastasis [8]. Mechanistically, loss of obscurin results in upregulation of the PI3K/Akt axis [5], which is altered in 30%–40% of invasive breast carcinomas [10, 11], driving breast cancer cell growth and metastatic dissemination via a two‐tiered system of gene expression and cytoskeletal changes [10, 12, 13, 14, 15, 16]. Obscurin forms a complex with the PI3K‐p85 regulatory subunit [5]. Their interaction is direct and mediated by the obscurin‐Pleckstrin Homology (PH) domain and the PI3K‐p85‐Src Homology 3 (SH3) domain with a K_D_ of ∼50 nM [5], suggesting that obscurin may be an upstream regulator of the PI3K/Akt axis in normal breast epithelium.

While restoration of obscurin expression in triple negative breast cancer (TNBC) cells via CRISPR‐activation suppresses cell migration, invasion, and dissemination in vitro and metastasis in vivo [4], the translational potential of this approach is limited due to obscurin's gigantic size. To overcome this challenge, we interrogated the suppressive properties of a mini‐obscurin, consisting of the obscurin‐PH domain—which is ∼50 times smaller than the full‐length protein—in two of the most aggressive breast cancer subtypes, i.e., triple negative and HER2+. We demonstrate that obscurin‐PH is a potent PI3K inhibitor with anti‐growth and anti‐metastatic capabilities and further uncover a new class of PI3K inhibitors, in the form of a subgroup of nine structurally homologous PH‐domains, uniquely targeting the p85 regulatory subunit.

Importantly, our approach is distinct from prior and ongoing strategies that focus on the development of small molecule PI3K inhibitors exclusively targeting the p110*α* catalytic subunit, which however exhibit dose‐limiting toxicity and are FDA approved solely for metastatic estrogen receptor‐positive breast cancer that has failed systemic hormone therapy [10]. In sum, our work illustrates the therapeutic potential of a new class of non‐chemical PI3K inhibitors in the form of the PH‐domain, which can be used as nanoparticle‐delivered local gene therapy for the treatment of metastatic breast cancer beyond the hormone receptor‐positive subtype.

## 2 Results

### 2.1 The Obscurin‐PH Domain Suppresses PI3K/Akt Signaling and Breast Cancer Cell Migration

**FIGURE 1:**
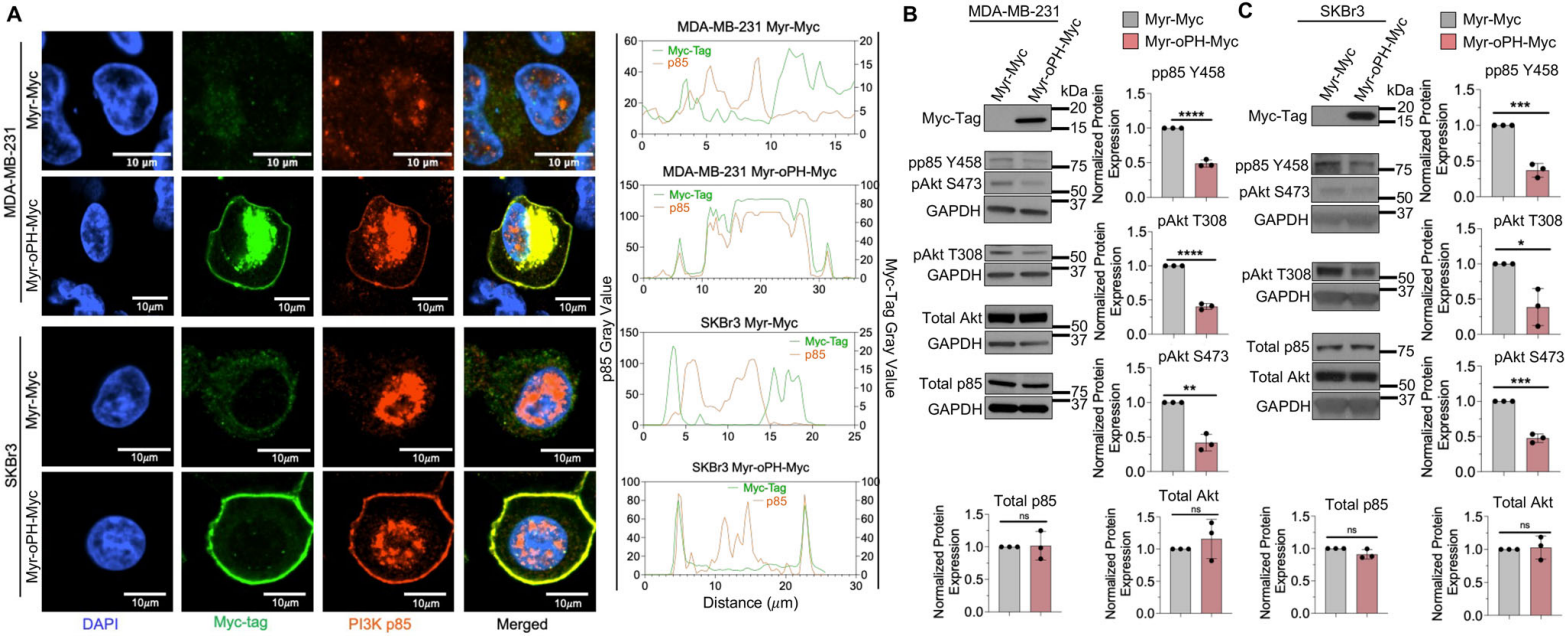
Sequestration of PI3K‐p85 by the obscurin PH‐domain suppresses PI3K/Akt activation. (A) Representative confocal images of adenovirus‐transduced MDA‐MB‐231 and SKBr3 cells expressing the Myr‐Myc and Myr‐oPH‐Myc constructs; DAPI (blue), Myc‐Tag (green), PI3K‐p85 (orange), and merged Myc‐tag/PI3K‐p85 (yellow). Cells of each treatment group vary significantly in size; scale bars differ to visualize the entire cell. Line composite graphs display PI3K‐p85 and Myc‐tag gray values (pixel intensity). (B,C) Immunoblots of MDA‐MB‐231 (B) and SKBr3 (C) cells expressing the Myr‐Myc and Myr‐oPH‐Myc constructs probed for PI3K‐p85 Y458 and pAkt T308/S473 phosphorylation levels. Densitometry values are presented as expression relative to the Myr‐Myc control, set at “1” (*n* = 3 independent experiments); two‐tailed t‐test. All data are plotted as mean ± SD; ^*^ *p*<0.05; ^**^ *p*<0.01; ^***^ *p*<0.001; ^****^ *p*<0.0001.

The obscurin‐PH domain binds directly to the PI3K‐p85 regulatory subunit, and small molecule inhibitors targeting the PI3K/Akt axis in obscurin‐deficient breast epithelial cells block cell migration and invasion [5]. We therefore set forth to explore the suppressive potential of the obscurin‐PH domain on PI3K/Akt activity and downstream cellular processes that mediate metastatic spread. To mobilize the obscurin‐PH domain to the cell membrane, we added a myristoylation tag [17] at its N‐terminus along with a Myc‐tag at its C‐terminus for ease of detection (Figure S1A). We then asked if ectopic expression of myristoylated obscurin‐PH domain in highly aggressive triple negative/claudin‐low MDA‐MB‐231 and HER2+ SKBr3 breast cancer cell lines, which exhibit ∼50% reduction in obscurin levels compared to non‐tumorigenic MCF10A breast epithelial cells (Figure S1B), restores the anti‐metastatic function of full‐length obscurin. Adenovirally‐mediated expression of myristoylated obscurin‐PH (Myr‐oPH‐Myc) in either cell line confirmed its cell membrane/perinuclear localization (Figure 1A) and robust expression (Figure 1B,C). Although detection of control Myr‐Myc protein (<1 kDa) was not feasible via immunoblotting, a diffuse faint distribution was observed via immunofluorescence (Figure 1A). Ectopic expression of Myr‐oPH‐Myc, but not control Myr‐Myc protein, led to re‐distribution of the PI3K‐p85 subunit from a nuclear/cytoplasmic punctate localization to a cell membrane/perinuclear accumulation, coincident with Myr‐oPH‐Myc (Figure 1A). The Myr‐oPH‐Myc mediated translocation of the PI3K‐p85 subunit was accompanied by significant suppression of PI3K/Akt activity, as evidenced by the reduced phosphorylation levels of activating p85‐Tyr458 as well as Akt‐Thr308 and Akt‐Ser473 (Figure 1B,C), whereas total p85 and Akt levels remained unchanged (Figure 1B,C).

Given that Myr‐oPH‐Myc sequesters p85 to the cell membrane and perinuclear space, reducing p85 activation and suppressing PI3K/Akt signaling, and active PI3K consists of a p85/p110α heterodimer [18], we next investigated if the obscurin‐PH domain affects the p85/p110α heterodimer complex. Ectopic expression of Myr‐oPH‐Myc, but not control Myr‐Myc, in MDA‐MB‐231 cells led to sequestration of the PI3K‐p110α subunit—similar to the PI3K‐p85 subunit—from a diffuse perinuclear distribution to a hybrid cell membrane/perinuclear accumulation coincident with Myr‐oPH‐Myc (Figure S1C). Consistent with the presence of a complex comprising the PI3K‐p85/p110α heterodimer and obscurin‐PH, immunoprecipitate fractions from MDA‐MB‐231 cells expressing Myr‐oPH‐Myc, but not Myr‐Myc, generated with antibodies to p85 or p110α confirmed the association of p85, p110α, and Myr‐oPH‐Myc (Figure S1D). Together, these results reveal that ectopic expression of obscurin‐PH sequesters p85 to the cell membrane/perinuclear space in complex with p110α.

**FIGURE 2:**
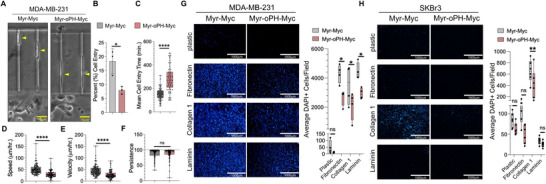
The obscurin PH‐domain reduces breast cancer cell migration and adhesion. (A) Representative images of MDA‐MB‐231 single cells (yellow arrowheads), expressing the Myr‐Myc or Myr‐oPH‐Myc construct, migrating through microchannels. (B) Percent (%) cell entry into microchannels, mean cell entry time (min; C), single cell speed (µm/h; D), velocity (µm/h; E), and persistence (F) of MDA‐MB‐231 cells expressing the Myr‐Myc or Myr‐oPH‐Myc constructs (*n* = 88–92 single cells per construct pooled from three independent experiments); two‐tailed t‐test (B and C) and Mann‐Whitney test (D–F). (G,H) Representative wide‐field fluorescent images of Myr‐Myc or Myr‐oPH‐Myc expressing MDA‐MB‐231 (G) and SKBr3 (H) cells labelled with DAPI (blue), following adhesion to plastic, fibronectin, collagen I, or laminin. Average DAPI‐stained cells/well are plotted (*n* = 4–6 independent experiments per condition); two‐way ANOVA with Sidak's multiple comparison test. All data are plotted as mean ± SD; ^*^ *p*<0.05; ^**^ *p*<0.01; ^****^ *p*<0.0001.

Consistent with the critical role of the PI3K/Akt axis in cancer cell migration [5, 14, 15, 19], ectopic expression of Myr‐oPH‐Myc in MDA‐MB‐231 and SKBr3 cells markedly reduced collective and chemotactic cell migration, as assessed by wound healing (Figure S2A, B) and Transwell (Figure S2C, D) migration assays, respectively. Single cell migration was similarly affected, as assessed in confining microchannels of 10 µm height x 3 µm width (Figure 2A; Video S1), where MDA‐MB‐231 cells transduced with Myr‐oPH‐Myc exhibited significantly decreased capability to enter the microchannels (Figure 2B) and required significantly longer time to do so (Figure 2C), compared to cells expressing control Myr‐Myc. Moreover, Myr‐oPH‐Myc transduced MDA‐MB‐231 cells displayed considerably reduced speed (Figure 2D) and velocity (Figure 2E), although persistence (net displacement/total distance traveled) was unaltered (Figure 2F).

### 2.2 The Obscurin‐PH Domain Decreases Breast Cancer Cell Adhesion and Ablates Filopodia Formation

Cancer cells shedding from primary breast tumors metastasize and adhere to secondary organ sites by utilizing pre‐metastatic niche‐specific extracellular matrix (ECM) proteins [20]. Prior work has shown that collagen I mediates colonization of the bone and peritoneal pre‐metastatic niches, fibronectin the pulmonary and liver pre‐metastatic niches, and laminin the brain pre‐metastatic niche [21]. To interrogate if restoration of obscurin‐PH expression impedes adhesion to pre‐metastatic niche ECM proteins, we performed adhesion assays on plastic, collagen I, fibronectin, and laminin. MDA‐MB‐231 cells expressing control Myr‐Myc protein ably adhered on all three pre‐metastatic ECM substrates (Figure 2G), consistent with the highly aggressive nature of their triple negative/claudin‐low molecular subtype that promiscuously metastasizes to multiple organ sites [22]. Contrary to MDA‐MB‐231, SKBr3 cells transduced with control Myr‐Myc protein adhered effectively to collagen I, but not fibronectin or laminin, in agreement with their HER2+ molecular phenotype that robustly colonizes the peritoneum [23] (Figure 2H). Interestingly, MDA‐MB‐231 cells expressing Myr‐oPH‐Myc exhibited indiscriminately reduced adhesion on all three pre‐metastatic niche ECM substrates (Figure 2G), and SKBr3 cells expressing Myr‐oPH‐Myc displayed decreased adhesion to collagen I (Figure 2H).

**FIGURE 3:**
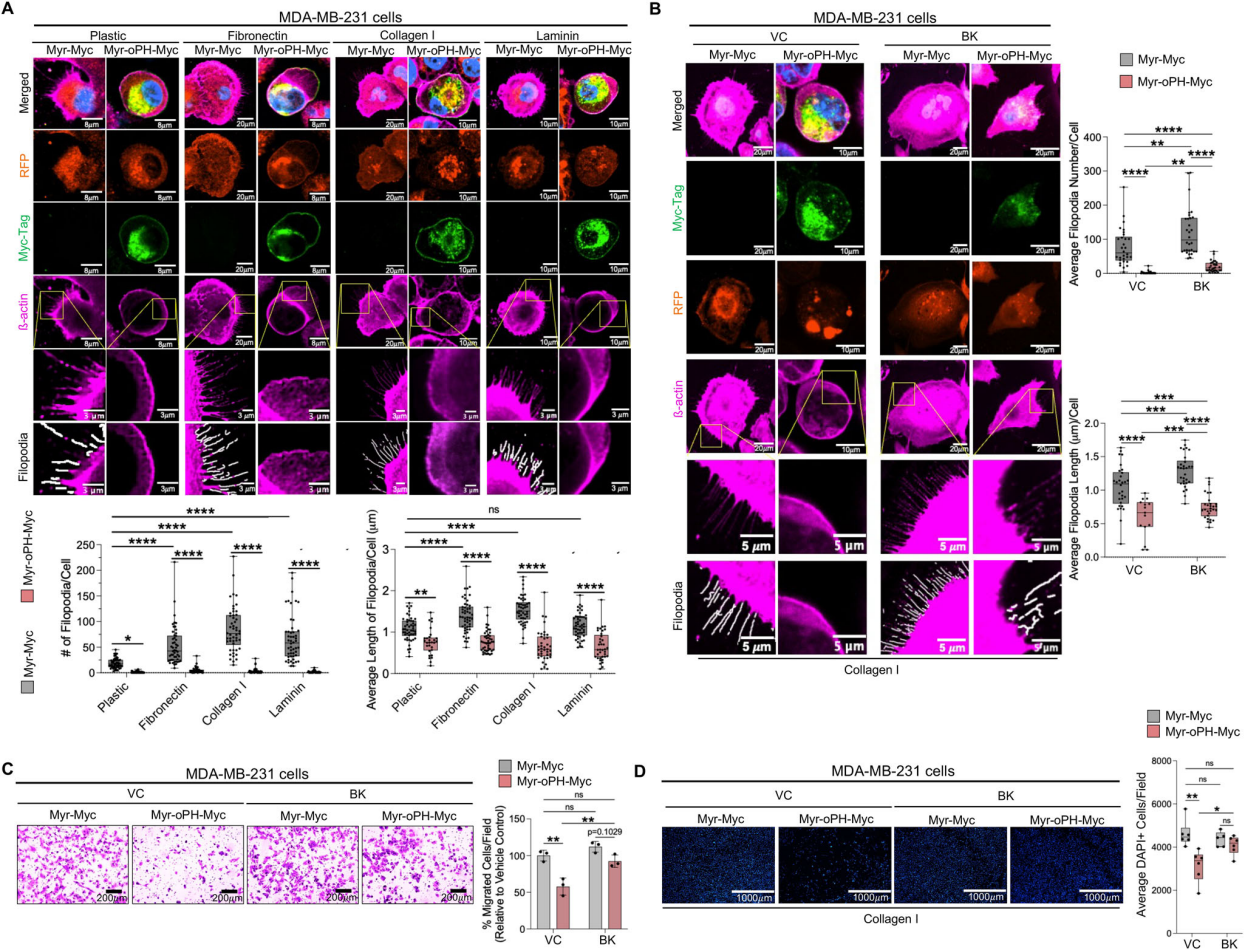
Filopodia ablation by the obscurin PH‐domain inhibits breast cancer cell migration and adhesion. (A) Representative confocal images of adenovirus‐transduced, RFP‐positive MDA‐MB‐231 cells expressing the Myr‐Myc or Myr‐oPH‐Myc construct plated on plastic, fibronectin, collagen I, or laminin substrates; RFP (orange), Myc‐tag (green), β‐actin (magenta), DAPI (blue), and filopodia FiloQuant Overlay mask (white). Cells and filopodia of each treatment group vary significantly in size; scale bars differ to visualize the entire cell and their filopodia. Average number and length (µm) of filopodia (FiloQuant) are plotted per cell, per extracellular matrix substrate (*n* = 50 cells per condition pooled from five independent experiments; 10 cells per experiment). (B) Representative confocal images of adenovirus‐transduced, RFP‐positive MDA‐MB‐231 cells expressing the Myr‐Myc or Myr‐oPH‐Myc construct plated on collagen I, treated with vehicle control (VC) or 100 ng/mL bradykinin (BK); RFP (orange), Myc‐tag (green), β‐actin (magenta), DAPI (blue), and filopodia FiloQuant Overlay mask (white). Cells and filopodia of each treatment group vary significantly in size; scale bars differ to visualize the entire cell and their filopodia. Average number and length (µm) of filopodia (FiloQuant) are plotted per cell, per treatment group (*n* = 30 cells per treatment pooled from three independent experiments; 10 cells per experiment). (C) Representative brightfield images of transwell migration assays of MDA‐MB‐231 cells expressing the Myr‐Myc or Myr‐oPH‐Myc construct, treated with vehicle control (VC) or 100 ng/mL bradykinin (BK). The percent (%) migrated cells per field are plotted, per treatment group (*n* = 3 independent experiments). (D) Representative images of Myr‐Myc and Myr‐oPH‐Myc expressing MDA‐MB‐231 cells stained with DAPI (blue) to allow visualization of nuclei, treated with vehicle control (VC) or 100 ng/mL bradykinin (BK), following adhesion to collagen I. Average DAPI‐labeled cells/well are plotted (*n* = 6 independent experiments). All statistical tests were performed with two‐way ANOVA followed by Tukey's multiple comparison test. All data are plotted as mean ± SD; ^*^ *p*<0.05; ^**^ *p*<0.01; ^***^ *p*<0.001; ^****^ *p*<0.0001.

Actin is a pinnacle driver of cell morphological changes, and activated PI3K/Akt signaling orchestrates cell migration, adhesion, and metastasis through shifts in actin nucleation [24]. This actin remodeling gives rise to filopodia, which are dynamic, actin‐rich structures of ≥1 µm in length protruding from the cell edge [25]. We therefore assessed filopodia formation via actin immunostaining of Myr‐Myc and Myr‐oPH‐Myc expressing MDA‐MB‐231 and SKBr3 cells. Successfully transduced cells were identified via RFP fluorescence (expressed by the bicistronic adenoviral vector) in addition to Myc‐tag immunolabeling. True to their highly aggressive phenotype, MDA‐MB‐231 cells expressing control Myr‐Myc extended filopodia on plastic, a property that was markedly enhanced in the presence of the ECM proteins fibronectin, collagen I, and laminin (Figure 3A), in agreement with earlier reports [26]. Remarkably, Myr‐oPH‐Myc expressing MDA‐MB‐231 cells failed to form filopodia, irrespective of the surrounding ECM environment (Figure 3A). Consistent with the tropic adhesion of SKBr3 cells to collagen I (Figure 2H), cells transduced with control Myr‐Myc formed numerous and long filopodia when plated on collagen I, and only scarce and short protrusions on plastic, fibronectin, and laminin (Figure S3). Remarkably, ectopic expression of Myr‐oPH‐Myc abolished filopodia formation on collagen I entirely (Figure S3).

To investigate a potential direct link between the obscurin‐PH mediated suppression of cell migration and adhesion with filopodia depletion, we treated MDA‐MB‐231 cells plated on collagen I—the most effective filopodia‐inducing ECM protein [26]—with supraphysiological levels of the potent inflammatory mediator bradykinin [25]. Notably, supraphysiological levels of bradykinin have been shown to bypass PI3K/Akt entirely and induce filopodia formation in Swiss 3T3 fibroblasts via activation of the small GTPase Cdc42 [25]. Consistent with this, bradykinin treatment increased both the number and length of filopodia in MDA‐MB‐231 cells transduced with either Myr‐Myc or Myr‐oPH‐Myc, relative to their respective vehicle‐treated cells (Figure 3B). Interestingly though, bradykinin rescue of filopodia in Myr‐oPH‐Myc expressing cells was less effective than in Myr‐Myc cells, indicating that bradykinin can only partially restore filopodia formation in the presence of the obscurin‐PH domain, thereby further underscoring obscurin‐PH's efficacy in filopodia ablation (Figure 3B). In accordance with a direct link between obscurin‐PH‐mediated filopodia abrogation and reduced cell migration and adhesion, bradykinin‐treated MDA‐MB‐231 cells expressing Myr‐oPH‐Myc displayed increased migration (Figure 3C) and adhesion to collagen I (Figure 3D), compared to vehicle‐treated Myr‐oPH‐Myc cells.

### 2.3 The Obscurin‐PH Domain Suppresses Breast Cancer Cell MMP Expression, Invadopodia Formation, and Dissemination

**FIGURE 4:**
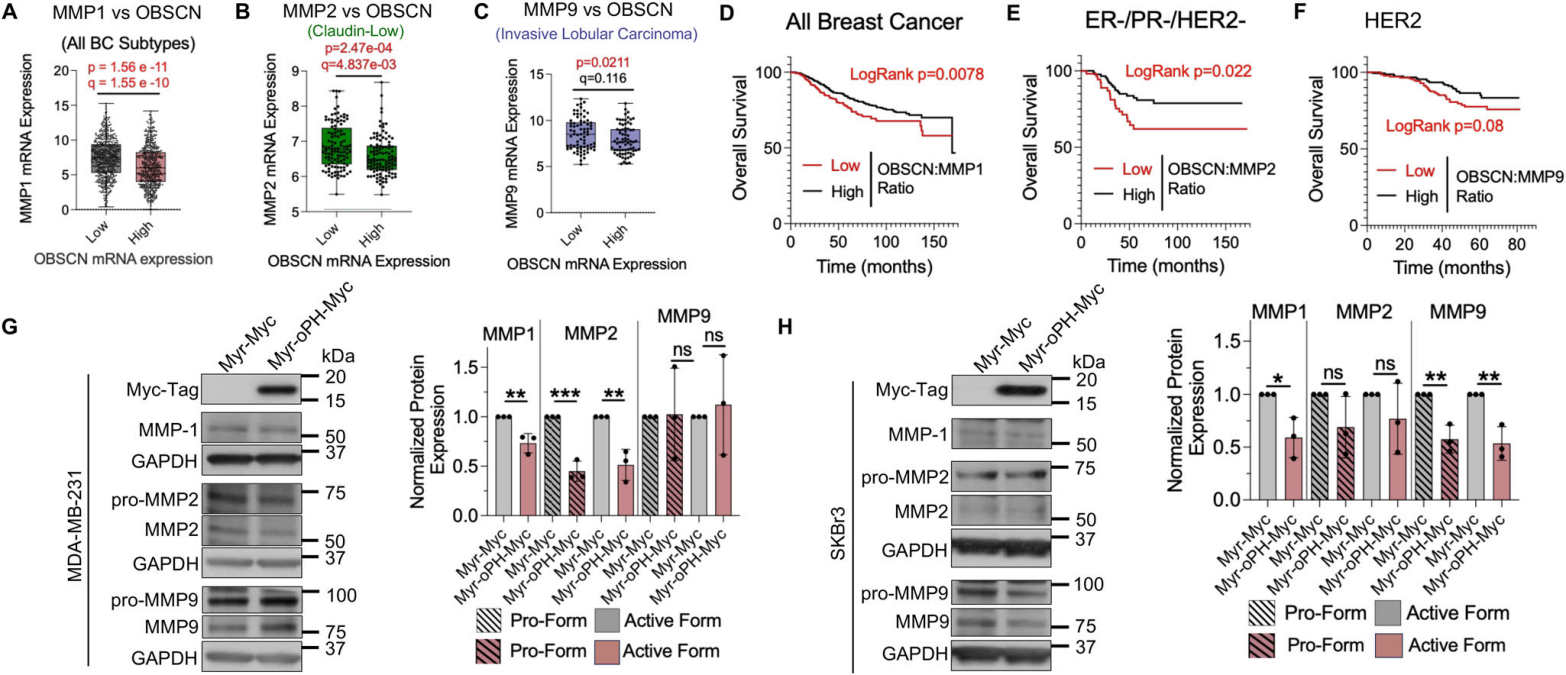
Ectopic expression of the obscurin PH‐domain silences breast cancer matrix metalloproteinase (MMPs) expression. (A‐C) cBioPortal Illumina HT‐12 v3 microarray (Breast Cancer METABRIC, Nature 2012 & Nat Commun 2016) MMP1 expression for all breast carcinoma subtypes (*n* = 541 patient samples per group; A), MMP2 expression for claudin‐low subtype breast cancer (*n* = 109 patient samples per group; B), and MMP9 expression for invasive lobular carcinoma (ILC) subtype breast cancer (*n* = 73 patient samples per group; C), separated by low and high *OBSCN* mRNA expression (median); p‐values by two‐tailed t‐test; q‐values by Benjamini‐Hochberg procedure. (D‐F) Kaplan Meier Plotter analysis (http://kmplot.com/analysis/index.php?p = service) of overall survival via low and high *OBSCN:MMP* expression ratio for (D) all breast cancer subtypes by *OBSCN:MMP1*, (E) ER‐/PR‐/HER2‐ by *OBSCN:MMP2*, and (F) HER2 by *OBSCN:MMP9*; LogRank test. (G‐H) Western blot of Myr‐Myc or Myr‐oPH‐Myc expressing MDA‐MB‐231 (G) and SKBr3 (H) cells for MMP1, MMP2, and MMP9 expression; (*n* = 3 independent experiments), two‐tailed t‐test. Data are plotted as mean ± SD; ^*^ *p*<0.05; ^**^ *p*<0.01; ^***^ *p*<0.001.

To establish metastatic colonies, disseminating tumor cells are required to invade through the dense ECM microenvironment of the tissue parenchyma [27]. Specifically, invading breast cancer cells express and secrete matrix metalloproteinases (MMP) 1, 2, and 9 downstream of upregulated PI3K/Akt signaling to degrade microenvironmental ECM barriers [19, 28]. RNAseq gene expression analysis using the TCGA Wanderer tool (maplab.imppc.org) confirmed markedly increased *MMP1* and *MMP9* expression in invasive breast tumors, independently of their molecular subtype (Figure S4A,B), relative to normal breast tissue, whereas evaluation of the Metabric breast cancer dataset using cBioPortal (cBioportal.org) indicated significantly elevated *MMP2* expression uniquely in claudin‐low breast tumors (Figure S4C). Considering that obscurin loss drives upregulation of the PI3K/Akt axis [5, 8], we investigated if human breast tumors displaying reduced obscurin expression contain increased levels of *MMP 1*, *2*, and/or *9*. Analysis of the 2018 TCGA PanCancer Atlas Invasive Breast Cancer dataset using cBioPortal, including all breast tumor molecular subtypes, revealed a positive correlation between low(er) *OBSCN* and high(er) *MMP1* mRNA levels (Figure 4A). Moreover, use of the Metabric breast cancer dataset showed a positive correlation between low(er) *OBSCN* and high(er) *MMP2* (Figure 4B) and *MMP9* (Figure 4C) mRNA levels in claudin‐low tumors and invasive lobular carcinomas (ILC), respectively. Relatedly, lower *OBSCN:MMP1* ratio correlates with significantly reduced overall survival of breast cancer patients independently of molecular differentiation (kmplot.com; Figure 4D), while lower *OBSCN:MMP2* ratio is linked to markedly decreased overall survival of ER‐/PR‐/HER2‐patients (kmplot.com, comprising the TNBC basal and claudin‐low subtypes; Figure 4E), and lower *OBSCN:MMP9* ratio is associated with a trending decline of overall survival of HER2+ patients (kmplot.com; Figure 4F).

Given these intriguing observations using patient datasets (Figure 4A–F), we investigated if ectopic expression of the obscurin‐PH domain attenuates MMP expression. In agreement with the bioinformatics human data, triple negative/claudin‐low MDA‐MB‐231 cells transduced with Myr‐oPH‐Myc contained significantly decreased levels of MMP1 and MMP2 compared to cells transduced with control Myr‐Myc, whereas MMP9 expression was unaltered (Figure 4G). Conversely, ILC‐like, HER2+ SKBr3 cells transduced with Myr‐oPH‐Myc expressed markedly reduced levels of MMP1 and MMP9, compared to cells transduced with control Myr‐Myc, whereas MMP2 levels were unchanged (Figure 4H). These findings were further confirmed by immunofluorescence staining of Myr‐oPH‐Myc expressing MDA‐MB‐231 and SKBr3 cells cultured on gelatin, which mirrors the 3‐dimensional (3D) microenvironment surrounding breast cancer cells, demonstrating decreased levels of the molecular subtype‐specific MMP2 and MMP9, respectively (Figure S4D,E).

**FIGURE 5:**
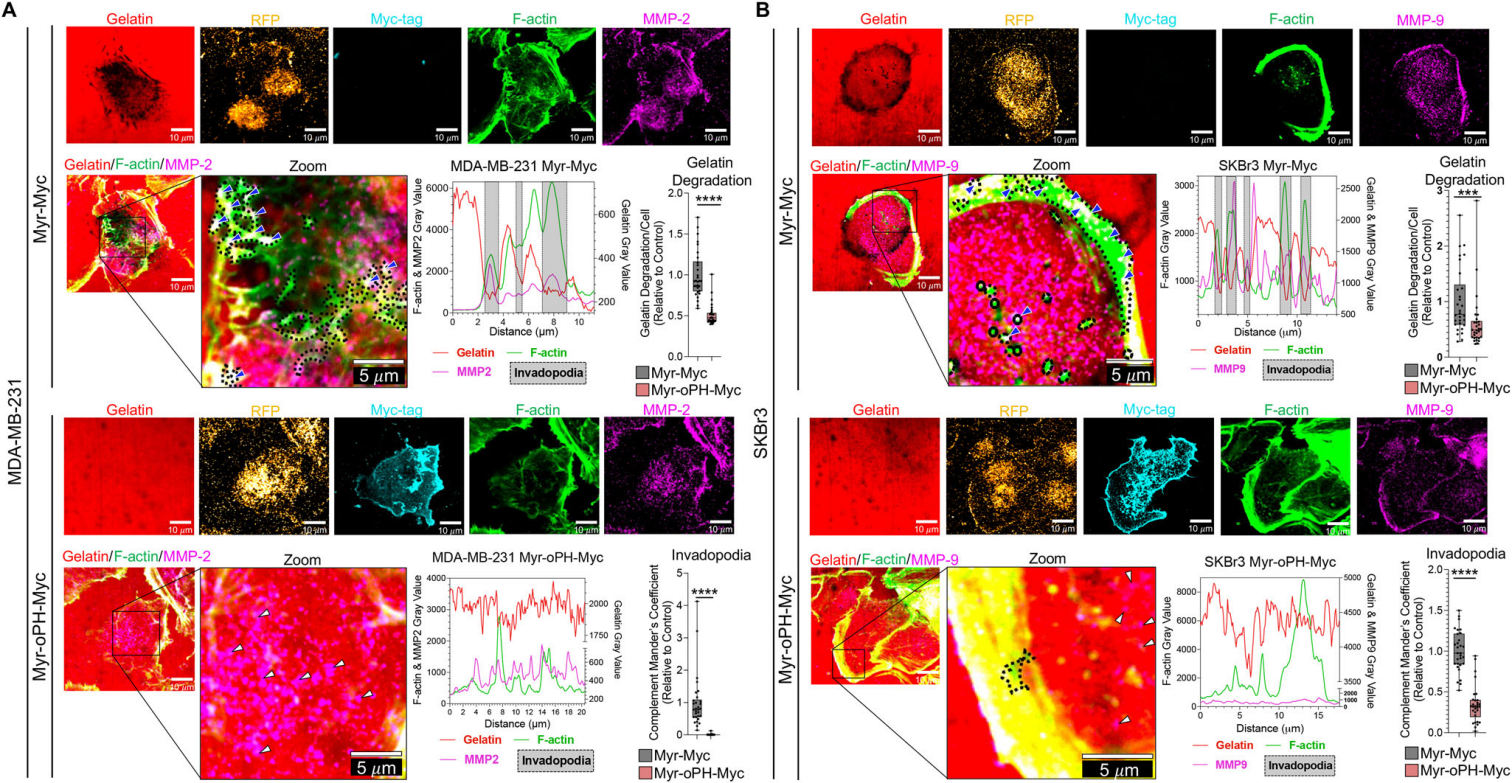
Ectopic expression of the obscurin PH‐domain blocks breast cancer cell invadopodia formation to inhibit invasion. (A,B) Representative confocal images of gelatin invadopodia assays for Myr‐Myc or Myr‐oPH‐Myc transduced MDA‐MB‐231 (A) and SKBr3 (B) cells; Gelatin (red), RFP (gold), Myc‐tag (Cyan), F‐actin (green), and MMP‐2 (MDA‐MB‐231; magenta) and MMP‐9 (SKBr3; magenta). Zoomed images include borders of invadopodia (black dotted lines), MMP‐positive invadopodia (blue arrowheads), MMP‐positive puncta (white arrowheads). Line composite graphs display F‐actin, MMP‐2 (MDA‐MB‐231), MMP‐9 (SKBr3), and Gelatin Mean Gray Values; cell regions with invadopodia are denoted by grey boxes. Gelatin degradation/cell and invadopodia abundance (calculated as the complement Gelatin/F‐actin Mander's coefficient relative to Myr‐Myc) are plotted; (*n* = 30 cells per construct pooled from three independent experiments; 10 cells per experiment); Mann‐Whitney test. Data are plotted as mean ± SD; ^***^ *p*<0.001; ^****^ *p*<0.0001.

In addition to increasing MMP expression, invading breast cancer cells must traffic MMPs from the Golgi to the tips of PI3K‐regulated, actin‐based invadopodia to mediate their secretion and subsequent matrix degradation [29]. To assess the ability of the obscurin‐PH domain to suppress invadopodia formation, we performed gelatin invadopodia assays combined with immunofluorescent staining to visualize MMP2 and MMP9 positive invadopodia. Remarkably, both MDA‐MB‐231 (Figure 5A) and SKBr3 (Figure 5B) cells transduced with Myr‐oPH‐Myc displayed a blatant decrease in gelatin degradation compared to their counterparts transduced with control Myr‐Myc. To visualize invadopodia, the fluorescent gelatin channel (red) was merged with the F‐actin staining (green) to identify F‐actin‐rich invadopodia within the confines of degraded gelatin space (green structures outlined by dotted black lines), as opposed to non‐invadopodia structures positive for F‐actin above non‐degraded gelatin (yellow structures). Notably, MDA‐MB‐231 and SKBr3 cells expressing control Myr‐Myc formed MMP2 and MMP9 positive invadopodia, respectively, as shown by overlaying MMP2 and MMP9 staining with F‐actin‐labeled invadopodia (Figure 5A,B; white structures; blue arrowheads). Quantification of invadopodia abundance, determined as the fraction of F‐actin positive protrusions over degraded gelatin space, per cell, indicated that both MDA‐MB‐231 and SKBr3 cells expressing the obscurin PH‐domain extended significantly less invadopodia (Figure 5A,B). While MMP2 and MMP9 positive puncta (magenta structures; white arrowheads) could still be visualized at the ventral surface of cells expressing Myr‐oPH‐Myc, these did not colocalize with invadopodia (Figure 5A,B).

**FIGURE 6:**
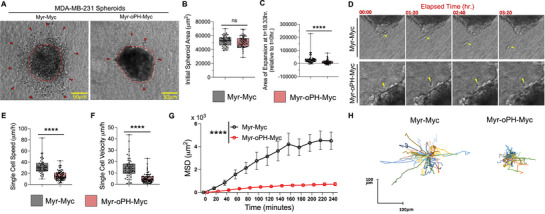
Ectopic expression of the obscurin PH‐domain halts breast cancer dissemination from spheroids. (A) Representative images of MDA‐MB‐231 Myr‐Myc or Myr‐oPH‐Myc expressing spheroids 37.5 h following embedding into 3D collagen I. Red frames indicate the spheroid edge at t = 0 h; red arrowheads denote invasion edges at t = 37.5 h. (B,C) Initial spheroid area (B) at t = 0 h and spheroid area of expansion (C) at t = 18.33 h for Myr‐Myc (*n* = 48 spheroids; 14–23 spheroids per experiment) and Myr‐oPH‐Myc (*n* = 57 spheroids; 19–20 spheroids per experiment), pooled from three independent experiments; Mann‐Whitney test. (D) Representative images of single dissociated cells (yellow arrowheads) from Myr‐Myc or Myr‐oPH‐Myc expressing spheroids during a total elapsed time of 3.33 h. (E–H) Spheroid dissociated single cell speed (µm/h; E), velocity (µm/h; F), mean square displacement (MSD;  µm^2^ x10^3^) over 4 h (G), and trajectories (H) for Myr‐Myc (*n* = 54 cells; 10–28 cells per experiment) and Myr‐oPH‐Myc (74 cells; 21–30 cells per experiment) spheroids, each pooled from three independent experiments; Mann‐Whitney test (E and F); two‐way ANOVA (G); Data are plotted as mean ± SD (B, C, E and F) or mean ± SEM (G); ^****^ *p*<0.0001.

Given the effectiveness of the obscurin‐PH domain to suppress breast cancer cell MMP expression and invadopodia formation, we asked whether it impedes invading cell dissemination, too. We recapitulated the extracellular environment of primary breast tumors by forming, embedding, and monitoring the invasive potential of MDA‐MB‐231 spheroids transduced with Myr‐Myc or Myr‐oPH‐Myc in 3D collagen I matrix (Figure 6A). Excitingly, Myr‐oPH‐Myc‐expressing spheroids displayed significantly reduced cell dissemination (Figure 6A; Video S2), as measured by the decreased area of expansion over a period of 18.3 h, compared to Myr‐Myc expressing spheroids (Figure 6B,C). Real‐time visualization of the mesenchymal, spindle‐like single cells disseminating from the edge of control spheroids confirmed their ability to successfully detach, contrary to the more rounded single cells from obscurin‐PH expressing spheroids that failed to do so (Figure 6D; Video S3). Single cells disseminating from Myr‐oPH‐Myc transduced spheroids exhibited markedly reduced speed (Figure 6E), velocity (Figure 6F), and mean square displacement (MSD, Figure 6G) relative to Myr‐Myc controls, with the former showing a consistent pattern of dissociation and reassociation with the spheroid edge, whereas the controls maintained a committed and persistent dissemination path (Figure 6H).

### 2.4 Nanoparticle Delivery of the Obscurin‐PH Domain Suppresses Breast Tumor Growth and Metastasis

Although gene transfer via adenoviral delivery or transient transfection of plasmid DNA (pDNA) is widely and effectively used for in vitro systems, the potency of either method in vivo is subpar, as they both exhibit poor tumor transduction [30]. We therefore shifted our delivery strategy to the use of lipid nanoparticles (LNPs). Formulated LNPs were optimal for systemic delivery and efficient tumor transfection [30], as indicated by their appropriate size, polydispersity index (PDI), and zeta potential charge (Figure S5A). LNP delivery of Myr‐oPH‐Myc to MDA‐MB‐231 and SKBr3 cells markedly decreased PI3K/Akt activity (Figure S5B,C) and chemotactic migration (Figure S5D,E) compared to control Myr‐Myc, replicating our findings using adenoviral infection (Figure 1B,C; Figure S2C,D). Of note, a pronounced upregulation of the total p85 levels was observed (Figure S5B,C), suggesting a robust sequestration of p85 that likely blocks protein turnover.

**FIGURE 7:**
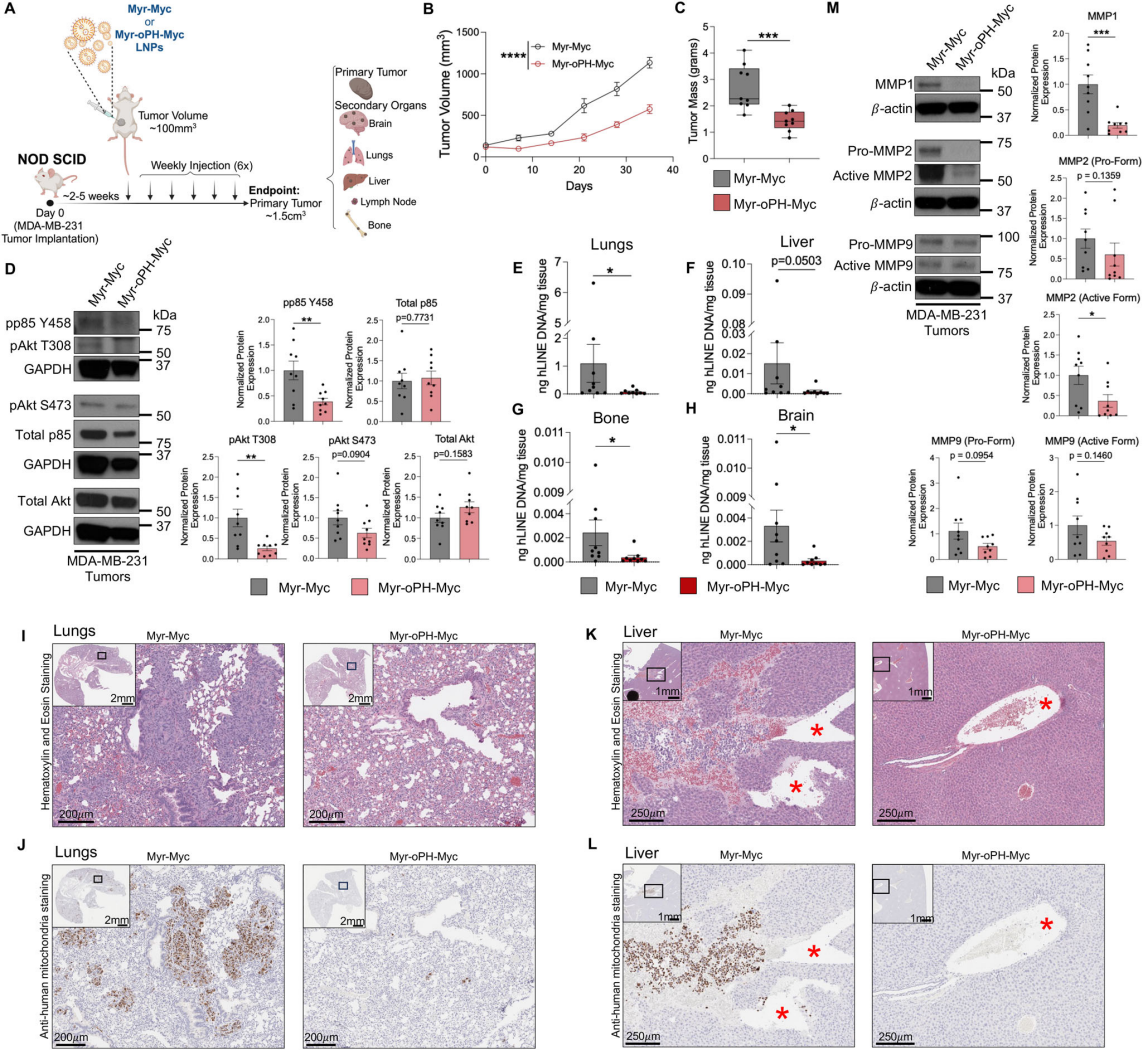
Lipid nanoparticle delivery of the obscurin PH‐domain suppresses breast cancer growth and inhibits metastasis. (A) Experimental mouse model schematic detailing the in vivo LNP treatment of orthotopic MDA‐MB‐231 tumors. 1 × 10^6^ MDA‐MB‐231 cells were injected into the mammary fat pad of female NOD SCID mice on Day 0 and were allowed to reach a volume of ∼100 mm^3^, at which point Myr‐Myc or Myr‐oPH‐Myc LNPs were administered intratumorally once a week for 6 weeks. Endpoint was specified as the point at which the primary tumor volume reached 1.5 cm^3^, followed by harvesting of the primary tumor, along with common metastatic organs, including the lungs, liver, bone, axillary lymph node, and brain; schematic created with Biorender.com (agreement number UW289RJIVN). (B) Caliper measurements of MDA‐MB‐231 tumor volumes throughout a 6‐week course of intratumoral injection of Myr‐Myc or Myr‐oPH‐Myc LNPs (*n* = 9 mice per group pooled from two independent experiments; 4–5 mice per experiment); two‐way ANOVA. (C) MDA‐MB‐231 primary tumor masses (in grams) at harvesting, following a 6‐week course of intratumoral injection of Myr‐Myc or Myr‐oPH‐Myc LNPs (n = 9 mice per group pooled from two independent experiments; 4–5 mice per experiment); two‐tailed t‐test. (D) Immunoblots of Myr‐Myc or Myr‐oPH‐Myc LNP‐treated MDA‐MB‐231 tumors for PI3K‐p85 Y458 and pAkt T308/S473 phosphorylation levels. Densitometry values are presented as expression relative to Myr‐Myc control (*n* = 9 mice per group pooled from two independent experiments; 4–5 mice per experiment); two‐tailed t‐test. (E‐H) qPCR of lungs (E), liver (F), bone (G), and brain (H) for human Long Interspersed Nuclear Element (hLINE) DNA from Myr‐Myc or Myr‐oPH‐Myc treated mice. hLINE DNA is plotted as µg hLINE DNA per mg tissue section (*n* = 9 mice per group pooled from two independent experiments; 4–5 mice per experiment); Mann‐Whitney test. (I,J) Representative hematoxylin and eosin (H & E; I) and anti‐human mitochondria (J) staining of lungs from Myr‐Myc or Myr‐oPH‐Myc treated mice. (K‐L) Representative H&E (K) and anti‐human mitochondria (L) staining of liver from Myr‐Myc or Myr‐oPH‐Myc treated mice revealing MDA‐MB‐231 tumor emboli lodged within portal sinusoids (red asterisks) in the control but not the experimental group. (M) Immunoblots of Myr‐Myc or Myr‐oPH‐Myc LNP‐treated MDA‐MB‐231 tumors for MMP1, Pro‐MMP2, MMP2, Pro‐MMP9 and MMP9. Densitometry values are presented relative to Myr‐Myc control (*n* = 9 mice per group pooled from two independent experiments; 4–five mice per experiment); Mann‐Whitney test (MMP1) and two‐tailed t‐test (Pro‐MMP2, MMP2, Pro‐MMP9, and MMP9). Data are plotted as mean ± SD (C) or mean ± SEM (B, D‐H and M); ^*^ *p*<0.05; ^**^ *p*<0.01; ^***^ *p*<0.001; ^****^ *p*<0.0001.

We proceeded to investigate the effect of LNP‐delivered Myr‐oPH‐Myc on tumor growth and metastasis. To simulate breast tumor formation and treatment in patients, MDA‐MB‐231 cells were implanted orthotopically into the mammary fat pad of NOD SCID mice and allowed to reach a volume of ∼100 mm^3^ before weekly intratumoral injections with either Myr‐Myc or Myr‐oPH‐Myc LNPs for 6 weeks (Figure 7A). qPCR at endpoint confirmed that primary tumors transduced with Myr‐Myc and Myr‐oPH‐Myc contained Myr‐Myc and Myr‐oPH‐Myc pDNA, respectively (Figure S6A,B). Likewise, RT‐qPCR showed robust expression of the Myr‐oPH‐Myc transcript in Myr‐oPH‐Myc expressing tumors (Figure S6C). Notably, Myc‐tag antibodies were unable to clearly detect Myr‐oPH‐Myc protein expression against the dense, high‐background tumor proteome, marking a limitation of this study frequently reported in the literature [31]. Growth measurements over the 6‐week injection course indicated that Myr‐oPH‐Myc LNP‐treated breast tumors grew significantly slower (Figure 7B), reaching an average mass ∼50% smaller than Myr‐Myc tumors (Figure 7C; Figure S6D). Consistent with these findings, XTT assays demonstrated a significant or trending reduction in cell viability of MDA‐MB‐231 cells expressing Myr‐oPH‐Myc delivered via adenovirus (Figure S6E) or LNPs (Figure S6F), compared to Myr‐Myc controls. Likewise, Myr‐oPH‐Myc adenoviral treatment of MDA‐MB‐231 tumorspheres growing in low‐attachment conditions profoundly decreased both the total number and size of tumorspheres formed relative to controls (Figure S6G). In agreement with our in vitro biochemical data, immunoblotting of primary tumors confirmed a significant reduction in the phosphorylation levels of p85‐Tyr458 and Akt‐Thr308, and a trending decrease in Akt‐Ser473 phosphorylation levels, indicative of the obscurin PH‐domain's suppressive effect on PI3K/Akt activity (Figure 7D). Notably, PI3K/Akt downregulation was accompanied by a trending increase in PARP cleavage (Figure S6H) in Myr‐oPH‐Myc expressing tumors, suggesting enhanced activation of intracellular apoptotic signaling.

More importantly, human long interspersed nuclear element (hLINE) qPCR of secondary organ sites revealed a dramatic reduction in metastasis to the lungs, liver, bone, and brain (Figure 7E–H) for Myr‐oPH‐Myc treated mice. However, metastasis to the axillary lymph nodes (Figure S6I) was variably decreased among Myr‐oPH‐Myc treated mice, likely reflecting the relatively advanced tumor burden at the time LNP Myr‐oPH‐Myc treatment was initiated. Furthermore, Hematoxylin and Eosin (H&E) staining (Figure 7I) along with anti‐human mitochondria (αhMito) staining (Figure 7J) and corresponding pathology review (Table S1) of Myr‐oPH‐Myc lungs revealed near normal tissue parenchyma architecture interspersed with minimal (Severity Score Grade 1) and scattered alveolar microclusters of human breast cancer metastatic cells, contrary to Myr‐Myc treated mouse lungs filled with several large (Severity Score Grade 3) alveolar, peribronchial, and peribronchiolar human breast cancer metastatic nodules. Similarly, Myr‐oPH‐Myc mouse liver contained minimal (Severity Score Grade 1) human breast cancer metastatic microclusters detached from the liver parenchyma, whereas Myr‐Myc mouse liver H&E staining (Figure 7K), αhMito labeling (Figure 7L), and accompanying pathology review (Table S1) showed sizeable human breast cancer metastatic tumor emboli (Severity Score Grade 2) lodged within feeding portal sinusoids (red asterisks). Interestingly, immunoblotting of Myr‐oPH‐Myc primary tumors demonstrated robust suppression of MMP1 and MMP2, but a non‐significant reduction of MMP9 expression (Figure 7M), in agreement with our in vitro findings of Myr‐oPH‐Myc‐treated MDA‐MB‐231 cells grown in 2‐dimension (2D) substrata (Figure 4G).

To test if the obscurin PH‐domain represses MMP9 expression in the initial stages of tumor development, we orthotopically implanted MDA‐MB‐231 cells into the mammary fat pad of NOD SCID mice, followed by 6 weekly injections of Myr‐Myc or Myr‐oPH‐Myc LNPs, 7 days post‐implantation before tumors became palpable (Figure S6J). qPCR of primary tumors at endpoint confirmed the expression of Myr‐Myc and Myr‐oPH‐Myc pDNA (Figure S6K,L), and RT‐qPCR showed abundant expression of the Myr‐oPH‐Myc transcript in the respective tumors (Figure S6M). Similar to the reduced growth of advanced Myr‐oPH‐Myc treated tumors (Figure 7B), Myr‐oPH‐Myc early tumors developed significantly slower and were ∼70% smaller compared to Myr‐Myc controls (Figure S6N). Although no metastatic burden was detected (or expected) at endpoint via hLINE DNA quantitation (Figure S6O) in either Myr‐Myc or Myr‐oPH‐Myc tumors given their early stage and small size (∼300 mm^3^ and ∼90 mm^3^, respectively), immunoblotting revealed significant suppression of MMP9 expression (Figure S6P). Consistent with this finding, cBioPortal analysis of invasive breast carcinoma patient samples from the 2024 TCGA and Genomic Data Commons dataset confirmed that MMP9 expression is higher in T1/T2 early‐stage samples relative to T3 later‐stage samples (Figure S6Q). Relatedly, analysis of the Breast Cancer METABRIC, Nature 2012 & Nature Communications 2016 datasets, demonstrated that higher MMP9 expression correlates with reduced overall survival in T1 patient samples, but not in T2 or T3 samples (Figure S6R). Thus, Myr‐oPH‐Myc LNP‐treatment induces a transient decrease in MMP9 levels during early tumorigenesis, while maintaining a prominent suppression of MMP1 and MMP2 expression in later stages.

### 2.5 The Kalirin and PLCγ1 PH‐Domains Suppress PI3K/Akt Activity, Cell Migration, and Dissemination

**FIGURE 8:**
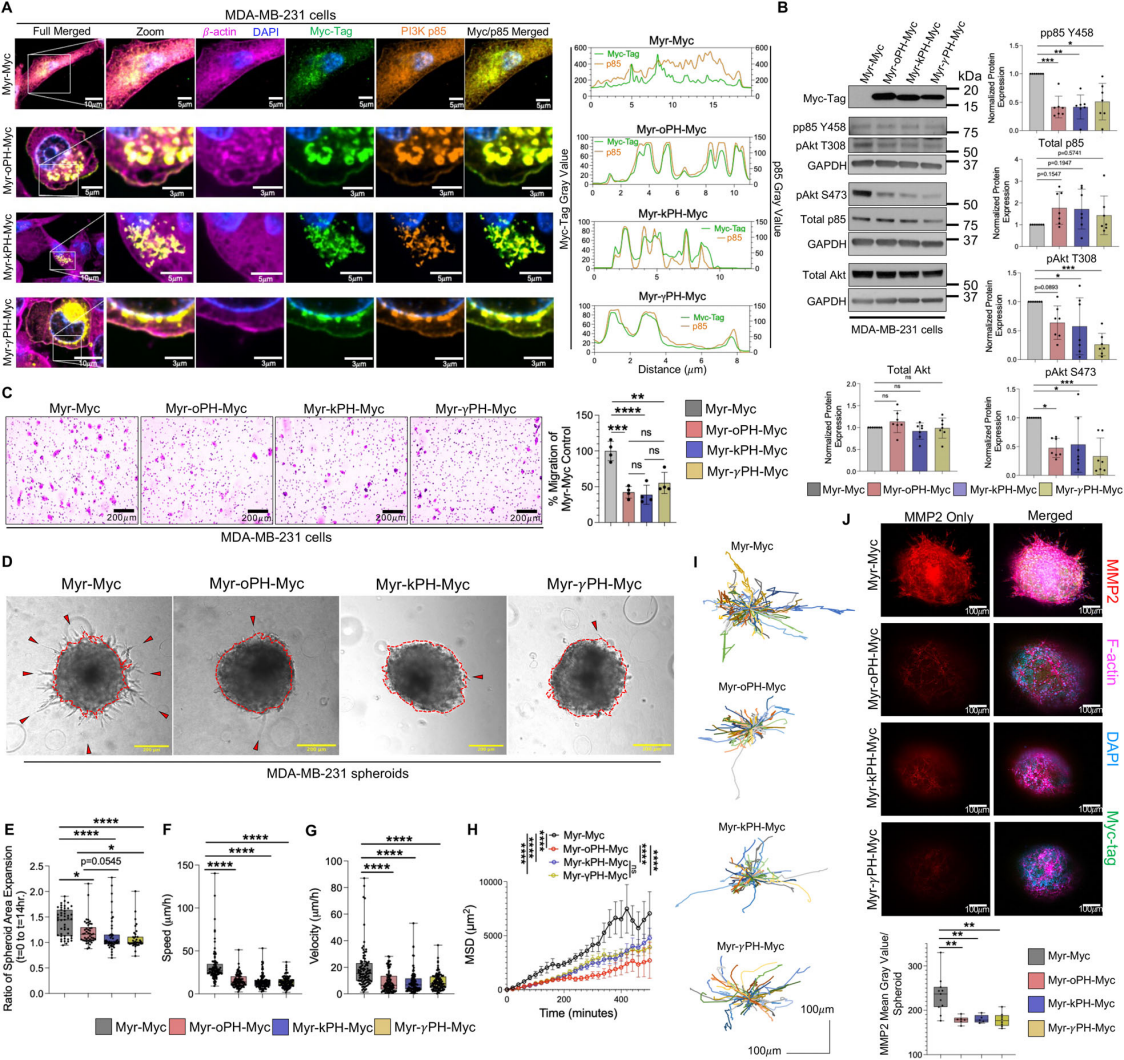
Sequestration of PI3K‐p85 by the Kalirin and PLCγ1 PH‐domains diminishes PI3K/Akt activation to block breast cancer dissemination. (A) Representative confocal images of MDA‐MB‐231 cells transiently transfected with the Myr‐Myc, Myr‐oPH‐Myc, Myr‐kPH‐Myc, or Myr‐γPH‐Myc constructs;  β‐actin (magenta), DAPI (blue), Myc‐tag (green), PI3K‐p85 (orange), and merged Myc‐tag/PI3K‐p85 (yellow). Cells of each treatment group vary significantly in size; different size scale bars allow for visualization of the entire cell. Corresponding line composite graphs display Myc‐tag and PI3K‐p85 gray values (pixel intensity). (B) Western blots of MDA‐MB‐231 cells transiently transfected with the Myr‐Myc, Myr‐oPH‐Myc, Myr‐kPH‐Myc, or Myr‐γPH‐Myc constructs, 2 h post‐serum stimulus, probed for PI3K‐p85 Y458 and pAkt T308/S473 phosphorylation levels followed by densitometric evaluation (*n* = 7 independent experiments); Kruskal‐Wallis test with Dunn's multiple comparison test (pp85‐Y458, pAkt‐S473) and one‐way ANOVA with Dunnett’ multiple comparison test (pAkt‐T308, total p85, total Akt). (C) Representative brightfield images of transwell migration assays of MDA‐MB‐231 cells transiently transfected with Myr‐Myc, Myr‐oPH‐Myc, Myr‐kPH‐Myc, or Myr‐γPH‐Myc constructs. The percent (%) migrated cells per field are plotted per treatment group (*n* = 4 independent experiments); one‐way ANOVA with Tukey's multiple comparison test. (D) Representative images of MDA‐MB‐231 spheroids treated with LNPs delivering the Myr‐Myc, Myr‐oPH‐Myc, Myr‐kPH‐Myc, or Myr‐γPH‐Myc constructs 14 h following embedding into 3D collagen I. Red frames indicate the spheroid edge at t = 0 h, while red arrowheads pinpoint invasion edges at t = 14 h. (E) Ratio of spheroid area of expansion from t = 0 to t = 14 h (*n* = 40–47 spheroids per construct, pooled from 3 independent experiments; 10–21 spheroids per experiment); Kruskal‐Wallis test with Dunn's multiple comparison test. (F–I) Spheroid dissociated single cell speed (µm/h; F), velocity (µm/h; G), mean square displacement (MSD;  µm^2^) over 8.33 h (H), and trajectories (I); *n* = 95 randomly selected spheroid single cells per construct pooled from three independent experiments (F, G, and I); *n* = 117–248 spheroid single cells per construct pooled from three independent experiments, (H); Kruskal‐Wallis test with Dunn's multiple comparison test (F,G); two‐way ANOVA followed by Tukey's multiple comparison test (H). (J) Representative confocal images of Myr‐Myc, Myr‐oPH‐Myc, Myr‐kPH‐Myc, or Myr‐γPH‐Myc LNP‐treated spheroids stained for MMP2 (red), F‐actin (magenta), DAPI (blue) and Myc‐tag (green). MMP‐2 mean gray values per spheroid are plotted (*n* = 5–10 spheroids per construct, from one independent experiment); one‐way ANOVA with Tukey's multiple comparison test; Data are plotted as mean ± SD (B,C, E–G, and J) or mean ± SEM (H); ^*^ *p*<0.05; ^**^ *p*<0.01; ^***^ *p*<0.001 ^****^ *p*<0.0001.

Given the potent anti‐metastatic properties of the obscurin‐PH domain, we examined if other PH‐domains possess similar capabilities. Using an NCBI blast search, we identified that the kalirin PH‐domain (kPH) possesses the highest sequence identity (35.4%) with the obscurin PH‐domain, while the phospholipase‐Cγ1 (PLCγ1) PH‐domain (γPH) exhibits minimal sequence homology (<1%; Figure S7A). Following the generation of myristoylated forms of the kalirin (Myr‐kPH‐Myc) and PLCγ1 (Myr‐γPH‐Myc) PH‐domains (Figure S7B), we examined their localization using transient transfection of the respective pDNA constructs in MDA‐MB‐231 cells. While Myr‐kPH‐Myc selectively concentrated to perinuclear vesicles, Myr‐γPH‐Myc targeted to the cell membrane and the perinuclear space, similar to Myr‐oPH‐Myc (Figure 8A). On the other hand, Myr‐Myc protein maintained a faint and diffuse distribution (Figure 8A). Notwithstanding their distinct distribution patterns, all three PH‐domains effectively sequestered PI3K‐p85 to their respective subcellular locations, whereas Myr‐Myc protein did not (Figure 8A). Notably, compared to their non‐myristoylated counterparts (Figure S7C), all three myristoylated PH‐domains promoted cell circularity (Figure S7D) and diminished cell area (Figure S7E), as demonstrated by β‐actin staining, which are hallmarks of indolent, non‐migratory cells with poor metastatic propensity [32]. These findings implicate that membrane targeting of all three PH‐domains elicits similar anti‐metastatic cell morphology changes.

Akin to Myr‐oPH‐Myc, transient overexpression of Myr‐kPH‐Myc and Myr‐γPH‐Myc in MDA‐MB‐231 cells significantly inhibited PI3K/Akt activity 2 h post‐serum challenge, as shown by diminished phosphorylation of p85‐Tyr458 and Akt‐Thr308/Ser473, while total levels of p85 and Akt remained unaltered (Figure 8B). Consistent with the decreased PI3K/Akt activity, MDA‐MB‐231 cells expressing either Myr‐kPH‐Myc or Myr‐γPH‐Myc exhibited reduced chemotactic migration in a Transwell assay, thereby replicating the effects observed following ectopic expression of Myr‐oPH‐Myc (Figure 8C). Relatedly, LNP delivery of Myr‐kPH‐Myc or Myr‐γPH‐Myc in MDA‐MB‐231 spheroids embedded in 3D collagen I effectively suppressed cell dissemination similar to Myr‐oPH‐Myc (Figure 8D; Video S4). This was evidenced by a notable reduction in the area of spheroid expansion over a period of 14 h, along with decreased speed, velocity, and mean square displacement of disseminating single cells relatively to Myr‐Myc control spheroids (Figures 8E–I). Consistent with these findings, immunostaining of all three PH‐transduced spheroids for MMP2 revealed drastically diminished expression compared to Myr‐Myc controls (Figure 8J).

### 2.6 Molecular Modeling of PH‐Domain Binding to the p85‐SH3 Domain

Our findings indicate that the obscurin, kalirin, and PLCγ1 PH‐domains can efficiently sequester p85 to block breast cancer cell dissemination and metastasis, while prior mechanistic work has established that the obscurin‐PH domain directly binds to the SH3‐domain of the p85 subunit with high affinity [5]. To gain insights about the structural binding interface of the PH and SH3 domains, we input each PH‐domain sequence into alpha‐fold and simulated binding with the p85‐SH3 domain (Figure S8A). Although amino acid similarity among the three PH‐domains varies from ∼34% to <1%, all three PH‐domains folded in a structurally analogous manner and interlocked with the dynamin‐binding interface of the p85‐SH3 domain, which is the canonical protein‐binding region of p85‐SH3 [33], thereby generating confidence in the validity of this model (Figure S8B). All models were of high quality, with predicted local distance difference test (pLDDT) values of 89.8, 85.2, and 90.7 for the obscurin, kalirin, and PLCγ1 PH‐domains, respectively (Figure S8C‐E); of note, high quality pLDDT values range between 80 and100 [34]. Furthermore, all three PH‐domains remained bound to the p85‐SH3 domain during each molecular dynamics simulation (50–120 ns) without significant change to the binding orientation, suggesting a stable interaction.

Given the marked similarity across the three equilibrated PH:SH3 complexes, we next inquired about potential residues that facilitate binding. In all three PH‐domains, we identified multiple residues that interlocked with a hydrophobic pocket formed by SH3 at the dynamin‐binding interface (Figure S8F–H). To test the contribution of those interactions, we performed in silico mutagenesis of various contributing PH‐domain residues, as predicted by alpha‐fold, with the intention of destabilizing the PH:SH3 complex. Bulky hydrophobic amino acids participating in relevant interactions or hydrophilic amino acids contributing to hydrogen bonds were substituted with the smaller hydrophobic alanine (A), whereas positively charged amino acids participating in electrostatic interactions were substituted with negatively charged glutamic acid (E).

Isoleucine‐23 (I23) of β‐strand 1 in the obscurin‐PH domain orchestrates hydrophobic interactions with tryptophan‐50 (W50) in the SH3 domain, while histidine‐21 (H21) of β‐strand 1 and arginine‐107 (R107) of the β‐strand 7/C‐terminal α‐helix linker (β‐7 linker) in the obscurin‐PH domain coordinate electrostatic interactions with glutamic acid‐13 (E13) of the SH3 domain (Figure S8F). Modeled mutagenesis of I23A, H21E, and R107E combinatorially induced significant motion only in the presence of the triple‐mutant, as measured by a larger radius of gyration (R_g_; Figure S9A). Furthermore, the increased overall root mean square deviation (RMSD; Figure S9B), higher root mean square fluctuation (RMSF; Figure S9C), and loss of stabilizing electrostatic interactions (Figure S9D) collectively indicate enhanced residue motion involved in complex formation. The need to induce multiple mutations to adequately disrupt the complex suggests a robust obscurin‐PH/p85‐SH3 interaction, congruent with their experimentally determined K_D_ of ∼50 nM [5].

In comparison, models of the kalirin and PLCγ1 PH‐domains complexed with the p85‐SH3 domain were more susceptible to dissociation when mutations of critical residues were modeled. Residues in analogous locations to those of the obscurin PH‐domain were predicted to support complex formation, yet with distinct variations. For instance, the kalirin‐PH/p85‐SH3 complex is dominated by hydrogen bonds between the kalirin‐PH glutamine‐22 (Q22) of β‐strand 1, E35 of *β*‐strand 2, and lysine‐101 (K101) of the β‐7 linker to the p85‐SH3 W50, threonine‐68 (T68), tyrosine‐69 (Y69), and I49, respectively (Figure S8G). In silico mutagenesis of these kalirin‐PH residues predicted that K101A, but not Q22A or E35A, induces complete dissociation of the kalirin‐PH domain from p85‐SH3 due to a profound increase in R_g_, RMSD, and RMSF, as well as a considerable loss in electrostatic interactions (Figure S9E–H). Interestingly, an increase in the total number of interdomain electrostatic interactions in both the Q22A substitution and triple‐mutant (Q22A/E35A/K101A) suggested that the polar Q22 hinders stable binding of the wild‐type, as the potent destabilizing effect of the K101A single mutant is masked by the addition of Q22A in the triple mutant (Figure S9H). Moreover, T8 in β‐strand 1 and T100 in the β‐7 linker of the PLCγ1‐PH domain form hydrogen bonds with Y69 and T100 of the p85‐SH3 domain, respectively, while the PLCγ1‐PH R23 of β‐strand 2 forms an electrostatic interaction with the p85‐SH3 E47 (Figure S8H). Mutagenesis modeling of PLCγ1‐PH R23E destabilized the complex, as seen by increased R_g_, RMSD, and RMSF values (Figure S9I–K) at the binding interface. Likewise, the T8A mutation also decreased total electrostatic interactions and somewhat destabilized the PLCγ1‐PH/p85‐SH3 interaction interface (Figure S9L).

Taken together, the obscurin‐PH/p85‐SH3 complex is predicted to demonstrate greater overall stability compared to that of the kalirin‐PH/p85‐SH3 and the PLCγ1‐PH/p85‐SH3, as determined by the minimal complex disruption induced by the triple mutant obscurin‐PH compared to the blatant complex dissociation using single mutants for kalirin and PLCγ1 PH‐domains. Importantly, our simulated mutagenesis of select residues across all three PH‐domains further predicted that disruptive substitutions in the latter half of β‐strand 1, the first half of β‐strand 2, and the β‐7 linker, destabilize interactions with either W50 or Y69 of p85‐SH3 that orchestrate PH‐SH3 binding (Figure S9M). Consequently, we jointly refer to the latter half of β‐strand 1, the first half of β‐strand 2, and the β‐7 linker of a PH‐domain as the *p*85‐*S*H3 *i*nteracting *r*egion, PSIR (Figure S9N), found in PH‐domains with p85 binding and sequestering capabilities.

### 2.7 FoldSeek Prediction of a Subfamily of PH‐Domains With p85‐SH3 Sequestering Capabilities

**FIGURE 9:**
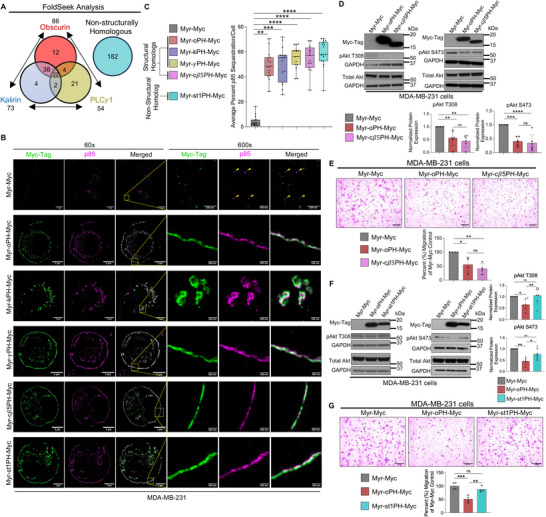
A subset of structurally homologous PH‐domains sequester PI3K‐p85, suppress PI3K/Akt activation, and block breast cancer migration. (A) Fold Seek analysis Venn diagram of the total number of PH‐domain structural homologs shared among obscurin (red), kalirin (blue), and PLCγ1 (yellow) PH‐domains, as well as non‐structurally homologous PH‐domains (cyan). (B) Representative images from STED imaging of MDA‐MB‐231 cells transiently transfected with Myr‐Myc, Myr‐oPH‐Myc, Myr‐kPH‐Myc, Myr‐γPH‐Myc, Myr‐cβ5PH‐Myc, or Myr‐st1PH‐Myc pDNA. Single MDA‐MB‐231 cells immunostained for Myc‐tag (green) and PI3K‐p85 (magenta) are shown at 60x, while zoom‐in areas at the cell periphery marked in yellow are shown at 600x, with areas of colocalization between Myc‐tag and PI3K‐p85 appearing white and PI3K‐p85 cytoplasmic puncta that do not colocalize with Myc‐Tag denoted with yellow arrowheads. (C) Plotted quantification of average PI3K‐p85 sequestration in MDA‐MB‐231 cells expressing Myr‐Myc, Myr‐oPH‐Myc, Myr‐kPH‐Myc, Myr‐γPH‐Myc, Myr‐cβ5PH‐Myc, or Myr‐st1PH‐Myc constructs (*n* = 15 cells per construct pooled from three independent experiments; five cells per experiment). (D) Western blots of MDA‐MB‐231 cells transiently transfected with Myr‐Myc, Myr‐oPH‐Myc, or Myr‐cβ5PH‐Myc, 2 h post‐serum stimulus, probed for pAkt‐T308 and pAkt‐S473 phosphorylation levels followed by densitometric evaluation (*n* = 6 independent experiments); one‐way ANOVA with Tukey's multiple comparison test. (E) Representative brightfield images of transwell migration assays of MDA‐MB‐231 cells transiently transfected with Myr‐Myc, Myr‐oPH‐Myc, or Myr‐cβ5PH‐Myc constructs. The percent (%) of migrated cells per field is plotted per treatment group (*n* = 4 independent experiments); one‐way ANOVA with Tukey's multiple comparison test. (F) Western blots of MDA‐MB‐231 cells transiently transfected with Myr‐Myc, Myr‐oPH‐Myc, or Myr‐st1PH‐Myc constructs, 2 h post‐serum stimulus, probed for pAkt‐T308 and pAkt‐S473 phosphorylation levels followed by densitometric evaluation (*n* = 7 independent experiments); Kruskal Wallis test with Dunn's multiple comparison test. (G) Representative brightfield images of transwell migration assays of MDA‐MB‐231 cells transiently transfected with Myr‐Myc, Myr‐oPH‐Myc, or Myr‐st1PH‐Myc constructs. The percent (%) of migrated cells per field is plotted per treatment group (*n* = 4 independent experiments); one‐way ANOVA with Tukey's multiple comparison test. Data is plotted as mean ± SD; ^*^ *p*<0.05; ^**^ *p*<0.01; ^***^ *p*<0.001 ^****^ *p*<0.0001.

Our experimental findings and modeling data demonstrate that, despite sharing low sequence homology, the obscurin, kalirin and PLCγ1 PH‐domains share structural homology enabling them to bind and sequester p85, thereby suppressing breast cancer cell migration and dissemination. This raises the question of whether additional PH‐domains possess similar properties. Using the FoldSeek platform [35], we screened the 285 known human PH‐domains and identified 103 structurally interrelated PH‐domains, including 22 PH‐domains that share structural homology with each of the obscurin, kalirin and PLCγ1 PH‐domains (Figure 9A). Conversely, the remaining 182 PH‐domains were found to be structurally distinct from each of these three PH‐domains (Figure 9A). We therefore hypothesized that the subgroup of 22 PH‐domains contains additional members of a previously elusive subfamily of PH‐domains capable of sequestering and inhibiting p85 activity. To identify such members, we examined whether the presence of PSIR alone is sufficient to sequester and inhibit p85, block downstream PI3K/Akt activation, and suppress breast cancer metastatic phenotypes. Using alpha‐fold, we analyzed the predicted binding orientations of the 22 structurally homologous PH‐domains with p85‐SH3 and selected to test the Centaurin‐β5 PH‐domain, expected to bind the dynamin binding interface of p85‐SH3 using the PSIR (Figure S10A). In parallel, we chose the Signal Transducing Adaptor Family Member 1 (STAP‐1) PH‐domain identified by FoldSeek within the 182 non‐structural homologs, which is also anticipated to bind the p85‐SH3 dynamin binding interface using the PSIR (Figure S10B). Of note, all five PH‐domains—including the four structurally homologous obscurin, kalirin, PLCγ1, and Centaurin‐β5 (Figure S10C–F) and the non‐structurally homologous STAP‐1 (Figure S10G)—are predicted to bind the conserved p85‐SH3 W50 and/or Y69 residues identified from our molecular dynamics simulations (Figure S9M).

To test our modeling predictions, we generated myristoylated constructs of the Centaurin‐β5 (Myr‐cβ5PH‐Myc) and STAP‐1 (Myr‐st1PH‐Myc) PH‐domains. These were subsequently transfected into MDA‐MB‐231 cells to assess their ability to sequester p85 using Stimulated Emission Depletion (STED) microscopy, which enables visualization of spatial localization at a resolution of ∼30 nm. In parallel experiments, we evaluated Myr‐oPH‐Myc, Myr‐kPH‐Myc, and Myr‐γPH‐Myc, which were shown to sequester and co‐distribute with p85 at the cell membrane and/or perinuclear vesicles, under confocal optics (Figure 5A). Consistent with our findings using confocal microscopy, STED imaging indicated that Myr‐oPH‐Myc, Myr‐kPH‐Myc, and Myr‐γPH‐Myc localized in close proximity with p85 at the cell membrane (Figure 9B) and/or perinuclear vesicles (Figure S11A), indicative of their association. In contrast, control Myr‐Myc protein maintained a diffuse, punctate distribution that did not overlap with p85 (Figure 9B; Figure S11A). Importantly, the structurally homologous Myr‐cβ5PH‐Myc and the structurally non‐homologous Myr‐st1PH‐Myc, both predicted to bind p85‐SH3 using the PSIR, effectively sequestered p85 at the cell membrane (Figure 9B) and intracellular vesicles (Figure S11A). Quantification of the proximal distribution of the individual PH‐domains and p85 revealed that ≥50% of p85 was sequestered to the cell membrane and/or intracellular vesicles, compared to <10% in the presence of control Myr‐Myc (Figure 9C). Collectively, our analysis reveals that PSIR‐containing PH‐domains, regardless of overall structural homology, sequester ≥50% of intracellular p85 to the cell membrane and perinuclear vesicles.

Given the intriguing pattern of p85 sequestration by PSIR‐containing PH‐domains, independent of the presence or absence of overall structural homology, we next investigated the ability of Myr‐cβ5PH‐Myc and Myr‐st1PH‐Myc to inhibit PI3K/Akt activity and suppress metastatic phenotypes. Immunoblotting analysis revealed that ectopic expression of the structurally homologous Myr‐cβ5PH‐Myc reduced p85‐Tyr458 (Figure S11B) and Akt‐Thr308/Ser473 (Figure 9D) phosphorylation and suppressed collective cell migration (Figure 9E), similar to Myr‐oPH‐Myc. Remarkably though, ectopic expression of the non‐structurally homologous Myr‐st1PH‐Myc failed to decrease p85‐Tyr458 (Figure S11C) and Akt‐Thr308/Ser473 (Figure 9F) phosphorylation, relative to Myr‐Myc control, nor did it dampen Transwell cell migration (Figure 9G).

Taken together, these findings indicate that PH‐domains predicted to bind p85‐SH3 using the PSIR (i.e., obscurin, kalirin, PLCγ1, Centaurin‐β5, and STAP‐1) are able to sequester p85. However, the PSIR alone is not sufficient to inhibit p85 activity and suppress downstream Akt phosphorylation, thereby proving ineffective against quelling pro‐metastatic cell migration. These findings implicate the existence of additional structural features beyond the PSIR, conserved among structurally homologous PH‐domains (i.e., obscurin, kalirin, PLCγ1, and Centaurin‐β5), that translate p85 binding and sequestration into functional p85 inhibition and downstream suppression of PI3K/Akt activity, conferring anti‐metastatic properties.

### 2.8 Identification of the “*p*85 Inhibitory And Metastasis *S*uppressor” (PIMS) Motif Within a Subfamily of PH‐Domains with Anti‐Metastatic Potential

**FIGURE 10:**
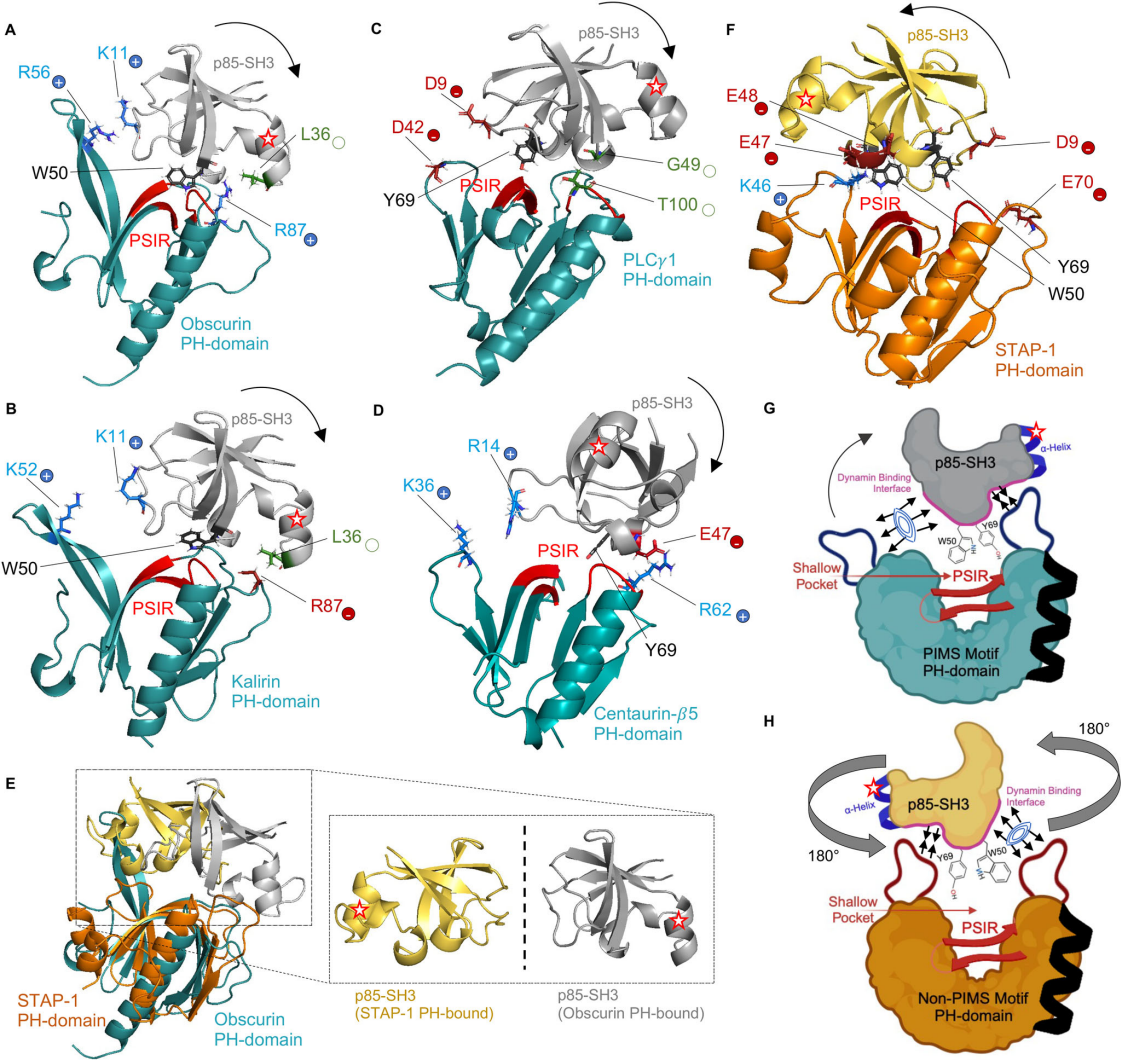
Alpha‐fold modeling of structurally and non‐structurally homologous PH‐domains predicts the *p*85 *i*nhibitory *m*etastasis *s*uppressor (PIMS) motif. (A‐D) Equilibrated alpha‐fold models of the structurally homologous obscurin (A), kalirin (B), PLCγ1 (C), and Centaurin‐β5 (D) PH‐domains (bottom subunit, cyan) in complex with the PI3K p85‐SH3 domain (top subunit, gray); the PSIR is shown in red; p85‐SH3 recipient hydrophobic (black), negatively‐charged (red), positively‐charged (blue), and neutral (green) residues on PH‐domains are highlighted; the p85‐SH3 directional tilt is denoted with a black arrow. (E,F) Overlayed alpha‐fold models of obscurin‐PH (bottom subunit, cyan) and STAP‐1‐PH (bottom subunit, orange) in complex with p85‐SH3 (top subunit; gray for obscurin‐PH and yellow for STAP‐1‐PH) are shown in (E); zoomed‐in depictions of the obscurin‐PH:p85‐SH3 and STAP‐1‐PH:p85‐SH3 models reveal that the STAP‐1‐PH:p85‐SH3 complex is the mirror image of the obscurin‐PH:p85‐SH3 complex. This is further depicted in the equilibrated stand‐alone STAP‐1‐PH:p85‐SH3 model (F). (G‐H) Summary cartoon of PH‐domains highlighting the presence (G; cyan) or absence (H; orange) of the “p85 inhibitory metastasis suppressor” (PIMS) motif; the p85‐SH3 dynamin‐binding interface is outlined in magenta, with the conserved p85‐SH3 α‐helix shown in blue and marked by a red‐and‐white star; the PH‐domain PSIR is depicted in red, while the conserved PH‐domain α‐helix is marked in black; the p85‐SH3 directional tilt in (G) is indicated with a black arrow, while the p85‐SH3 directional tilt and rotation in (H) are denoted with gray arrows. Schematic created with BioRender.com (agreement number BI289RK0DW).

To identify the structural elements, outside the PSIR, of a PH‐domain that enable effective p85 binding, sequestration, and inhibition, we further analyzed the alpha‐fold prediction models of the five PH‐domain/p85‐SH3 complexes. While the obscurin‐PH/p85‐SH3 (Figure 10A), kalirin‐PH/p85‐SH3 (Figure 10B), PLCγ1‐PH/p85‐SH3 (Figure 10C), and Centaurin‐β5‐PH/p85‐SH3 (Figure 10D) complexes adopt highly similar binding orientations, the STAP‐1‐PH/p85‐SH3 complex displays a mirror‐image inversion, as shown when overlaid with the obscurin‐PH/p85‐SH3 complex (Figure 10E). Closer evaluation of local interacting residue charges (negatives in red, positives in blue, neutrals in green) reveals that the loops surrounding the PSIR hydrophobic pocket (red peptide backbone) of the STAP‐1 PH‐domain (Figure 10F) differ substantially from those of the obscurin, kalirin, PLCγ1, and Centaurin‐β5 PH‐domains (Figure 10A–D). Consequently, alpha‐fold predicts that p85‐inhibiting PH‐domains retain a structurally conserved topology we herein coin the “p85 inhibitory metastasis suppressor” (PIMS) motif. This motif is composed of the PSIR plus leftward‐oriented repulsive charges that pivot p85‐SH3 binding rightward toward the PH‐domain PSIR, ultimately positioning the p85‐SH3 α‐helix (blue, marked with a red and white star) above the PH‐domain α‐helix (black; Figure 10G). Accordingly, obscurin‐PH's R56 is positioned against p85‐SH3's K11, kalirin‐PH's K52 is positioned against p85‐SH3's K11, PLCγ1‐PH's D42 is positioned against p85‐SH3's D9, and Centaurin‐β5‐PH's K36 is positioned against p85‐SH3's R14 (Figures 10A‐D). In contrast, the STAP‐1 PH‐domain, lacks the PIMS motif and instead harbors rightward‐oriented repulsive charges (i.e., STAP‐1 E70 against p85‐SH3 D9) and leftward‐oriented attractive charges (i.e., STAP‐1 K46 with p85‐SH3 E47 and E48) that rotate p85‐SH3 by 180° (Figures 10E‐F), resulting in a mirror‐image, functionally ineffective binding of p85‐SH3 to the PSIR (Figure 10H).

Using the newly defined PIMS motif, we re‐screened all 103 structurally homologous PH:p85‐SH3 model complexes predicted by alpha‐fold. Our screen revealed five additional PH‐domains that contain the PIMS motif, deposited in https://figshare.com/s/471195da640504d9fec0, including the PH‐domains of Neurofibromin 1 (NF1), RasGAP‐activating‐like protein 1 (RASAL1), 1‐Phosphatidylinositol 4,5‐Bisphosphate Phosphodiesterase Beta‐1 (PLCB1), TBC1 Domain Family Member 2A (TBC1D2A), and TBC1 Domain Family Member 2B (TBC12B; Table S2).

Taken together these findings indicate the following; First, the PH‐domain PSIR region binds the p85‐SH3 dynamin binding interface and sequesters p85; however, the PSIR alone does not guarantee downstream suppression of PI3K/Akt activity or blockade of pro‐metastatic phenotypes. Secondly, the PH‐domain PIMS motif consists of the PSIR and critical flanking loop residues that effectively position p85‐SH3 to suppress p85 activity. Lastly, a distinct subfamily of nine PH‐domains harbors the PIMS motif, enabling p85 binding in a uniquely defined orientation that may potently inhibit p85, suppress downstream Akt signaling, and thereby impede breast cancer metastasis.

## 3 Conclusions

Metastasis remains the leading cause of cancer‐related death, with metastatic breast cancer carrying a dismal 5‐year survival rate of ∼32%, compared to 99% for localized disease [36]. Earlier evidence had implicated *OBSCN* loss in the development and predisposition to different cancer types due to its high mutational prevalence and epigenetic dysregulation, resulting in loss of the obscurin transcript and protein [1, 37]. In breast cancer, *OBSCN* exhibits a mutational frequency of ∼12.5% and a significant degree of hypermethylation (average β‐value of >0.8 in a 0–1 scale), correlating with reduced *OBSCN* expression [1, 7]. Relatedly, prior studies have identified *OBSCN* loss as a key driver of breast cancer formation, metastatic progression, as well as chemotherapy and radiotherapy resistance [3, 4, 5, 6, 7, 8, 9]. As such, analysis of Kaplan‐Meier datasets indicates that low *OBSCN* levels correlate with significantly reduced overall survival and relapse‐free survival (RFS) in breast cancer patients [1, 4]. Most recently, our group demonstrated that restoration of *OBSCN* expression via *CRISPR*‐activation in highly metastatic TNBC cells blocks primary tumor metastasis, solidifying *OBSCN* as a potent metastasis suppressor in normal breast epithelium [4].

Herein, we harness obscurin's anti‐metastatic function—effectively addressing the challenge posed by the substantial size of the full‐length protein (720–870 kDa)—to convert its metastasis‐suppressor property into a novel, deliverable gene therapy. Our earlier biochemical studies linked obscurin loss to upregulation of the PI3K/Akt axis [5], which is altered in >40% of invasive breast carcinomas [11], and inexorably associated with apoptotic resistance, growth, enhanced migration and invasion, angiogenesis, and hormonal/chemotherapy resistance [5, 10, 14, 15, 16]. We previously established that the obscurin PH‐domain binds the PI3K/p85 SH3‐domain with a K_D_ of ∼50 nM to mediate obscurin‐p85 complex formation [5], denoting that obscurin may act upstream of the PI3K/Akt axis to regulate its activity in normal breast epithelium. In this study, we built on these findings to ectopically express a mini‐obscurin ∼50 times smaller than the full‐length obscurin protein, suppressing the PI3K/Akt axis and reinstating obscurin's metastasis suppressor function. Intriguingly, we further replicate the anti‐metastatic phenotype of the obscurin PH‐domain using three additional PH‐domains related by their structural homology as opposed to sequence homology. In turn, we provide evidence for a subclass of PH‐domains that harbor the structurally conserved PIMS motif and share anti‐metastatic capabilities.

Two PI3K inhibitors–Alpelisib and Inavolisib–targeting the catalytic p110*α* subunit encoded by the *PIK3CA* gene are currently approved for use in *PIK3CA*‐mutant, hormone receptor positive breast cancer that has failed first line hormonal therapy [38]. However, TNBC and HER2+ subtypes employ a combination of intrinsic and adaptive resistance mechanisms to bypass p110α inhibition and maintain PI3K/Akt pathway signaling, including *PIK3CA* amplification and recruitment of p110β, which dimerizes with p85 effectively substituting p110α [39]. Moreover, aggressive breast cancers with *PTEN* loss and/or hyperphosphorylated Insulin‐like Growth Factor 1 Receptor (IGF‐1R) primarily employ p110β to sustain PI3K/Akt signaling activity [39]. As such, no p110α inhibitors have been approved for the treatment of metastatic TNBC and HER2+ subtypes, both of which are associated with poorer prognosis and lower survival rates. Our work addresses this critical clinical deficiency by demonstrating that the obscurin PH‐domain serves as an effective PI3K inhibitor in metastatic TNBC and HER2+ subtypes by sequestering the PI3K/p85 regulatory subunit. Importantly, we show that obscurin‐PH mediated sequestration of p85 preserves its association with p110*α*, thereby stabilizing an inactive heterodimeric p85/p110*α* complex and resulting in reduced downstream Akt signaling. Given that p85 serves as a redundant binding partner of all Class I PI3K isoforms, including p110β, which can circumvent p110α inhibition, sequestration of p85 within an inactive heterodimeric complex is predicted to reduce the availability of free p85 required for compensatory p110*β* signaling. Consequently, obscurin‐PH targeting of p85 overcomes the primary intrinsic and adaptive resistance mechanisms of the aggressive TNBC and HER2+ subtypes, which tend to replace one Class I PI3K isoform for another to evade p110α inhibition.

No therapies currently target p85 for breast cancer treatment. Yet, like *PIK3CA*, the *PIK3R1* gene, encoding the PI3K p85 regulatory subunit, is frequently altered in breast cancer. Accordingly, ∼23% of breast cancers exhibit *PIK3R1* copy number loss while ∼28% harbor *PIK3R1* mutations identified as hemizygous losses [40]. Furthermore, transcriptomic analysis showed a 50–77% reduction in *PIK3R1* mRNA expression in breast cancer samples relative to healthy controls [40]. This evidence has implicated *PIK3R1* as a tumor suppressor, underscoring its relevance as a critical therapeutic target [40]. Our data support a model in which the obscurin PH‐domain suppresses PI3K/Akt signaling through a two‐pronged mechanism. In its non‐phosphorylated state, p85 through its central iSH2 coiled‐coil region clamps p110*α*, maintaining autoinhibition [18]. Phosphorylation of Tyr458 within the iSH2 coiled‐coil by scaffolding kinases such as Src‐family members—facilitated by the p85 SH3‐domain [41]—loosens this clamp, promoting a conformational change in p110*α* that enhances PI3K holoenzyme catalytic activity [18]. Our findings show that ectopically expressed obscurin PH‐domain binds the p85 SH3‐domain via the PIMS motif, reducing Tyr458 phosphorylation and downstream Akt signaling. This evidence supports the first arm of our model, in which obscurin‐PH binding may hinder SH3‐domain mediated kinase recruitment and access to the iSH2 coiled‐coil, preventing Tyr458 phosphorylation, thereby stabilizing the iSH2 autoinhibitory clamp to lock the p85/p110*α* complex in an inactive state. Moreover, prior studies have shown that *PIK3R1* knockdown enhances PI3K signaling by depleting the pool of inactive p85 monomers that compete for and quench receptor tyrosine kinase (RTK) binding sites, permitting increased active p85/p110*α* heterodimer recruitment and signaling [40]. Our data demonstrate that delivery of the obscurin PH‐domain increases the total intracellular p85 levels, while potently inhibiting PI3K/Akt signaling. This supports the second arm of our model, in which obscurin‐PH/p85‐SH3 binding may sterically compete with E3 ubiquitin ligases, which also bind to the p85‐SH3 domain [42], preventing degradation and proteasomal recycling of the catalytically silent PI3K holoenzyme. In turn, the enzymatically inactive heterotrimer accumulates to saturate RTK binding sites, blocking downstream signaling to Akt. Thus, our model supports a possible dual role for the obscurin PH‐domain as an allosteric molecular gatekeeper, preventing p85‐Tyr458 phosphorylation, and as an inhibitor of proteasomal recycling of the inactive PI3K holoenzyme. Together, these actions may promote the accumulation of a heterotrimer, composed of obscurin‐PH and a catalytically silent p85/p110*α* holoenzyme, which effectively quenches intracellular RTK signaling.

Migratory breast cancer cells recruit p85/p110 complexes to activated receptors at the leading edge, generating local phosphatidylinositol (3,4,5)‐trisphosphate (PIP_3_) gradients that activate small GTPases (e.g., Cdc42), which nucleate branched actin filaments to extend filopodia and invadopodia, facilitating migration and invasion [14, 43]. Myristoylation of obscurin‐PH traffics it to the cell membrane and perinuclear vesicles, mirroring the native distribution of obscurin in breast epithelial cells, and driving a profound transformation in the actin cytoskeleton. Membrane‐bound obscurin‐PH induces cell shrinkage, increases circularity, and leads to complete ablation of filopodia and invadopodia. These effects coincide with a continuous, ring‐like distribution of the obscurin‐PH/p85 complex at the cell membrane, dispersing p85 clusters from the leading edge where they promote nucleation of actin‐based protrusions [14, 43]. Supraphysiologic doses of bradykinin hyperactivate the Bradykinin B2 G protein‐coupled receptor (B2R) and Gα12/13, bypassing PI3K, to directly activate Cdc42 without local PIP_3_ gradients [25, 44]. Our data show that the same bradykinin dosing partially rescues filopodia formation, migration, and adhesion in obscurin‐PH expressing breast cancer cells, thereby directly linking obscurin‐PH‐mediated inhibition of migration and adhesion to filopodia abrogation. In addition, these findings support a model in which obscurin‐PH‐mediated PI3K suppression may block actin remodeling and filopodia formation via the downstream Cdc42 effector. Given that PI3K signals to Cdc42 through spatially localized PIP3 gradients, and that sequestered p85 is evenly distributed across the cell membrane without polarization to the leading edge, obscurin‐PH likely disrupts spatial signaling to Cdc42, thereby forcing a non‐polarized distribution of the actin cytoskeleton. Thus, unlike small molecule PI3K inhibitors that diffuse through the cytosol to inhibit p110 at its local site of action—including polarized edges—myristoylated obscurin‐PH enforces spatial rewiring by sequestering p85 and impeding localized PI3K signaling to Cdc42, cytoskeletal actin polarity, and filopodia/invadopodia formation.

Matrix metalloproteinases (MMPs) are potent transmembrane and soluble enzymes transcriptionally regulated by PI3K/Akt‐dependent transcription factors that enable highly metastatic breast cancers to degrade ECM barriers during intravasation and extravasation [12, 13, 19, 45]. Our analysis of patient datasets, corroborated by our in vitro studies, revealed that obscurin PH‐domain treatment induces a marked, subtype‐specific reduction in MMP expression. Prior work has shown that different breast cancer molecular subtypes harbor distinct patterns of chromatin accessibility at MMP gene loci, resulting in variable subtype‐specific MMP expression profiles [46], despite shared transcriptional regulation by NF‐κB, AP‐1, and STAT3 [45, 47]. As PI3K/Akt signaling has been shown to directly drive NF‐κB activation to promote MMP expression and breast cancer metastasis [45], these data support a schema in which ectopic obscurin‐PH expression represses the PI3K/Akt/NF‐κB axis to silence subtype‐specific, endogenously expressed MMPs with open chromatin states. In turn, independent of the distinct MMP epigenetic landscape and expression profile of each breast cancer molecular subtype, the obscurin PH‐domain is able to uniformly repress MMP expression. This paradigm is further substantiated by our evaluation of MMP expression in early‐ and late‐stage triple‐negative, claudin‐low in vivo tumors treated with obscurin PH‐domain LNPs. Following pre‐established trends that early‐ and late‐stage primary tumors carry evolving MMP chromatin landscapes [46], we observed stage‐specific MMP suppression by the obscurin PH‐domain that parallels the fluctuating stage‐specific chromatin state of MMPs witnessed within the same breast cancer molecular subtype. These findings support the remarkable ability of the obscurin PH‐domain to overcome molecular subtype‐specific and stage‐dependent epigenetic control of MMP expression to achieve consistent silencing of the invasive breast cancer MMP milieu.

Most breast cancers are diagnosed prior to detectable metastatic disease, presenting a critical window during which metastasis‐targeted interventions could have maximal impact [36]. However, standard‐of‐care therapies for metastatic TNBC and HER2+ subtypes rely heavily on non‐specific cytotoxic agents that target proliferating cells, leaving the metastatic cascade largely unaddressed [48]. Ironically, recent evidence demonstrates that metastatic cancer cells undergoing confined migration exhibit low proliferative activity, upregulated drug‐efflux pumps, and increased stem‐like transcriptional programs, driving resistance to conventional chemotherapies [49]. As a result, proliferative primary tumor breast cancer cells respond dramatically to cytotoxic agents, while disseminated metastatic cells remain largely unscathed [49]. Consequently, optimal breast cancer therapeutic interventions would combine tumor cell eradication with direct targeting of metastatic progression. Our findings demonstrate that local delivery of LNPs carrying the obscurin PH‐domain to mice with advanced‐stage orthotopic triple‐negative breast tumors slowed primary tumor growth and robustly suppressed metastasis to all major secondary organ sites, except the lymph nodes where suppression was variable. Notably, ∼25.6% of breast cancer patients without distant disease already harbor lymph node metastases at diagnosis, underscoring lymphatic spread as an early event in breast cancer metastatic dissemination [50]. We therefore attribute the minimal difference in nodal metastases in our model to the late stage at which treatment was initiated. We anticipate that earlier administration of obscurin‐PH, aligned with the typical timing of breast cancer diagnosis, would further prevent lymphatic dissemination and comprehensively block systemic spread.

PH‐domains are best known for their canonical binding to PIPs, while their capacity to mediate protein–protein interactions remains comparatively underexplored [51]. Through our investigation of the obscurin PH‐domain, we confirmed three additional PH‐domains (kalirin, PLCγ1, and Centaurin‐β5) that replicate equivalent anti‐metastatic capabilities in highly aggressive TNBC cells by sequestering p85, expanding our discovery of one potent anti‐metastatic therapeutic to at least four. Structural modeling, molecular dynamics simulations, and in silico mutagenesis studies converged on the conserved PSIR that mediates PH‐domain/p85‐SH3 binding. In turn, high‐resolution STED microscopy, signaling studies, and functional assays in combination with structural modeling revealed that the PSIR is part of a larger PIMS motif, which uses charged residues within flanking loops to orient the p85‐SH3‐domain over the PSIR, thereby translating p85 sequestration to p85 inhibition, and downstream PI3K/Akt suppression and pro‐metastatic blockade. Realtedly, all four tested PH‐domains containing the PIMS motif increased total p85 levels, amounting to decreased relative p85‐Tyr458 phosphorylation and subsequent attenuation of Akt signaling. Systematic screening of the 103 interrelated PH‐domains identified by FoldSeek for the presence of the PIMS motif uncovered five additional PH‐domains (Table S2) that share this distinctive p85‐SH3 binding topology. Thus, when expressed in isolation from the structural confinements of their parent protein, this subgroup of nine PH‐domains may exert anti‐metastatic effects equivalent or superior to current PI3K chemical inhibitors, pointing to a new direction for metastasis‐targeted therapies.

Collectively, our work unveils a previously elusive class of non‐chemical PI3K inhibitors in the form of the PH‐domain with potent anti‐metastatic properties, addressing an urgent unmet clinical need. The mechanism by which obscurin‐PH blocks breast cancer metastasis is multifaceted; our unique construct quenches PI3K catalytic activity to dampen downstream PI3K/Akt signaling, rewires cytoskeletal actin polarity to block filopodia and invadopodia formation, and silences invasive MMP expression. Critically, we put forth not one, but nine variants of this novel class of PI3K inhibitors, leveraging their unique p85‐sequestration and inhibition capabilities through the structurally conserved PIMS motif, thereby amplifying our discovery of novel anti‐metastatic agents. Together, these findings pave the way for new targeted therapies to counter breast cancer metastatic progression and improve patient survival.

## 4 Experimental Section/Methods

### 4.1 Human Cell Lines and Cell Culture

MCF10A (RRID:CVCL_0598), MDA‐MB‐231 (RRID:CVLC_0062), and SKBr3 (RRID:CVLC_0033) cells were purchased from ATCC (Manassas, Virginia). MCF10A cells were cultured as previously described [5]. MDA‐MB‐231 cells were maintained in DMEM supplemented with 10% FBS and 1% penicillin‐streptomycin, while SKBr3 cells were maintained in McCoys 5A Modified Medium supplemented with 10% FBS and 1% penicillin‐streptomycin. All cells were cultured in 5% CO2 at 37°C in a humidified tissue culture incubator. Cells were routinely checked for Mycoplasma contamination using the MycoGuard Mycoplasma PCR Detection Kit (Genecopoeia, Rockville, MD), according to the manufacturer's protocol, and were confirmed to be free of contamination. All cells were used between passages 8 and 22.

### 4.2 Cloning and Construct Generation

#### 4.2.1 Myr‐Myc Construct

The myristoylation (Myr) sequence (5’‐ATGGGAAGCAGCAAGAGCAAGCCAAAG‐3’) was ligated into the pCMV6‐Entry Mammalian Expression Vector (OriGene, Rockville, MD) as annealed overlapping oligos (Table S3) at AscI and XhoI sites (NEB, Ipswich, MA), in frame with the COOH‐terminal Myc and DDK tags and transformed into *E. coli* cells. The Myr‐Myc sequence was then PCR‐amplified using OneTaq 2X Master Mix (NEB, NEB, Ipswich, MA) and ligated into the pCMV6‐Entry Mammalian Expression Vector (OriGene, Rockville, MD) at AscI and PmeI sites (NEB, Ipswich, MA), introducing a stop codon prior to the DDK tag, to eliminate it. The pCMV6‐Myr‐Myc plasmid was subsequently gel‐purified using the QIAquick Gel Extraction Kit (Qiagen, Germantown, MD) and transformed into *E. coli* cells.

#### 4.2.2 Obscurin Pleckstrin Homology (PH)‐Domain constructs

The obscurin PH‐domain was cloned from cDNA reverse transcribed (SuperScriptTM First‐Strand Synthesis System for RT‐PCR, Thermo Fisher, Waltham, MA) from MCF10A RNA isolated using the RNeasy Plus Mini Kit (Qiagen, Germantown, MD). The obscurin PH‐domain cDNA was then PCR amplified (OneTaq 2X Master Mix, NEB, Ipswich, MA), ligated into the pCMV6‐Entry Mammalian Expression Vector (OriGene, Rockville, MD) at AscI and XhoI sites (NEB, Ipswich, MA), and transformed into *E. coli* cells. To eliminate the terminal DDK tag, the obscurin PH‐Myc (oPH‐Myc) was PCR‐amplified from the resultant pCMV6‐oPH‐Myc‐DDK plasmid using OneTaq 2X Master Mix (NEB, Ipswich, MA) and ligated into the pCMV6‐Entry Mammalian Expression Vector (OriGene, Rockville, MD) at AscI and PmeI sites (NEB, Ipswich, MA), introducing a stop codon prior to the DDK tag. The pCMV6‐oPH‐Myc plasmid was subsequently gel purified using the QIAquick Gel Extraction Kit (Qiagen, Germantown, MD) and transformed into *E. coli* cells. In a subsequent reaction, the Myr tag was inserted via PCR amplification of the oPH‐Myc sequence from the pCMV6‐oPH‐Myc plasmid, using a primer set that contained the Myr sequence and the OneTaq 2X Master Mix (NEB, Ipswich, MA). The obtained amplicon was subsequently ligated into the pCMV6‐Entry Mammalian Expression Vector (OriGene, Rockville, MD) at AscI and PmeI sites (NEB, Ipswich, MA), gel purified using the QIAquick Gel Extraction Kit (Qiagen, Germantown, MD) and transformed into *E. coli* cells.

#### 4.2.3 Kalirin Pleckstrin Homology (PH)‐Domain constructs

The kalirin PH‐domain sequence was purchased as a custom insert in the pUC57 donor plasmid (GenScript, Piscataway, NJ). The kalirin PH‐domain sequence was PCR amplified (OneTaq 2X Master Mix, NEB, Ipswich, MA) from the pUC57 plasmid, ligated into the pCMV6‐Entry Mammalian Expression Vector (OriGene, Rockville, MD) at AscI and XhoI sites (NEB, Ipswich, MA), and transformed into *E. coli* cells. To eliminate the terminal DDK tag, the kalirin PH‐Myc (kPH‐Myc) sequence was then PCR‐amplified from the resultant pCMV6‐kPH‐Myc‐DDK plasmid vector using OneTaq 2X Master Mix (NEB, Ipswich, MA) and ligated into the pCMV6‐Entry Mammalian Expression Vector (OriGene, Rockville, MD) at AscI and PmeI sites (NEB, Ipswich, MA), introducing a stop codon prior to the DDK tag. The ligated pCMV6‐kPH‐Myc plasmid was subsequently gel purified using the QIAquick Gel Extraction Kit (Qiagen, Germantown, MD) and transformed into *E. coli* cells. In a subsequent reaction, the Myr tag was introduced via PCR amplification of the kPH‐Myc sequence from the pCMV6‐kPH‐Myc plasmid, using a primer set that included the Myr sequence and the OneTaq 2X Master Mix (NEB, Ipswich, MA). The resultant Myr‐kPH‐Myc amplicon was ligated into the pCMV6‐Entry Mammalian Expression Vector (OriGene, Rockville, MD) at AscI and PmeI sites (NEB, Ipswich, MA), gel purified using the QIAquick Gel Extraction Kit (Qiagen, Germantown, MD) and transformed into *E. coli* cells.

#### 4.2.4 PLCγ1 Pleckstrin Homology (PH)‐Domain constructs

The PLCγ1 PH‐Myc and PLCγ1 Myr‐PH‐Myc sequences were purchased as custom inserts in the pUC57 donor plasmid (GenScript, Piscataway, NJ). The PLCγ1 PH‐Myc or PLCγ1 Myr‐PH‐Myc sequences were PCR amplified (OneTaq 2X Master Mix, NEB, Ipswich, MA) from the pUC57 plasmid, ligated into the pCMV6‐Entry Mammalian Expression Vector (OriGene, Rockville, MD) at AscI and XhoI sites (NEB, Ipswich, MA) introducing a stop codon prior to the DDK tag, gel purified using the QIAquick Gel Extraction Kit (Qiagen, Germantown, MD), and transformed into *E. coli* cells.

#### 4.2.5 Centaurin‐β5 and STAP‐1 Pleckstrin Homology (PH)‐Domain constructs

The Centaurin‐β5 and STAP‐1 Myr‐PH‐Myc sequences were purchased as custom inserts in the pUC57 donor plasmid (GenScript, Piscataway, NJ). Both Myr‐PH‐Myc sequences were PCR amplified (OneTaq 2X Master Mix, NEB, Ipswich, MA) from the pUC57 plasmid, ligated into the pCMV6‐Entry Mammalian Expression Vector (OriGene, Rockville, MD) at AscI and XhoI sites (NEB, Ipswich, MA) introducing a stop codon prior to the DDK tag, gel purified using the QIAquick Gel Extraction Kit (Qiagen, Germantown, MD), and transformed into *E. coli* cells.

All plasmids were transformed into One Shot TOP10 chemically competent *E*. coli cells (Thermo Fisher, Waltham, MA). All primer sets are listed in Table S3. The authenticity of all plasmids was verified by Sanger sequencing (Genewiz, South Plainfield, NJ).

### 4.3 Transient Transfection

MDA‐MB‐231 cells used for immunoblotting or transwell migration assays were plated at a 3 × 10^5^ cells/well density in 6‐well plates (Corning, Corning, NY), while cells for immunofluorescence experiments were plated at a 3 × 10^4^ cells/well density in 96‐well plates (Ibidi, Fitchburg, Wisconsin). Cells were allowed to adhere for 24 h in a humidified tissue culture incubator at 37°C, 5% CO_2_, before they were transiently transfected with the indicated plasmids, using Lipofectamine 3000 Transfection Reagent (Thermo Fisher, Waltham, MA; #L3000008). All transfections were completed in DMEM 10% FBS, 1% penicillin‐streptomycin for 24 h. Cells were allowed to recover in complete growth media for an additional 2 or 24 h before further experimentation.

### 4.4 Adenovirus Generation and Treatments

Myr‐Myc and Myr‐oPH‐Myc adenoviruses were generated by SignaGen Laboratories (Frederick, MD, USA). In brief, flanking BamHI and HindIII sites were added to the Myr‐Myc DNA sequence via PCR amplification followed by cloning into the pAD‐CMV‐RFP shuttle vector. Likewise, EcoRI and PmeI restriction sites were added to the Myr‐oPH‐Myc DNA via PCR amplification for insertion into the Ad‐CMV‐RFP shuttle vector. The resultant adenovirus shuttle plasmids were then combined into the pAD backbone and packaged into HEK293 cells, obtained by ATCC (RRID:CVCL_0045), by transfection using the LipoJet In Vitro Transfection Kit (Ver. II, #SL100468). Two weeks following transfection, adenoviruses were harvested and further amplified in HEK293 cells. Titration was performed using the Adeno Rapid Titer Kit (632250) from Takara (San Jose, CA).

For adenovirus treatments, MDA‐MB‐231 and SKBr3 cell monolayers were plated at a 3 × 10^5^ cells/well density in 6‐well plates (Corning, Corning, NY) and allowed to adhere for 24 h in a humidified tissue culture incubator at 37°C, 5% CO_2_ before infection with Myr‐Myc control or Myr‐oPH‐Myc adenovirus at a MOI of 400 in complete growth media. SKBr3 cells used specifically for transwell migration assays were plated in 100 mm tissue‐culture dishes (Corning, Corning, NY) at a 1.5 × 10^6^ cells/plate density. Twenty‐four h following treatment, media was replaced with fresh, adenovirus‐free, complete growth media, while 24 h later cells were either harvested for protein lysates or replated for immunofluorescence, gelatin invasion, transwell migration, single cell migration through microchannels, spheroid invasion, or adhesion assays.

### 4.5 Lipid Nanoparticle (LNP) Formulation and Cell Treatment

LNPs were formulated using the NanoAssemblr Ignite platform (Precision NanoSystems, Vancouver, Canada), which utilizes a Y‐shaped staggered herringbone micromixer cartridge (Precision NanoSystems, Vancouver, Canada). The lipid phase was composed of Dlin‐KC2‐DMA (2,2‐dilinoleyl‐4‐dimethylaminoethyl‐[1, 3]‐dioxolane; MedKoo Biosciences Inc., Morrisville, NC), DSPC (1,2‐distearoyl‐sn‐glycero‐3‐phosphocholine; Avanti Polar Lipids Inc., Alabaster, Alabama), cholesterol (Sigma‐Aldrich, St. Louis, MO), and mPEG2000‐DSPE (1,2‐dimyristoyl‐rac‐glycero‐3‐methoxypolyethyleneglycol‐2000; Avanti Polar Lipids Inc., Alabaster, Alabama). The lipids were dissolved in ethanol (Koptec) at a molar ratio of KC2:DSPC: cholesterol:mPEG2000‐DSPE of 50:10.5:38.1:1.5. The aqueous phase contained the desired DNA segment in water (DNase, RNase and protease tested, sterile filtered; Quality Biological, Gaithersburg, MD). A nitrogen‐to‐phosphate ratio (N/P) of 8 between the ionizable lipid and DNA was maintained for all formulations to sustain optimal charge balance and packaging of plasmid DNA into LNPs. The aqueous‐to‐lipid flow rate ratio (FRR) was maintained at 3:1 for the formulation at a flow rate of 12 mL/min. An initial waste volume of 0.45 mL and a final waste volume of 0.05 mL was set for all formulations. LNPs were purified using Amicon Ultra Centrifugal Filters (Millipore Sigma, Burlington, MA). Hydrodynamic size (z‐avg), polydispersity index (PDI), and surface charge (zeta potential) were determined by dynamic light scattering (DLS) using Malvern Zetasizer Nano‐ZS Zen 3600. Purified LNPs were diluted 100 times in water for hydrodynamic size and PDI assessments, while for zeta potential measurements LNPs were diluted 100 times in PBS. The DNA concentration after purification was measured using a NanoDrop 2000 (Thermo Fisher, Waltham, MA). All LNPs were stored at 4°C and used within 6 h of formulation.

MDA‐MB‐231 cell monolayers were plated at a 3 × 10^5^ cells/well density in 6‐well plates (Corning, Corning, NY) for western blot and transwell migration assays, while SKBr3 cell monolayers were plated at a 3 × 10^5^ cells/well density in 6‐well plates (Corning, Corning, NY) for western blot and a 1.5 × 10^6^ cells/plate density in 100 mm dishes (Corning, Corning, NY) for transwell migration assays. MDA‐MB‐231 and SKBr3 cells were allowed to adhere for 24 h in a humidified tissue culture incubator at 37°C, 5% CO_2_ before treatment with 20 µg/mL and 10 µg/mL, respectively, of LNPs carrying control Myr‐Myc, Myr‐oPH‐Myc, Myr‐kPH‐Myc or Myr‐γPH‐Myc plasmid in Opti‐MEM Reduced Serum Medium (Thermo Fisher, Waltham, MA). Twenty‐four h post‐treatment, the LNP‐containing Opti‐MEM media was replaced with fresh, particle‐free complete growth media, and 24 h later, cells were either harvested for protein lysates or replated for transwell migration assays.

### 4.6 Immunofluorescence, Confocal Microscopy, and Stimulated Emission Depletion (STED) Microscopy

For confocal microscopy, cell monolayers were plated on non‐coated or pre‐coated with the indicated substrate Polymer Coverslip μ‐Plate 96‐well Square multiwell plates (Ibidi, Fitchburg, Wisconsin). Following permeabilization with 100% methanol for 15 min at ‐20°C, cells were washed with 1X PBS, blocked in 3% bovine serum albumin (BSA) for 1 h at room temperature, re‐washed with 1X PBS, and incubated with primary antibodies for Myc‐tag (mouse monoclonal antibody 9E10; Abcam, Waltham, MA; ab32), PI3K/p85 (rabbit monoclonal antibody 19H8; Cell Signaling Technology, Danvers, MA #4257), PI3K/p110*α* (rabbit monoclonal antibody; Cell Signaling Technology, Danvers, MA #4255S), RFP (rabbit polyclonal antibody, V22R36; Thermo Fisher, Waltham, MA; PA1‐986), and β‐actin (goat polyclonal antibody, LSBio, Lynnwood, WA; LS‐B15553) overnight at 4°C in a 0.3% Triton‐X‐100, 1% BSA, 1X PBS solution. Following extensive washes with 1X PBS, cells were treated with the appropriate secondary antibodies: anti‐mouse AlexaFluor 488 (Thermo Fisher, Waltham, MA), anti‐rabbit AlexaFluor 568 (Thermo Fisher, Waltham, MA), and anti‐goat Alexa Fluor 633 (Thermo Fisher, Waltham, MA) at room temperature in the dark for 1 h. Samples were subsequently stained with DAPI (Thermo Fisher, Waltham, MA), immersed in Ibidi Immersion Oil (Ibidi, Fitchburg, Wisconsin), and stored at 4°C in the dark until imaging. Images were acquired on a Nikon W1 spinning disk with a 60x oil immersion objective, with pixel sizes at 0.06 or 0.11 µm/pixel. Line composite graphs of Myc‐Tag and p85 colocalization were generated via fluorescence pixel intensity (gray value) measurements acquired with ImageJ software (National Institutes of Health, Bethesda, Maryland), where a straight line was drawn at random across the cell body and pixel intensity of the Myc‐Tag and p85 channels (mean gray value) versus distance (µm) was plotted.

Spheroids were stained as previously described [52]. Briefly, following fixation in 4% PFA for 15 min at room temperature, spheroids were washed in 1X Dulbecco's phosphate buffered saline (D‐PBS), permeabilized with 0.5% Triton‐X‐100 in 1X D‐PBS for 1 h at room temperature, and blocked with 10% FBS, 1% BSA in 1X D‐PBS for 4 h at room temperature. Spheroids were subsequently incubated with primary antibodies against Myc‐tag (mouse monoclonal antibody 9E10; Abcam, Waltham, MA; ab32) and MMP‐2 (goat polyclonal antibody I34C660; R&D systems, Minneapolis, MN; AF902) in 1% FBS, 1% BSA, and 0.2% Triton‐X‐100 in 1X D‐PBS overnight at 4°C. Following extensive washes in 1X D‐PBS, spheroids were incubated at room temperature in the dark for 4 h with the appropriate secondary antibodies: anti‐mouse AlexaFluor 488 (Thermo Fisher, Waltham, MA) and anti‐goat AlexaFluor 594 (Thermo Fisher, Waltham, MA) in 1% FBS, 1% BSA, and 0.2% Triton‐X‐100 in 1X D‐PBS. All spheroids were subsequently counter‐stained with Alexa Fluor 647 Phalloidin (Cell Signaling Technology, Danvers, MA; #8940) for 15 min at room temperature in the dark, followed by DAPI (Thermo Fisher, Waltham, MA) for 10 min at room temperature in the dark, immersed in Ibidi Immersion Oil (Ibidi, Fitchburg, Wisconsin), and stored in 4°C in the dark for a maximum of 48 h before imaging. Images were acquired on a Nikon W1 spinning disk with a 20x air objective, with pixel sizes at 0.32 µm/pixel. Whole spheroid MMP2 expression was measured using ImageJ software (National Institutes of Health, Bethesda, Maryland), where a region of interest (ROI) was first drawn around each spheroid edge using β‐actin staining as reference, followed by measuring MMP2 fluorescence pixel intensity (mean gray value).

For stimulated emission depletion (STED) microscopy, cell monolayers were plated on fibronectin pre‐coated 1.5H Glass Coverslip μ‐Plate 96‐well Square multiwell plates (Ibidi, Fitchburg, Wisconsin). Following permeabilization with 100% methanol for 15 min at ‐20°C, cells were washed with 1X D‐PBS, blocked in 3% BSA for 1 h at room temperature, re‐washed with 1X D‐PBS, and incubated with primary antibodies for Myc‐tag (mouse monoclonal antibody 9E10; Abcam, Waltham, MA; ab32) and PI3K/P85 (rabbit monoclonal antibody 19H8; Cell Signaling Technology, Danvers, MA #4257) overnight at 4°C in a 0.3% Triton‐X‐100, 1% BSA, 1X PBS solution. Following extensive washes with 1X D‐PBS, cells were treated with the appropriate secondary antibodies: anti‐mouse Abberior Star Orange (Abberior, Göttingen, Germany) and anti‐rabbit Abberior Star Red (Abberior, Göttingen, Germany) at room temperature in the dark for 1 h. Samples were subsequently immersed in Abberior Mount Liquid AntiFade (Abberior, Göttingen, Germany), and stored at 4°C in the dark until imaging. STED images were acquired with the Abberior Facility Line STED microscope using an Olympus 60x/1.42 STED‐UPLXAPO60xO oil immersion lens. Abberior STAR Orange and STAR Red were excited at 561 nm and 640 nm, respectively, and depletion was performed with a 775 nm pulsed laser with a gating of 1–8 ns and a pixel dwell time of 5 µs. The emission from both channels was collected with the matrix detector, and the images were post‐processed with the Abberior Imspector software. Each line was scanned with 20‐line accumulations during acquisition. The pixel size was set to 30 nm, and the pinhole was set to 1 AU.

### 4.7 Stimulated Emission Depletion (STED) Microscopy Analysis

For all STED analysis, matrix post‐processed images (Abberior Imspector software) were evaluated to calculate percent (%) p85 sequestration per cell using ImageJ software (National Institutes of Health, Bethesda, Maryland). All images were initially converted to TIFF files, equally adjusted for brightness and contrast per secondary antibody controls and converted to binary images. ROIs were then drawn around the cell membrane and extraneous extracellular signal was eliminated. To visualize p85 signal that solely localized with Myc‐Tag signal, the p85 binary image was overlayed with the Myc‐Tag binary image (“AND” operation) using the Image Calculator feature. Mean gray values of the resultant p85/Myc‐Tag overlay and the individual p85 binary image were measured, and percent (%) p85 sequestration per cell was calculated as:

$$Percent\;p85\;Sequestration\;per\;Cell=\frac{p85\;\mathrm{AND}\;\mathrm{Myc}-\mathrm{Tag}}{p85}\times 100$$

Per PH‐domain construct, 15–18 cells were imaged, with 5–8 cells per biological replicate, from three biological replicates per construct. Percent (%) p85 sequestration per cell was then plotted per PH‐domain construct.

### 4.8 Generation of Protein Lysates and Western Blotting

Protein lysates from cell monolayers were generated as follows: media was collected and stored on ice, while cells were gently washed with 1xPBS. The media fraction and the PBS wash were combined and centrifuged at 1750 rpm for 5 min at 22°C to isolate weakly adherent cells; weakly adherent cell pellets were stored on ice. The remaining cell monolayer was scraped into radioimmunoprecipitation assay buffer (RIPA, Sigma, St. Louis, MO), supplemented with Halt protease and phosphatase inhibitors (Thermo Fisher, Waltham, MA), and then combined with the weakly adherent cell pellet.

Protein lysates from primary tumors were generated as follows: tumors were flash frozen in liquid nitrogen, followed by vigorous tissue homogenization using mortar and pestle. Samples were then dissolved in RIPA buffer (Sigma, St. Louis, MO), supplemented with Halt protease and phosphatase inhibitors (Thermo Fisher, Waltham, MA). All samples collected in RIPA buffer were then lysed via rotation for 30 min at 4°C, followed by centrifugation at 20 000 g for 10 min at 4°C to isolate the protein fraction from cell debris.

Protein lysate concentration was determined using the Quick Start Bradford Protein Assay (Bio‐Rad, Hercules, CA), and 15–20 µg of protein lysates were separated using either NuPAGE 3–8% Tris‐acetate SDS‐PAGE gels (specifically for Western blots of obscurin expression) or NuPAGE 4%–12% Bis‐Tris gels (all other proteins). Proteins were transferred to nitrocellulose membranes for immunoblotting. All membranes were blocked for 2 h in 3% BSA in Tris Buffered Saline (TBS) with 0.1% Tween‐20 (TBST), before overnight incubation at 4°C with primary antibodies to obscurin‐Ig58/59 (rabbit polyclonal; 0.5 µg/mL[4]), HSP90 (C45G5, rabbit monoclonal antibody, Cell Signaling Technology, Danvers, MA; #4877), Myc‐Tag (71D10, rabbit monoclonal antibody; Cell Signaling Technology, Danvers, MA; #2278), pan‐Akt (C67E7, rabbit monoclonal antibody; Cell Signaling Technology, Danvers, MA; #4691), GAPDH‐71.1 (mouse monoclonal antibody; Sigma, St. Louis, MO; G8795), PI3K/p85 (19H8, rabbit monoclonal antibody; Cell Signaling Technology, Danvers, MA; #4257), phospho‐PI3K/P85‐Tyr458/p55‐Tyr199 (rabbit monoclonal antibody; Cell Signaling Technology, Danvers, MA; #4228), phospho‐Akt‐Thr308 (C31E5E, rabbit monoclonal antibody; Cell Signaling Technology, Danvers, MA; #2965), phospho‐Akt‐Ser473 (D9E, XP rabbit monoclonal antibody; Cell Signaling Technology, Danvers, MA; #4060), PARP (46D11, Rabbit monoclonal antibody; Cell Signaling Technology, Danvers, MA; #9532); Cleaved PARP (Asp214) (D64E10, XP Rabbit monoclonal antibody; Cell Signaling Technology, Danvers, MA; #5626), MMP‐1 (E9S9N, rabbit monoclonal antibody; Cell Signaling Technology, Danvers, MA; #54376), MMP‐2 (D4M2N, rabbit monoclonal antibody; Cell Signaling Technology, Danvers, MA; #40994), and MMP‐9 (D6O3H, XP Rabbit monoclonal antibody; Cell Signaling Technology, Danvers, MA; #13667) in 3% BSA TBST overnight at 4°C. Following extensive washes with TBST, membranes were incubated with horseradish peroxidase (HRP) conjugated anti‐rabbit or anti‐mouse secondary antibody (Cell Signaling Technology, Danvers, MA). Immunoreactive bands were visualized either with Pierce ECL Western Blotting Substrate (Thermo Fisher, Waltham, MA) or SignalFire ECL Reagent (Cell Signaling Technology, Danvers, MA) kits. Densitometric evaluation was performed using ImageJ software (National Institutes of Health, Bethesda, Maryland). Three to seven biological replicates were performed for each experiment. All blots are presented in grayscale mode. Image brightness and size may have been adjusted uniformly across the entire image; please refer to raw data available on FigShare.

### 4.9 Co‐Immunoprecipitation Experiments

Control Myr‐Myc and Myr‐oPH‐Myc adenovirally‐infected MDA‐MB‐231 cells grown in 6‐well plates were washed twice with ice‐cold PBS and lysed on ice using 100 µL per well of lysis buffer (50 mM HEPES pH 7.4, 120 mM NaCl, 2 mM EDTA, 2 mM MgCl_2_, 0.5% Triton X‐100) supplemented with protease and phosphatase inhibitors (Thermo Fisher Scientific, Waltham, MA, USA). Lysates were collected, passed through a 1 mL syringe for homogenization, incubated for 30 min at 4°C, and cleared by centrifugation at 13 000 X g, for 10 min. Protein concentration was measured using the BCA Assay (Thermo Fisher Scientific, Waltham, MA, USA), and 200 µg of total protein was used for each immunoprecipitation experiment.

Lysates were first precleared with Protein G–agarose (Roche, Basel, Switzerland; #11719416001) to reduce nonspecific binding. Precleared lysates were subsequently incubated overnight at 4°C with Protein G‐agarose preloaded with 1 µg of the appropriate primary antibody. The following rabbit monoclonal antibodies were used: Myc (71D10, rabbit monoclonal antibody; Cell Signaling Technology, Danvers, MA; #2278), PI3K‐p85 (19H8, rabbit monoclonal antibody; Cell Signaling Technology, Danvers, MA; #4257), and PI3K‐p110*α* (C73F8, rabbit monoclonal antibody; Cell Signaling Technology, Danvers, MA; #4249). After incubation, samples were washed using lysis buffer containing 0.1% Triton X‐100 followed by PBS. Bound proteins were eluted in 2×SDS sample buffer (Invitrogen, Thermo Fisher Scientific, Waltham, MA, USA; #LC2676) containing 100 mM DTT and heated at 70°C for 10 min before SDS–PAGE analysis.

### 4.10 Wound Healing Assay

MDA‐MB‐231 and SKBr3 cells were plated at a 3 × 10^5^ cells/well density in 6‐well plates. MDA‐MB‐231 cells were allowed to adhere for 24 h before they were treated with Myr‐Myc control or Myr‐oPH‐Myc adenovirus (MOI 400) in complete growth media for 24 h. Cells were supplied with fresh growth media for 24 h, and then a scratch was performed. SKBr3 cells were allowed 6 days to reach confluence following plating, after which one well was trypsinized to obtain a final cell count, while the other wells were treated with Myr‐Myc control or Myr‐oPH‐Myc adenovirus (MOI 400) in complete growth media. Twenty‐four h following adenovirus treatment, cells were supplied with fresh growth media for an additional 24 h, and then a scratch was performed.

All scratches were generated with a 200 µL sterile pipette tip and cells were subsequently washed with 1X PBS. Following the scratch/wound, cells were cultured in complete growth media for 42 h (MDA‐MB‐231) and 96 h (SKBr3) in a humidified tissue culture incubator with 5% CO_2_ at 37°C. Images were taken at 0, 16, 24, and 42 h (MDA‐MB‐231) or 0, 24, 45, and 96 h (SKBr3) using the EVOS FL cell imaging system (Thermo Fisher, Waltham, MA) under a 4X objective. Collective cell migration was quantified as percent (%) wound closure, where cell edge boundaries were drawn manually and quantified using ImageJ software (National Institutes of Health, Bethesda, Maryland); five to six biological replicates were performed per cell line.

### 4.11 Transwell Migration Assay

Transiently transfected, adenovirus‐infected, and LNP‐treated MDA‐MB‐231 or SKBr3 trypsinized cells were plated at a density of 5 × 10^4^ or 1 × 10^6^ cells, respectively, in 500µL of serum‐free medium onto a 24‐well impermeable insert upper chamber containing 8.0µm sized pores (Corning, Corning, NY). The lower chamber of the wells was filled with complete growth media containing 10% FBS. Chambers were incubated for 24 h (MDA‐MB‐231) or 48 h (SKBr3) in a humidified tissue culture incubator with 5% CO_2_ at 37°C. Non‐migratory cells on the upper surface were removed using a cotton swab, while migratory cells on the lower surface were stained using a fixative and two stain solutions (Modified Giemsa) supplied by the Differential Quick Stain Kit (Polysciences, Inc., Warrington, PA) for 5 min per solution, followed by extensive washing in distilled water between solution stains. Membranes were then removed from the insert and placed on slides for imaging. Migrating cells were quantified by counting 3 random fields from three to four independent experiments under an inverted light microscope (Olympus IX51, Center Valley, PA; 10X objective).

### 4.12 Microfluidic Device Fabrication, Cell Seeding, Live‐cell Imaging, and Analysis

Adenoviral‐treated MDA‐MB‐231 cells were selected for construct expression using complete growth media supplemented with 500 µg/mL Geneticin G418 (Life Technologies) for 4–7 days, with fresh Geneticin G418 complete growth media being re‐supplied every 72 h. Of note, parental SKBr3 cells displayed minimal single cell migration through confined microchannels, as previously reported [53]; therefore, they could not be reliably tested. PDMS‐based microfluidic devices containing a parallel series of microchannels of 10 µm in height, 3 µm in width, and 200 µm in length were fabricated as previously described [54, 55]. All microchannel dimensions were routinely confirmed by laser profilometer and then incubated with rat tail collagen I (20 µg/mL, Thermo Fisher, Waltham, MA) for 1–2 h at 37°C in 5% CO_2_ prior to migration assays. Migration assays were performed in DMEM containing 10% FBS and 1% penicillin‐streptomycin supplemented with 500 µg/mL Geneticin G418. No chemotactic stimulus was provided. For each device, 20 µL of 5 × 10^6^ cells/mL cell suspension in Geneticin G418 complete growth media was added to the inlet well. Prior to migration experiments, fresh Geneticin G418‐containing growth media was added to the inlet and outlet wells. Timelapse images were subsequently recorded in 10 min intervals for up to 24 h under an inverted Nikon Eclipse Ti microscope (Nikon, Tokyo, Japan) equipped with a stage‐top incubator (Okolab, Pozzuoli, Italy, or Tokai Hit, Shizuoka, Japan) at 37°C in 5% CO_2_, automated controls (NIS‐Elements, Nikon), and 10x/0.45 numerical aperture Ph1 objective. Cell migration analysis was performed as previously described [54, 55]. Live cell migration videos were exported to ImageJ (National Institutes of Health, Bethesda, Maryland). Cell entry time and percent cell entry were calculated manually, where cell entry time was defined as the time interval from the time point the leading cell edge initiated entry into a microchannel until the entire cell had fully entered. All tracks of fully entered individual cells were obtained using the MTrackJ plugin (Biomedical Imaging Group Rotterdam, Erasmus University Medical Center, Netherlands). Cell migration speed, velocity, and persistence were subsequently calculated from these tracks using a custom MATLAB script (MathWorks, Natick MA). Experiments were repeated three independent times.

### 4.13 Adhesion Assays

96‐well plates were precoated with ECM proteins as previously described [56]. In brief, plates were incubated for 24 h in a humidified tissue culture incubator at 37°C, 5% CO2 with 6 µg/mL of either human fibronectin, collagen I, or laminin (Corning, Corning, NY), dissolved in 1X PBS. Control wells for adhesion to plastic were precoated with equal volumes of 1X PBS. Matrix‐coated wells were blocked with 3% BSA in 1X PBS for 1 h at 37°C, 5% CO2 in a humidified tissue culture incubator. Adenovirus‐treated MDA‐MB‐231 and SKBr3 cells were plated in each well at a 3 × 10^4^ cells/well density and incubated in a humidified tissue culture incubator at 37°C, 5% CO2 for 30 min and 2 h, respectively. Immediately following adhesion, wells were washed 3 times with 1X PBS, fixed with 100% methanol at ‐20°C for 15 min, and stained with DAPI. Plates were subsequently stored in the dark at 4°C and imaged within one week using the EVOS FL cell imaging system (Thermo Fisher, Waltham, MA) under a 4X objective. Adherent cells were quantified by summing DAPI positive particles present per field from three random fields per well, from four to six biological replicates using ImageJ software (National Institutes of Health, Bethesda, Maryland).

### 4.14 Filopodia Analysis

Following staining for RFP, Myc‐tag, and β‐actin, as well as image acquisition of Myr‐Myc and Myr‐oPH‐Myc adenovirally‐transduced single cells, cell filopodia were counted and their length measured using the ImageJ Single Image_FiloQuant plugin (Turku, Finland). In short, ten, adenovirus‐treated, RFP‐positive cells per construct (i.e., Myr‐Myc and Myr‐oPH‐Myc) were selected at random per biological replicate for analysis (*n* = 5 biological replicates per ECM matrix substrate). To visualize filopodia, cell images for β‐actin staining were contrast‐adjusted and cropped to include only the selected cell edges (containing filopodia), while surrounding RFP‐negative cells were cleared from the image. Processed cell images were converted to TIFF files. FiloQuant settings (edge detection, filopodia detection, and contour detection thresholds) per TIFF file were custom‐adjusted to highlight cell edge protrusions and exclude false positive hits, as outlined by the FiloQuant Manual V1.0 (https://imagej.net/media/filoquant‐manual‐v1.pdf).

### 4.15 Bradykynin Treatment

Cells were treated with 100 ng/ml bradykinin (Tocris, Bristol, UK #3004) in all assays. For imaging of filopodia via β‐actin fluorescence, 10 min prior to fixation with 100% methanol, bradykinin‐containing growth media was added to cells while incubated at 37°C, 5% CO_2_ in a humidified tissue culture incubator. For transwell migration assays, cells were plated in bradykinin‐containing serum‐free media in the upper chamber, whereas the lower chamber contained 100 ng/mL bradykinin in growth media. For adhesion assays, adenovirus‐treated cells were plated in bradykinin‐containing growth media and allowed to adhere for 30 min at 37°C, 5% CO_2_ in a humidified tissue culture incubator, before being further processed for staining and visualization.

### 4.16 Spheroid Formation and 3D Collagen Invasion Assay

Spheroid experiments were performed using either adenovirus pre‐treated or untreated MDA‐MB‐231 cells, as previously described [57]. Parental SKBr3 cells were unable to form spheroids, likely due to their weak(er) metastatic potential compared to MDA‐MB‐231 cells [58] and therefore were not tested. 3 × 10^3^ MDA‐MB‐231 cells pre‐treated with Myr‐Myc (MOI:400) or Myr‐oPH‐Myc (MOI:400) adenovirus were suspended in 150 µL of ice‐cold Growth Factor Reduced Matrigel (Corning, Corning, NY), which was generated by diluting DMEM containing 10% FBS and 1% penicillin‐streptomycin at 1:50 ratio Matrigel to DMEM media. The cell‐Matrigel suspension was then gently plated in different wells followed by incubation at 37°C, 5% CO_2_ in a cell culture incubator for 48 h before embedding for invasion assays. For spheroids treated with LNPs, 3 × 10^3^ untreated MDA‐MB‐231 cells were suspended in the same Matrigel‐DMEM media and allowed to form spheroids for 24 h, before addition of either Myr‐Myc, Myr‐oPH‐Myc, Myr‐kPH‐Myc, or Myr‐γPH‐Myc LNPs directly into each well at 20 µg/mL. LNP‐treated spheroids were then allowed to grow for an additional 24 h before embedding for invasion assays.

3D collagen invasion assays using spheroids were performed as previously described^7^. In short, 3 mL of rat tail collagen type I (Corning, Corning, NY) were mixed with 375 µl of 10x DMEM‐low glucose (Sigma, St. Louis, MO). The pH of the mixture was gradually adjusted to physiological levels with NaOH. 30 µl of the mixture were then added to a Falcon 24 well‐plate (Corning, Corning, NY) and incubated at 37°C, 5% CO_2_ in a cell culture incubator for ∼20 min. Spheroids were collected into 1.5 mL Eppendorf tubes by disrupting the Matrigel gently and incubated on ice for >10 min to further depolymerize the Matrigel. Spheroids were isolated by centrifugation (1500 rpm) for 6 sec and resuspended into 240 µl of the pH‐balanced collagen mixture. Next, 120 µl of the spheroid‐collagen mixture were plated in each well and incubated at 37°C, 5% CO_2_ in a cell culture incubator for 1 h. Following collagen polymerization, 1 mL cell culture media was added to each well. Time‐lapse images were recorded in 20 min intervals for 16–48 h under an inverted Nikon Eclipse Ti microscope (Nikon) equipped with a stage‐top incubator (Okolab or Tokai Hit) at 37°C and 5% CO2, automated controls (NIS‐Elements, Nikon) and a 10x/0.45 numerical aperture Ph1 objective. Cell tracks were obtained using the MTrackJ plugin (Biomedical Imaging Group Rotterdam, Erasmus University Medical Center, Netherlands), and resultant cell velocity, speed, and mean square displacement (MSD) were calculated using a custom‐made MATLAB script. Spheroid area size and normalized area of expansion were measured manually using ImageJ software (National Institutes of Health, Bethesda, Maryland). Data was acquired from three independent experiments.

### 4.17 Gelatin Invasion Assay, Invadopodia Quantification, and MMP Staining

96‐well plates (Ibidi, Fitchburg, Wisconsin) were coated with Fluorescein‐Gelatin using the QCM Gelatin Invadopodia Assay Kit (Green) (Millipore Sigma, Burlington, MA; ECM670). Following aldehyde quenching, MDA‐MB‐231 and SKBr3 cells treated with Myr‐Myc (MOI:400) or Myr‐oPH‐Myc (MOI:400) adenovirus were seeded at a 3 × 10^4^ cells/well density onto fresh Fluorescein‐Gelatin‐coated plates and allowed to invade for 24 h (MDA‐MB‐231) or 72 h (SKBr3). Cells were subsequently fixed in 3.7% paraformaldehyde (PFA), 1X D‐PBS solution for 30 min in the dark, washed with 1X D‐PBS, and stored in the dark at 4°C for no longer than one week.

To preserve fluorescence, all further steps were performed in the dark. To stain invadopodia and MMPs, fixed cells were first permeabilized with 0.3% Triton‐X‐100 for 5 min at room temperature, blocked with 3% BSA for 60 min at room temperature, and then incubated with primary antibodies against Myc‐Tag (mouse monoclonal antibody 9E10; Abcam, Waltham, MA; ab32), MMP‐2 (rabbit monoclonal antibody D4M2N; Cell Signaling Technology, Danvers, MA; #40994), or MMP‐9 (XP rabbit monoclonal ab D6O3H; Cell Signaling Technology, Danvers, MA; #13667) in 2% BSA, 0.25% Triton‐X‐100 overnight at 4°C. Cells were washed with 1X D‐PBS and incubated with the appropriate secondary antibodies, anti‐mouse AlexaFluor 405 (Thermo Fisher, Waltham, MA) and anti‐rabbit AlexaFluor 594 (Thermo Fisher, Waltham, MA), and counterstained with AlexaFluor 647 Phalloidin (Cell Signaling Technology, Danvers, MA; #8940) in 2% BSA, 0.25% Triton‐X‐100 for 1 h at room temperature. Cells were subsequently washed 2x with 2% BSA, 0.25% Triton‐X‐100 and 3x with 1X D‐PBS, before they were immersed in Ibidi Immersion Oil (Ibidi, Fitchburg, Wisconsin) and stored at 4°C in the dark.

All images were acquired using a Nikon W1 spinning disk with a 60x oil immersion objective, with pixel sizes at 0.11 µm/pixel. For image presentation, channels were pseudo‐colored using ImageJ software (National Institutes of Health, Bethesda, Maryland) as follows: gelatin (red), RFP (gold), Myc‐tag (Cyan), Phalloidin/F‐actin (green), and MMP2/9 (magenta). To visualize gelatin degradation and invadopodia, the imaging plane was centered at the cell‐gelatin interface. To acquire images of MMP expression, the imaging plane was centered above the gelatin plane at the cell body using phalloidin/F‐actin staining as reference. Gelatin degradation was quantified as 1/Mean Gray Value of fluorescein fluorescence from 10 randomly selected, RFP‐positive cells per biological replica for each construct (*n* = 3 biological replicates, 30 cells per construct) using ImageJ software (National Institutes of Health, Bethesda, Maryland). Fluoresceine gelatin and Phalloidin/F‐actin channels were input into the ImageJ JaCoP plugin (National Institutes of Health, Bethesda, Maryland) and invadopodia were quantified as 1‐Mander's Coefficient for the fraction of phalloidin/F‐actin signal that overlaps with gelatin signal. MMP expression was quantified from 20 randomly selected, RFP‐positive cells per biological replica for each construct (*n* = 3 biological replicates, 60 cells per construct), as the mean gray value of MMP fluorescence per cell, using ImageJ software (National Institutes of Health, Bethesda, Maryland).

### 4.18 Animal Studies

All animal studies were performed following the Institutional Animal Care and Use Committee (IACUC) procedures and guidelines of the University of Maryland, Baltimore under an approved protocol. Eight‐to‐twelve‐week‐old female NOD SCID mice weighing 19–25 g was obtained from Charles River (Fredrick, MD) and fed ad libitum. Prior to injection, animals were randomly assigned to the Myr‐Myc or Myr‐oPH‐Myc LNP group. At Day 0, 1 × 10^6^ MDA‐MB‐231 cells were suspended in 100 µL PBS and mixed with equal volume of Matrigel (Corning). Cell number was quantified via Countess Automated Cell Counter (ThermoFisher). The cell suspension was injected subcutaneously into the fourth mammary gland on the ventral surface of the abdomen of each female mouse. Following tumor implantation, 10 µg Myr‐Myc or Myr‐oPH‐Myc LNPs were injected into primary tumors that had grown to ∼100 mm^3^ or into day‐7 tumor‐bearing mouse mammary fat pads, once a week for 6 weeks. Tumor volumes were measured with an external digital caliper (Thomas Scientific, Inc.) weekly until endpoint, along the two longest perpendicular axes in the x/y plane to the nearest 0.1 mm. Depth was assumed to be equivalent to the shortest of the perpendicular axes (y), and volume was calculated according to the: V  =  xy^2^/2 formula, as the standard practice for xenograft tumors.

### 4.19 XTT Cell Viability Assays

MDA‐MB‐231 cells were seeded at a density of 3 × 10^4^ cells/well in 96‐well clear‐bottom black plates (Corning, Lowell, MA, USA) and subsequently treated with either Myr‐Myc or Myr‐oPH‐Myc adenovirus, or with 20 µg/mL Myr‐Myc or Myr‐oPH‐Myc LNPs. After 48 h of growth in DMEM supplemented with 10% FBS and 1% penicillin‐streptomycin, cell viability was measured with the CyQUANT XTT Cell Viability Assay (ThermoFishcer), according to the manufacturer's instructions. Briefly, at endpoint cells were incubated with XTT reagent for 5 h in a 37°C 5% CO_2_ humidified tissue culture incubator and absorbance was measured at 450 nm using a microplate reader; 6 or 10 independent experiments were performed for adenovirus‐ or LNP‐treated cells, respectively.

### 4.20 Tumorsphere Formation Assays

Single MDA‐MB‐231 cells transduced with either Myr‐Myc or Myr‐oPH‐Myc adenovirus were plated in ultralow attachment plates (Corning, Lowell, MA, USA) at a density of 10 000 viable cells/ml in MammoCult complete media consisting of MammoCult Basal Medium (Human) supplemented with MammoCult Proliferation Supplement (Human). Tumorsphere cultures were supplemented 1:1 with fresh MammoCult Complete Media after 3 days and maintained for a total of 7 days, at which point spheres were imaged using the EVOS FL cell imaging system (Thermo Fisher, Waltham, MA) under a 4X objective. Sphere diameters were measured using ImageJ software (National Institutes of Health, Bethesda, Maryland) and those greater than ∼100 µm were counted as tumorspheres. The number of tumorspheres per well was quantified, and tumorsphere diameters per well were averaged. Experiments were performed six independent times.

### 4.21 *hLINE* qPCR Analysis of Metastatic Burden

DNA was extracted from frozen lung, axillary lymph node, liver, bone, and brain specimens using the DNeasy Blood and Tissue kit (Qiagen), according to the manufacturer's recommendations. For bone, surrounding muscle tissue was dissected followed by flash‐freezing of the entire femur in liquid nitrogen, and vigorous homogenization using mortar and pestle until all bone was reduced to powder. For lung, axillary lymph node, liver, and brain, 10–15 mg per piece was collected from each harvested organ, weighed, and recorded. Samples were lysed overnight at 56°C, and purification steps were followed as outlined in the manufacturer's protocol. DNA was eluted in 100 µl (lungs and liver) or 50 µl (axillary lymph node, bone, and brain) of DNA‐RNA free water. Quantification of *hLINE* levels, which serve as proxy for the amount of human DNA present in mouse organs, was performed with qPCR, as previously reported[4], in a 20 µl reaction with the following components: 10 µl iTaq Universal SYBR Green Supermix (Bio‐Rad), 1.5 µl of each 10 µM forward and reverse primers, 4.5 µl purified DNA and 2.5 µl water. In each qPCR experiment, serial dilutions of human DNA extracted from MDA‐MB‐231 cells using the DNeasy Blood and Tissue kit (Qiagen) were included to serve as standards. Results are shown as the amount of human DNA per mg of digested tissue sample. Tissues from nine animals per group were analyzed and three technical qPCR replicates were performed for each biological replicate; the *hLINE* qPCR primer sequences are provided in Table S4.

### 4.22 Primary Tumor qPCR Analysis of LNP‐Delivered Myr‐Myc and Myr‐oPH‐Myc pDNA

DNA was extracted from frozen primary tumor specimens using the DNeasy Blood and Tissue kit (Qiagen) according to the manufacturer's recommendations. For each tumor, 10–15 mg per piece was collected, weighed, and recorded. Samples were lysed overnight at 56°C, and purification steps were followed as outlined in the manufacturer's protocol. DNA was eluted in 100 µl of DNA‐RNA free water. Quantification of Myr‐Myc or Myr‐oPH‐Myc pDNA levels, which serve as confirmation of successful LNP delivery of each pDNA construct, was performed with qPCR as previously reported [4]. Briefly, qPCR was performed in a 20 µl reaction with the following components: 10 µl iTaq Universal SYBR Green Supermix (Bio‐Rad), 1.5 µl of each 10 µM forward and reverse primers, 4.5 µl purified DNA and 2.5 µl water. In each qPCR experiment, serial dilutions of Myr‐Myc or Myr‐oPH‐Myc plasmid DNA extracted from Myr‐Myc or Myr‐oPH‐Myc expressing MDA‐MB‐231 cells using the DNeasy Blood and Tissue kit (Qiagen) were included to generate standard curves of each pDNA construct. Results are shown as the amount of Myr‐Myc or Myr‐oPH‐Myc pDNA per mg of digested primary tumor sample. Tumors from nine animals per group were analyzed and three technical qPCR replicates were performed for each biological replicate; the Myr‐Myc and Myr‐oPH‐Myc qPCR primer sequences are provided in Table S4.

### 4.23 Primary Tumor RNA Extraction and RT‐qPCR

Total RNA was extracted from primary tumors using the Qiagen RNeasy Plus Mini Kit with gDNA eliminator columns (Qiagen, Germantown, MD), which selectively and efficiently remove genomic DNA. Tumors were flash frozen in liquid nitrogen, followed by vigorous homogenization using mortar and pestle until all tissue was reduced to powder and subsequently processed for RNA extraction. cDNA was synthesized from ∼700 ng of total RNA using the SuperScript III First‐Strand Synthesis System (Invitrogen, Thermo Fisher). KiCqStart Universal SYBR Green qPCR ReadyMix (Sigma, St. Louis, MO) was used for qPCR reactions. RNA expression levels were normalized to GAPDH using the DDCt method. Three technical qPCR replicates for each of the nine primary tumors were performed; the Myr‐Myc and Myr‐oPH‐Myc qPCR primer sequences are provided in Table S4.

### 4.24 Immunohistochemistry and Pathology

Animals that reached the designated endpoint (i.e., tumors that received up to 6 injections of either Myr‐Myc or Myr‐oPH‐Myc LNPs) were sacrificed. The lungs, liver, and bone were removed, fixed in formalin for 24 h, embedded in paraffin wax, and serially sectioned (4 µm thick). All immunohistochemistry Human Mitochondria and H&E staining was performed by HistoWiz (Histowiz.com; Brooklyn, NY). A pathology report was performed by a Histowiz senior board‐certified research pathologist, where whole slide images, and six regions of interest per slide, were blindly reviewed in their entirety, with all samples designated as experimental. The complete pathology report is available as a Supplemental Data file.

### 4.25 Alpha‐Fold, In Silico Mutagenesis, and Molecular Dynamics Simulations

All models were generated using alpha‐fold [59] and assessed for quality using per‐residue local difference distance test (pLDDT) and root mean square deviation (RMSD) across multiple models. Models were equilibrated for >50 ns in explicit solvent at 37°C, 150 mM NaCl using the Amber14 force field in the program YASARA [60]. All mutations were generated from the equilibrated models by using the ‘swap’ command and then further equilibrated for 10 ns. Simulations were surveyed every 100 psec and analyzed using standard YASARA macros as previously described [60]. All data were visualized in PyMOL (The PyMOL Molecular Graphics System, Version 2.5.5 Schrödinger, LLC.).

### 4.26 FoldSeek Analysis

Alpha‐fold predicted structures of the obscurin, kalirin, and PLCγ1 PH‐domains complexed with the PI3K p85‐SH3 domain were input into FoldSeek [35]. Structural homologs were identified using the 3Di/AA mode and Homo sapiens (human) taxonomic filter. Homolog hits for the obscurin, kalirin, and PLCγ1 PH‐domains were conservatively defined as UniProt‐ or NIH‐annotated PH domains excluding non‐cDNA targets and registering as Bit Score>100 and E‐value<1E‐02, restricted to the AFDB50 database clusters. Each set of structurally homologous PH‐domains was then cross‐referenced with the homolog lists of the other two PH‐domains, and shared structural homologs were recorded and counted.

### 4.27 Quantification and Statistical Analysis

All data are presented as the mean ± SD or SEM from three or more independent experiments. All images are included as representative examples of the quantitative average per treatment group. Gaussian or non‐gaussian distributions were confirmed using the Kolmogorov‐Smirnov test for normal distributions. Data sets with Gaussian distributions were compared using two‐tailed Student's t‐test or one‐way ANOVA followed by Tukey's, Dunnett's, or Fisher's LSD multiple comparisons test, as appropriate. For non‐Gaussian distributions, the nonparametric Mann‐Whitney or Kruskal‐Wallis test was used for comparisons between two or more groups, respectively. Two‐way ANOVA followed by Dunn's or Sidak multiple comparisons test was used for comparisons between multiple groups with two independent variables. Statistical significance was defined as *p*<0.05. Calculations were performed using GraphPad Prism 9 or 10 (GraphPad Software). Specific details of statistical analysis per experiment are included in the figure legends.

## Author Contributions

ME: performed conceptualization, data curation, formal analysis, funding acquisition, investigation, methodology, project administration, supervision, validation, visualization, writing – original draft and writing – review and editing. AS: data curation, formal analysis, investigation, methodology, validation, writing – original draft and writing – review and editing. SC: data curation, formal analysis, investigation, methodology, visualization, writing – review and editing. SJL: formal analysis, investigation, methodology, validation, writing – review and editing. KT: formal analysis, investigation, methodology, supervision, writing – review and editing. MMM: methodology, writing – review and editing. PD: investigation, methodology, validation, writing – review and editing. TG: methodology, writing – review and editing. AK: methodology, resources, supervision, writing – review and editing. SSM: funding acquisition, supervision, writing – review and editing. NW: conceptualization, data curation, formal analysis, funding acquisition, investigation, methodology, software, project administration, resources, supervision, validation, visualization, writing – review and editing. KK: conceptualization, funding acquisition, project administration, resources, supervision, writing – review and editing. AKK: conceptualization, funding acquisition, project administration, supervision, writing – original draft and writing – review and editing.

## Funding

This work was supported by NIH/NCI R01 CA183804 (KK and AKK), NIH/NCI R01CA254193 (KK), NIH/NIGMS R35GM156305 (KK), NIH/NCI R01 CA124704 (SM), NIH/NCI Training Program in Cancer Biology T32 CA154274 (ME), NIH/NCI 5F30CA278384 (ME), NIH/NIGMS R15 GM148809 (NW), NSF MCB 2024182 (NW), and the METAvivor Foundation (SM).

## Ethics Statement

All animal studies were performed according to the Institutional Animal Care and Use Committee (IACUC) guidelines of the University of Maryland, Baltimore under the following approved protocols: AUP‐0222003 and AUP‐00002855.

## Conflicts of Interest

The authors declare that they have no conflict‐of‐interest disclosures. A Non‐Provisional Patent Application (#:19/454,051), titled: “Compositions and Methods for Treating Cancer Metastasis by Administering Nucleic Acids Encoding Pleckstrin Homology Domains” is pending.

## Supporting information




**Supporting File 1**: advs74936‐sup‐0001‐SuppMat.pdf.

**Supporting File 2**: advs74936‐sup‐0002‐TableS1‐S4.zip.

**Supporting File 3**: advs74936‐sup‐0003‐VideoS1‐S4.zip.

## Data Availability

The main data supporting the results of this study are available within the article and its Supplementary Data files, while all raw data are available in Figshare under the following link: https://figshare.com/s/471195da640504d9fec0.
