# Correction to: “Delivery of Pleckstrin‐Homology Domains Suppresses PI3K/Akt Signaling and Breast Cancer Metastasis”

**DOI:** 10.1002/advs.77498

**Published:** 2026-09-29

M. Eason, A. Sen, S. Cho, et al. “ Delivery of Pleckstrin‐Homology Domains Suppresses PI3K/Akt Signaling and Breast Cancer Metastasis.” Advanced Science 13, no. 30 (2026): e18339, https://doi.org/10.1002/advs.202518339.

After the publication of the above article, the authors identified the following inadvertent errors, which were introduced during the manual copy‐and‐paste process used to compile the raw data master Excel files.

Figure 1B The normalized densitometric values for total PI3K p85 relative to the housekeeping protein GAPDH were inadvertently duplicated and used for total Akt in replicates 2 and 3 in the *“Rep 2 & Rep 3”* worksheet of the “*Densitometry Raw Values & Analysis.xlsx”* file located in the *“Panel 1B”* folder deposited in FigShare. These errors have been corrected in the Excel file, with the revised values highlighted in yellow, and in the corresponding “*Prism Statistical Analysis.pzfx”* file. The corrected Excel and Prism files have been labeled as “Revised”.

This correction does not affect the statistical outcomes or the conclusions of Figure 1B. Specifically, pAkt T308 remains unchanged and highly significant (****p < 0.0001), pAkt S473 increases in significance from **p < 0.01 to ***p < 0.001, and the total AKT remains unaltered. The pAkt T308, pAkt S473, and total Akt graphs for Figure 1B have been recreated and are shown in comparison to the original ones.


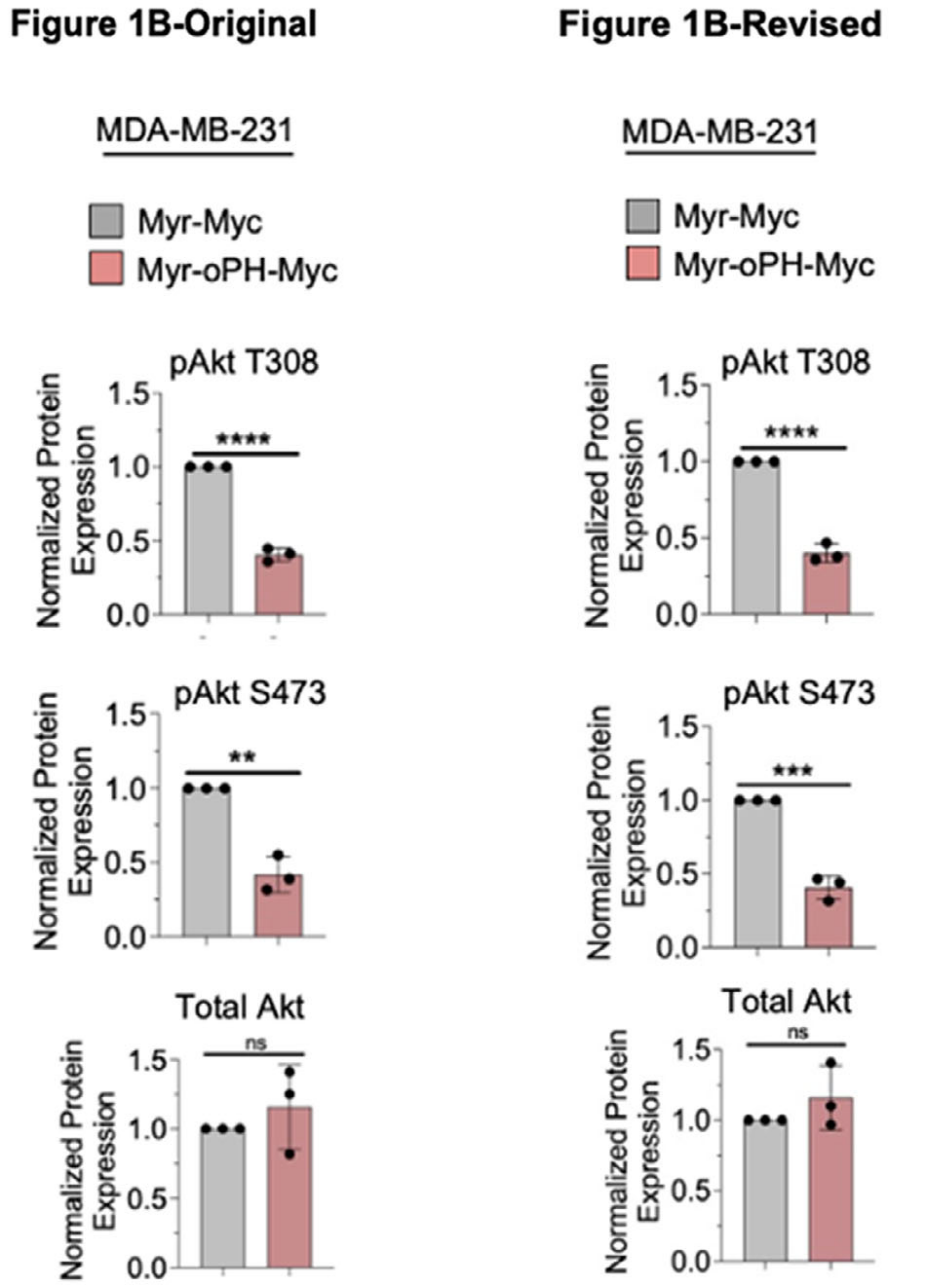


Figure 2D‐F: The measurements in rows 21–24 of the *“Myr‐oPH‐Myc(3)”* columns in the “*Rep. 2&3, Sp, Vel, Persistence*” worksheet of the *“Raw Data & Analysis.xlsx*” file located in the *“Panel 2B‐F”* folder deposited in FigShare were inadvertently duplicated from rows 17–20. This error has been corrected in the Excel file, with the corresponding cells left empty and highlighted in yellow, and in the associated *“Prism Statistical Analysis.pzfx”* file. The corrected Excel and Prism files have been labeled as “Revised”.

This correction does not affect the statistical outcomes for Figure 2D‐F, which remain unchanged, or the conclusions of Figure 2. The graphs for Figure 2D‐F have been recreated and are shown in comparison to the original ones.


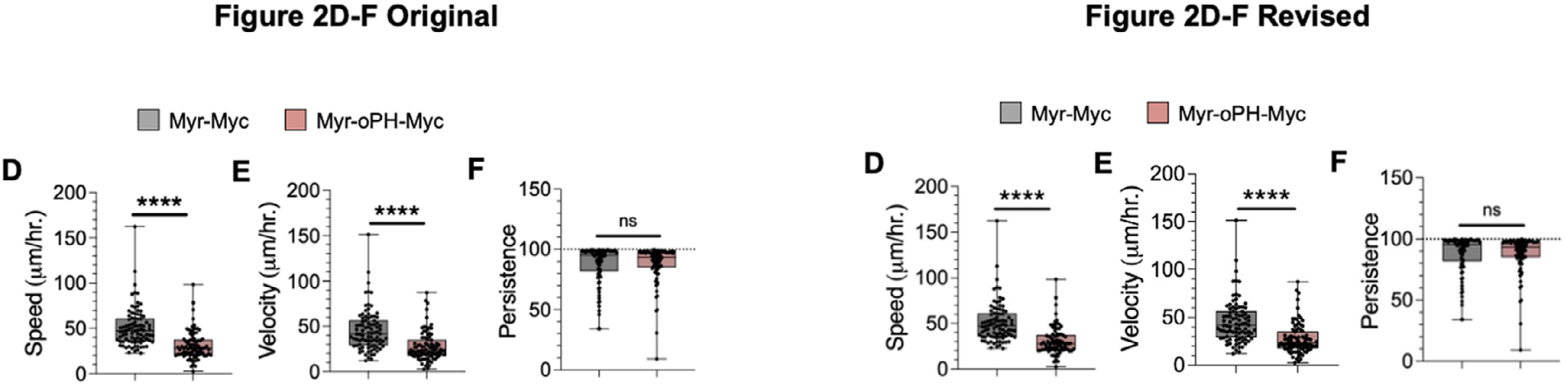


Figure 8F‐G The measurements in rows 10–12 of the *“Kalirin”* columns in the *“Raw Data”* worksheet of the *“Speed & Velocity Rep 2 Raw Data & Analysis.xlsx”* file located in both *“Panel 8F”* (speed) and *“Panel 8G”* (velocity) folders deposited in FigShare were inadvertently duplicated from rows 7–9. For ease of access by the readership, the Excel file was deposited in both folders, as the speed and velocity values are calculated from cell tracking data consisting of the x‐ and y‐coordinates and corresponding time points. This error has been corrected in the Excel file, with the corresponding cells left empty and highlighted in yellow. The corrected Excel file has been labeled as “Revised”.

The graphs for Figure 8F,G were generated using a randomized selection of data points. During this randomization process, the duplicated measurements were not selected, as documented in the *“Data Following Randomized (19)”* worksheet of the same Excel file. As such, panels F and G do not require revision.

In summary, these inadvertent errors do not affect the results of the relevant panels and figures, nor do they alter the conclusions of the study as a whole. The authors sincerely apologize for this oversight.

