# Supplementary material for: Delivery of Pleckstrin‐Homology Domains Suppresses PI3K/Akt Signaling and Breast Cancer Metastasis
Source: Adv Sci (Weinh). 2026 Mar 30;13(30):e18339. doi: 10.1002/advs.202518339 (PMC13248768; doi:10.1002/advs.202518339)
Supplement: Supplementary file 1 — Supporting File 1: advs74936‐sup‐0001‐SuppMat.pdf. [file ADVS-13-e18339-s002.pdf]

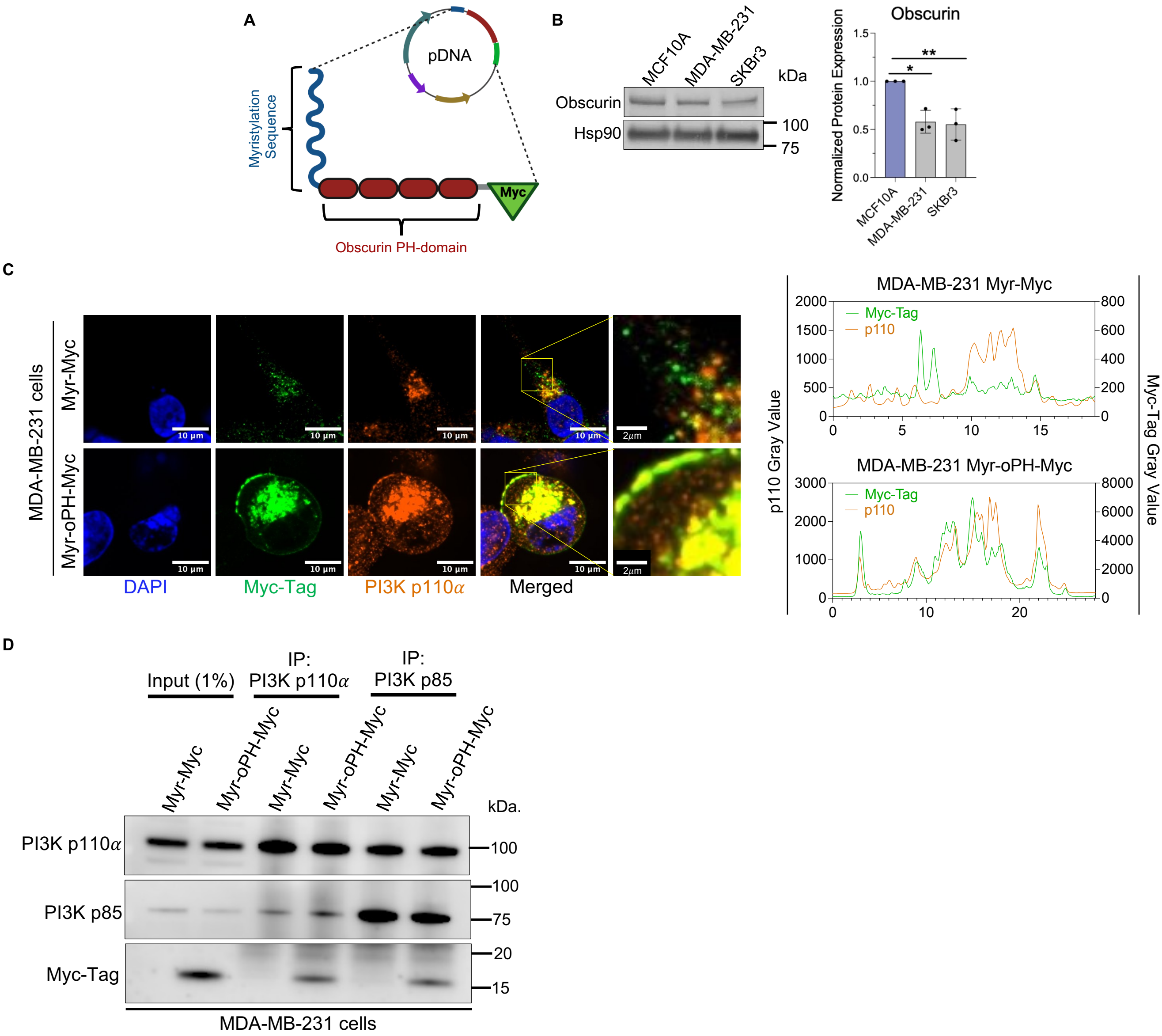

Supplementary Fig. 1

Figure S1: PI3K-p110α remains in complex with obscurin-PH-sequestered p85. (A) Schematic of myristoylated obscurin pleckstrin homology (PH)-domain with C-terminal Myc-Tag (Myr-oPH-Myc); created with Biorender.com (agreement number LL289RLO7B). **(B)** Representative Western blot image of MCF10A, MDA-MB-231, and SKBR3 cells probed for obscurin expression; Hsp90 was used as loading control. Densitometry values are presented as expression relative to MCF10A, which was set to "1" (n=3 independent experiments); One-Way ANOVA with Tukey's multiple comparison test. **(C)** Representative confocal images of adenovirally-transduced MDA-MB-231 cells expressing Myr-Myc or Myr-oPH-Myc; DAPI (blue), Myc-tag (green), PI3K-p110α (orange), and merged Myc-tag/PI3K-p110α (yellow). Cells of each treatment group vary significantly in size; scale bars differ to visualize the entire cell. Line composite graphs display Myc-tag and PI3K-p110α gray values (pixel intensity). **(D)** Representative immunoblots for PI3K p110α, PI3K p85, and Myc-Tag in immunoprecipitated fractions derived from lysates of MDA-MB-231 cells expressing the Myr-Myc and Myr-oPH-Myc constructs, indicating the 1% of total input fraction, the PI3K p110α pull-down fraction, and the PI3K p85 pull-down fraction. All data are plotted as mean ± SD; *$p<0.05$; **$p<0.01$.

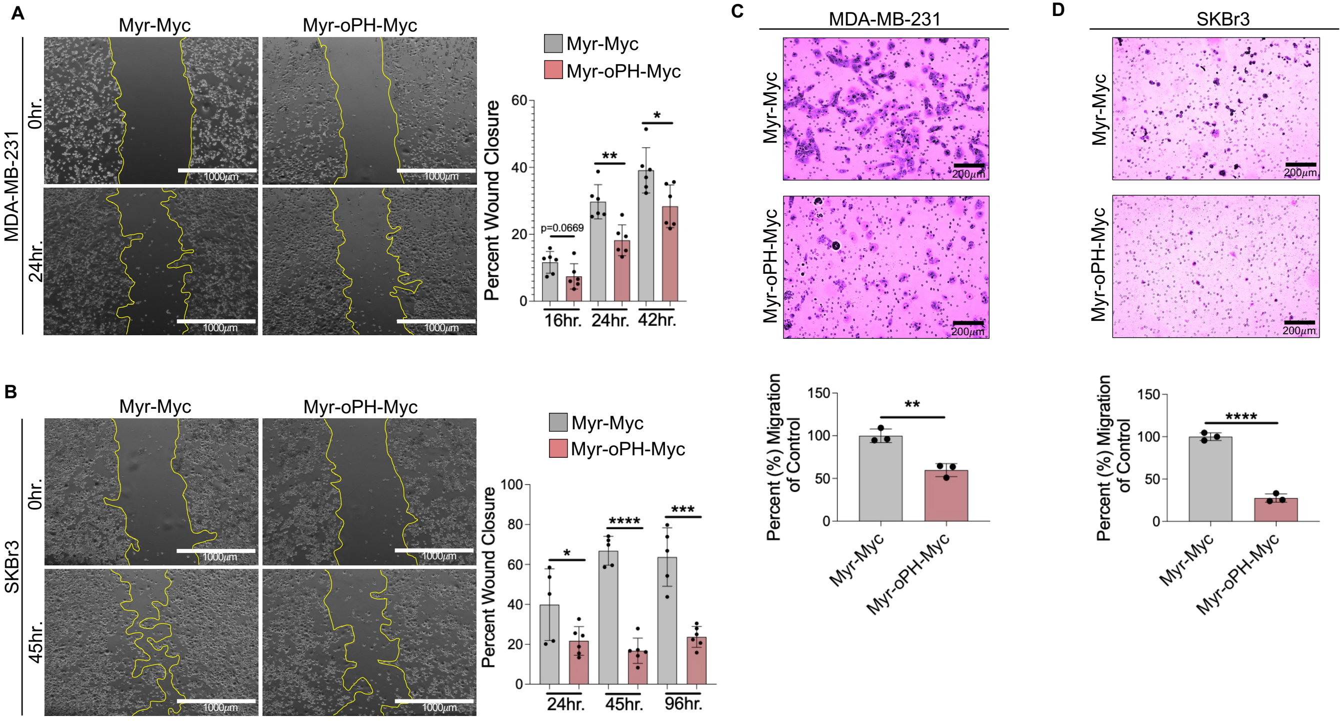

Supplementary Fig. 2

Figure S2: The obscurin PH-domain hinders breast cancer collective and chemotactic cell migration. (A-B) Representative phase contrast images of wound healing assays of adenovirus-transduced MDA-MB-231 **(A)** and SKBr3 **(B)** cells expressing the Myr-Myc and Myr-oPH-Myc constructs at 0 h and 24 h (MDA-MB-231) or 45 h (SKBr3) following scratch. Wound boarders are outlined in yellow. Percent (%) wound closure is plotted for the 16, 24, and 42 h time point (MDA-MB-231) or 24, 45, and 96 h time points (SKBr3) following scratch (n=5-6 independent experiments); two-tailed t-test. **(C-D)** Representative brightfield images of transwell migration assays of adenovirus-transduced MDA-MB-231 **(C)** and SKBr3 **(D)** cells expressing the Myr-Myc and Myr-oPH-Myc constructs. The percent (%) migrated cells per field is plotted, per treatment group (n=3 independent experiments); two-tailed t-test. All data are plotted as mean ± SD; $^*p<0.05$; $^{**}p<0.01$; $^{***}p<0.001$; $^{****}p<0.0001$.

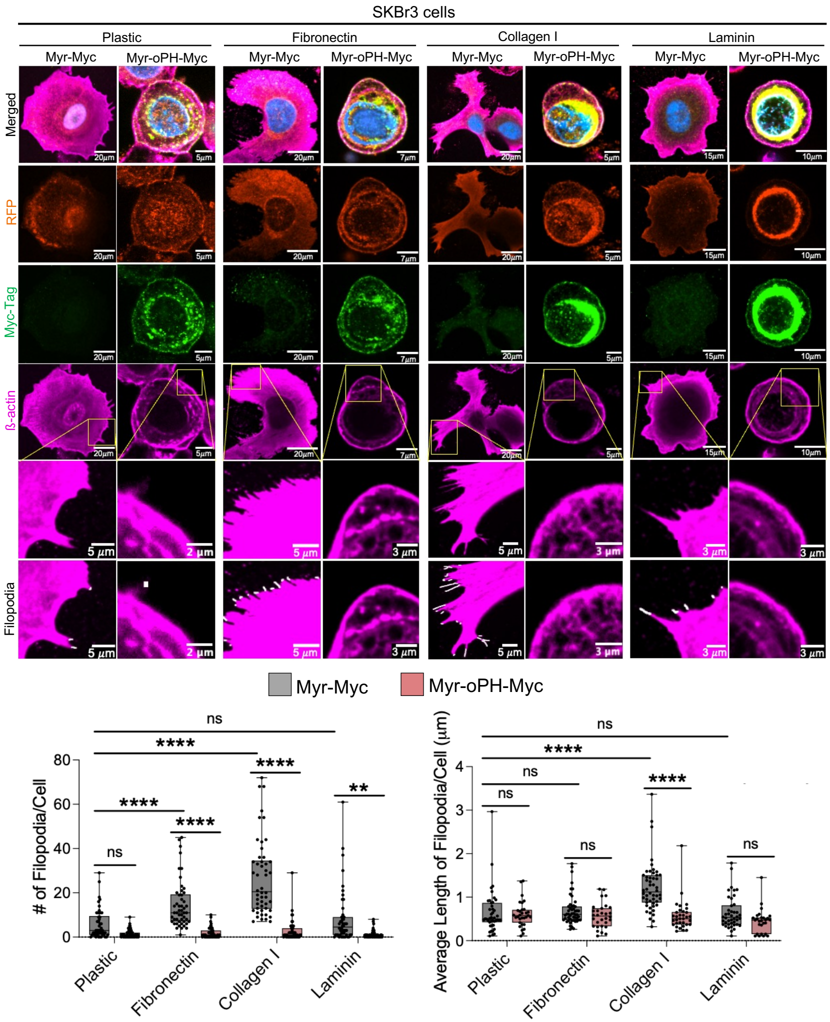

Supplementary Fig. 3

Figure S3: The obscurin PH-domain ablates HER2+ breast cancer cell filopodia. Representative confocal images of adenovirus-transduced, RFP-positive SKBr3 cells expressing the Myr-Myc or Myr-oPH-Myc construct plated on plastic, fibronectin, collagen I, or laminin substrates; RFP (orange), Myc-tag (green), β-actin (magenta), DAPI (blue), and filopodia FiloQuant Overlay mask (white). Cells and filopodia of each treatment group vary significantly in size; scale bars differ to visualize the entire cell and their filopodia. Average number and length (μm) of filopodia (FiloQuant) are plotted per cell, per extracellular matrix substrate (n=50 cells per condition pooled from 5 independent experiments; 10 cells per experiment). Statistical analysis was performed with two-way ANOVA followed by Tukey's multiple comparison test. All data are plotted as mean ± SD; **p<0.01; ****p<0.0001.

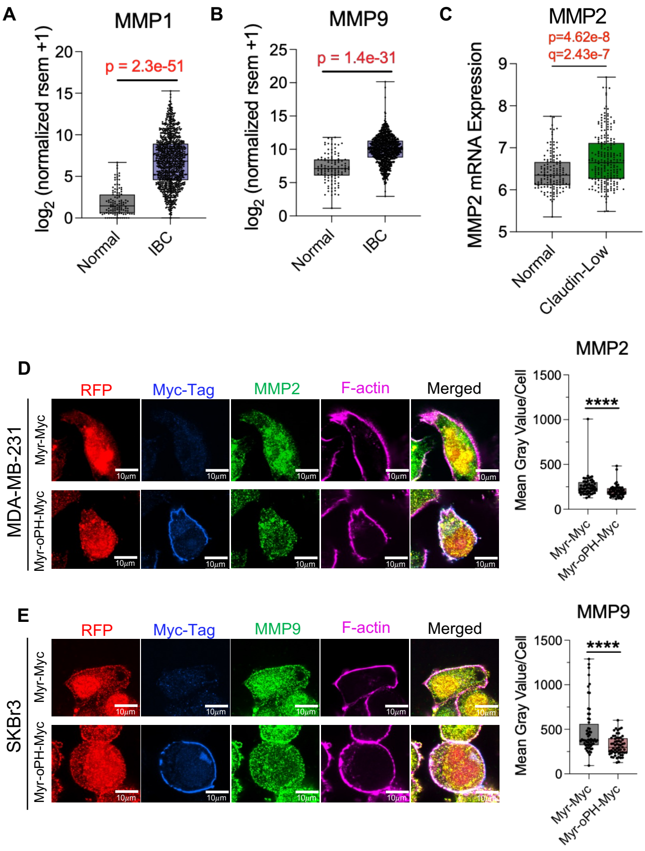

Supplementary Fig. 4

Figure S4: The Obscurin PH-domain silences matrix metalloproteinase (MMPs) expression. (A-B) Illumina HiSeq MMP1 **(A)** and MMP9 **(B)** RNAseq expression analysis (http://maplab.imppc.org/wanderer/) of normal breast (n=113 patient samples) and invasive breast carcinoma (n=1052 patient samples); Wilcoxon Rank Sum test. **(C)** cBioPortal Illumina HT-12 v3 microarray MMP2 Expression (Breast Cancer METABRIC, Nature 2012 & Nat Commun 2016) for breast carcinoma, partitioned by the PAM50 subtype classification as either normal (n=148 patient samples) or claudin-low (n=218 patient samples) breast cancer; p-values by two-tailed t-test; q-values by Benjamini-Hochberg procedure **(D-E)** Representative confocal images of adenovirus-transduced, RFP-positive MDA-MB-231 **(D)** and SKBr3 **(E)** cells expressing the Myr-Myc or Myr-oPH-Myc construct plated on gelatin; RFP (red), Myc-tag (blue), MMP2 **(D)** or MMP9 **(E)** (green), and β-actin (magenta). MMP2 **(D)** or MMP9 **(E)** mean gray values (pixel intensity) are plotted per cell (n=60 cells per construct pooled from 3 independent experiments); Mann-Whitney test. All data are plotted as mean ± SD; ****p<0.0001.

A

Lipid Nanoparticle (LNP) Sample Group	Z-avg (nm)			Polydispersity Index (PDI)			Zeta-potential (mV)		
Myr-Myc LNPs	94.15	93.78	93.12	0.09	0.10	0.09	-5.14	-5.74	-6.54
Myr-oPH-Myc LNPs	93.07	92.33	92.92	0.08	0.09	0.05	-5.63	-6.61	-6.06
Myr-kPH-Myc LNPs	95.41	94.21	94.25	0.08	0.08	0.08	-6.14	-6.26	-5.97
Myr-γPH-Myc LNPs	94.59	94.15	93.89	0.09	0.09	0.09	-5.89	-5.97	-6.01

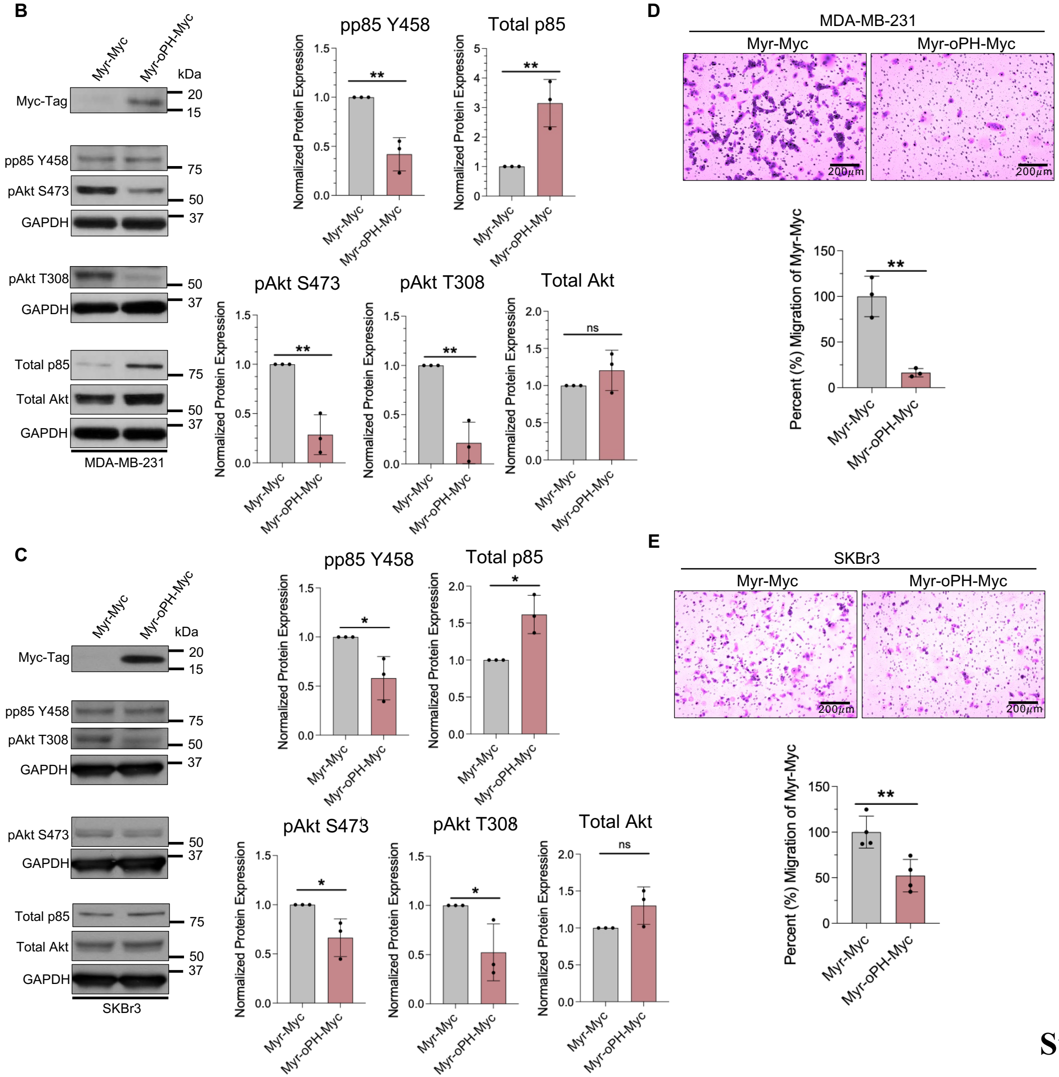

Supplementary Fig. 5

Figure S5: Lipid nanoparticle delivery of the obscurin PH-domain suppresses PI3K/Akt activation and reduces migration. (A) Size (nm), polydispersity index (PDI), and zeta-potential (mV) values of formulations for Myr-Myc, Myr-oPH-Myc, Myr-kPH-Myc, and Myr-γPH-Myc lipid nanoparticle (LNP)/pDNA mixtures. **(B-C)** Immunoblots of MDA-MB-231 **(B)** and SKBr3 **(C)** cells probed for PI3K-p85 Y458 and pAkt T308/S473 phosphorylation levels treated with LNP-delivered Myr-Myc or Myr-oPH-Myc constructs. Densitometry values are presented as expression relative to Myr-Myc control, set at "1" (n=3 independent experiments); two-tailed t-test. **(D-E)** Representative brightfield images of transwell migration assays of MDA-MB-231 **(D)** and SKBr3 **(E)** cells treated with LNP-delivered Myr-Myc or Myr-oPH-Myc constructs. The percent (%) migrated cells per field is plotted, per treatment group (n=3-4 independent experiments); two-tailed t-test. All data are plotted as mean ± SD; *$p<0.05$; **$p<0.01$.

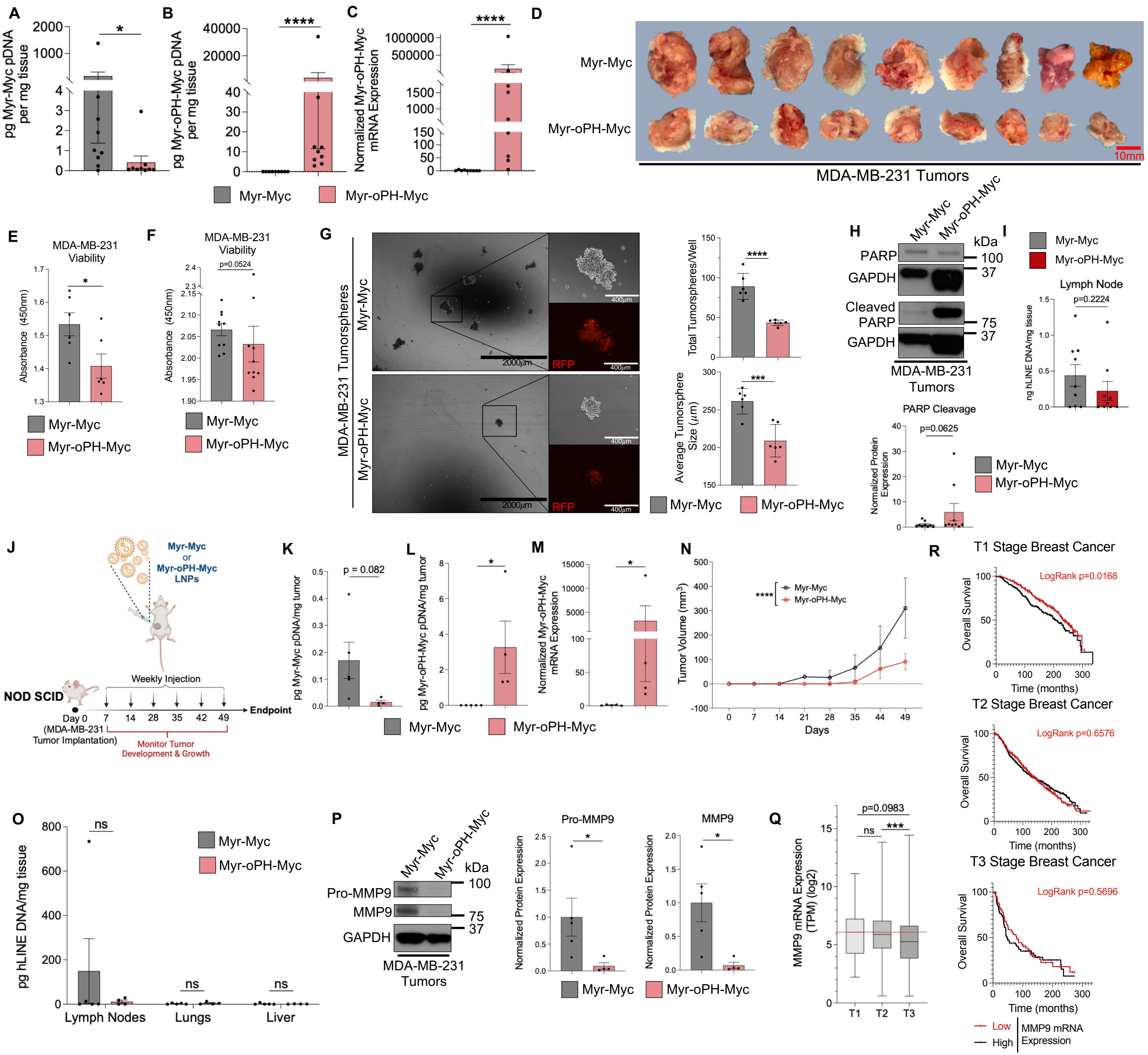

Supplementary Fig. 6

Figure S6: Lipid Nanoparticle Delivery of the obscurin PH-domain inhibits MMP expression and promotes apoptosis in breast cancer tumors. (A-B) qPCR of Myr-Myc **(A)** or Myr-oPH-Myc **(B)** pDNA constructs, plotted as pg pDNA per mg of digested tissue section from LNP-delivered Myr-Myc or Myr-oPH-Myc treated tumors (n=9 mice per group pooled from 2 independent experiments; 4-5 mice per experiment); Mann-Whitney test. **(C)** RT-qPCR for Myr-oPH-Myc mRNA expression, normalized to GAPDH, for Myr-Myc or Myr-oPH-Myc expressing tumors (n=9 mice per group pooled from 2 independent experiments; 4-5 mice per experiment); Mann-Whitney test. **(D)** Excised Myr-Myc and Myr-oPH-Myc LNP-treated primary tumors. **(E-F)** XTT assay cell viability measurements, plotted as absorbance (450 nm), for Myr-Myc or Myr-oPH-Myc transduced MDA-MB-231 cells either via adenovirus (E**;** n=6 independent experiments) or 20 μg/mL LNPs (F; n=10 independent experiments**)**; two-tailed t-test (E) and Mann-Whitney test (F). **(G)** Representative phase contrast images of adenovirally-transduced, RFP-positive MDA-MB-231 tumorspheres grown in low-attachment conditions, expressing Myr-Myc or Myr-oPH-Myc. Total tumorspheres per well and average tumorsphere size (μm^2) were plotted per treatment group (n = 6 independent experiments); two-tailed t-test. **(H)** Immunoblots of Myr-Myc and Myr-oPH-Myc LNP-treated tumors for PARP and cleaved PARP. Densitometry values are presented as cleaved PARP expression relative to Myr-Myc control (n=9 mice per group pooled from 2 independent experiments; 4-5 per experiment); Mann-Whitney test. **(I)** qPCR of lymph nodes for human Long Interspersed Nuclear Element (hLINE) DNA from LNP-delivered Myr-Myc or Myr-oPH-Myc treated mice. hLINE DNA is plotted as μg hLINE DNA per mg of tissue section (n=9 mice per group pooled from 2 independent experiments; 4-5 mice per experiment); Mann-Whitney test. **(J)** Experimental mouse model schematic detailing LNP treatment of developing orthotopic MDA-MB-231 tumors. $1x10^6$ MDA-MB-231 cells were injected into the mammary fat pad of female NOD SCID mice on Day 0. Seven days following cell implantation, Myr-Myc or Myr-oPH-Myc LNPs were administered at the site of injection once a week for 6 weeks. Endpoint was specified as the point at which 6 total injections per construct had been administered to each mouse, followed by subsequent tissue harvest of the primary tumor, lungs, liver, and lymph nodes; schematic created with Biorender.com (agreement number WU289RLHPI). **(K-L)** qPCR of Myr-Myc **(K)** or Myr-oPH-Myc **(L)** pDNA constructs, plotted as pg pDNA per mg of digested tissue section from LNP-delivered Myr-Myc or Myr-oPH-Myc treated tumors (n=4-5 mice per group); two-tailed t-test **(K)** and Mann-Whitney test **(L)**. **(M)** RT-

qPCR for Myr-oPH-Myc mRNA expression, normalized to GAPDH, for Myr-Myc or Myr-oPH-Myc expressing tumors (n=4-5 mice per group); Mann-Whitney test. **(N)** Caliper measurements of tumor volumes throughout a 6-week course of intratumoral injection of either Myr-Myc or Myr-oPH-Myc LNPs (n=4-5 mice per group); two-way ANOVA. **(O)** qPCR of lymph nodes, lungs, and liver for hLINE DNA from Myr-Myc or Myr-oPH-Myc treated mice. hLINE DNA is plotted as pg hLINE DNA per mg tissue section; n=4-5 mice per group; Mann-Whitney test. **(P)** Immunoblots of Myr-Myc and Myr-oPH-Myc LNP-treated tumors for Pro-MMP9 and MMP9. Densitometry values are presented as Pro-MMP9 and MMP9 expression relative to the Myr-Myc control (n=4-5 mice per group); Mann-Whitney test. **(Q)** cBioPortal Illumina HT-12 v3 microarray for MMP9 expression (TPM $\log_2$) of invasive breast carcinoma patient samples from the 2024 TCGA and Genomic Data Commons dataset, separated by AAJC pathologic T stage (n=40-632 patient samples per T stage); Kruskal-Wallis test with Dunn's multiple comparison test. **(R)** Kaplan–Meier overall survival curves for T1, T2, and T3 breast cancer patients from the cBioPortal METABRIC cohorts (Nature 2012; Nature Communications 2016), stratified by low *versus* high MMP9 mRNA expression, measured using the Illumina HT-12 v3 microarray.; LogRank Test. All data are plotted as mean ± SD **(G, N)** or mean ± SEM (**A-C, E, F, H, I, K-P**); *p<0.05, ***p<0.001, ****p<0.0001.

A

Obscurin PH-Domain Sequence Homology Search

Align.	DB:ID	Protein Source	Corresponding Gene Name	Score (Bits)	Identities %	Positives %	E()-Value
1	SP:OBSCN_HUMAN	Obscurin	OBSCN	262.7	100	100	1.70E-82
2	SP:KALRN_HUMAN	Kalirin	KALRN	65.9	35.4	49.6	1.20E-13
3	SP:PLCG1_HUMAN	Phospholipase C gamma 1	PLCG1	NA	<1	NA	NA

B

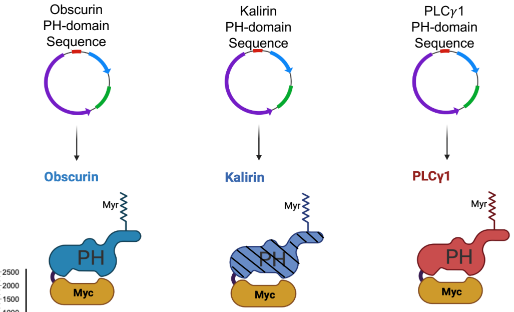

C

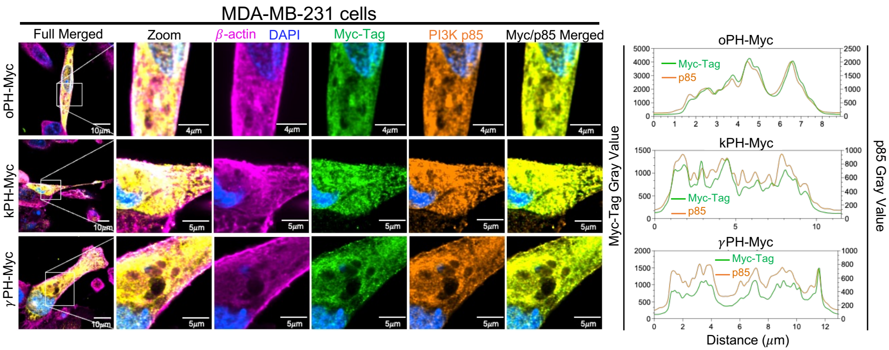

D

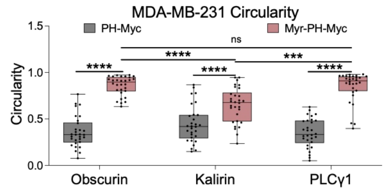

E

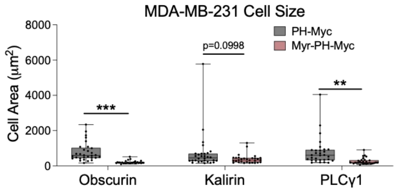

Supplementary Fig. 7

Figure S7: Sequestration of PI3K-p85 by myristoylated obscurin, kalirin, and PLCγ1 PH-domain modulates breast cancer cell morphology. (A) NCBI Blast (https://blast.ncbi.nlm.nih.gov/Blast.cgi?PAGE=Proteins) analysis showing sequence homology between the obscurin PH-domain and the Kalirin or PLCγ1 PH-domains. **(B)** Schematic of plasmid DNA vectors encoding the myristoylated obscurin, kalirin, and PLCγ1 pleckstrin homology (PH)-domains, with C-terminal Myc-Tag; created with Biorender.com (LJ289RL92O). **(C)** Representative confocal images of MDA-MB-231 cells transiently transfected with non-myristoylated oPH-Myc, kPH-Myc, or γPH-Myc constructs; β-actin (magenta), DAPI (blue), Myc-tag (green), PI3K-p85 (orange), and merged Myc-tag/PI3K-p85 (yellow). Cells of each treatment group vary significantly in size; scale bars differ to visualize the entire cell. Corresponding line composite graphs display Myc-tag and PI3K-p85 gray values (pixel intensity). **(D-E)** Cellular circularity **(D)** and cell area (μm^2; **E)** of MDA-MB-231 cells transiently transfected with the obscurin, kalirin, or PLCγ1 PH-Myc or Myr-PH-Myc constructs (n=30 cells pooled from 3 independent experiments); two-way ANOVA with Tukey's **(D)** and Sidak's **(E)** multiple comparison test. All data are plotted as mean ± SD; **p<0.01, ***p<0.001, ****p<0.0001.

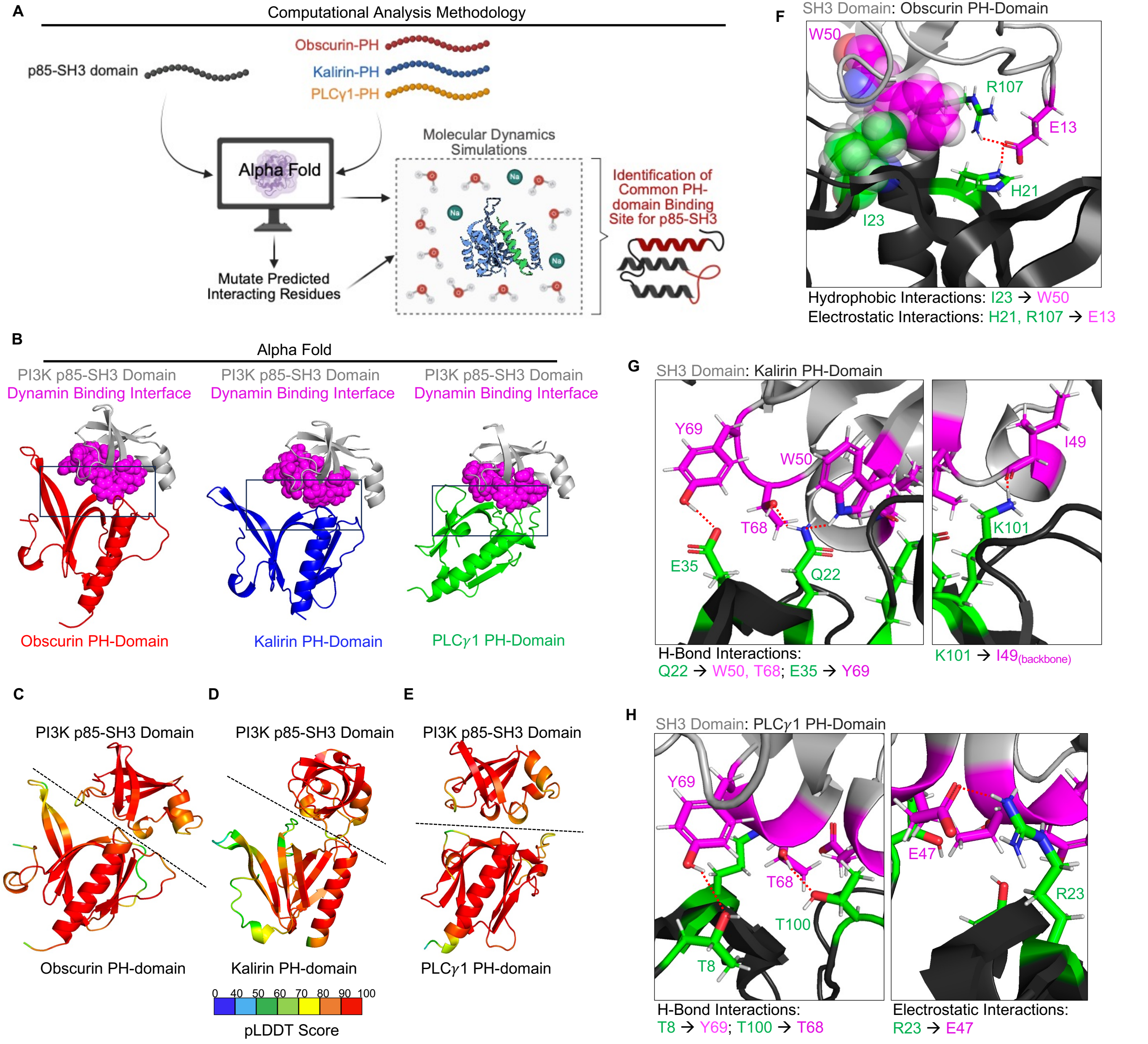

Supplementary Fig. 8

Figure S8: Alpha-fold structural modeling of the obscurin, kalirin, and PLCγ1 PH-domain/PI3K p85-SH3 complex predicts PH-domain binding to the SH3 dynamin binding interface. **(A)** Computational analysis schematic outlining the methodology used to predict the PH-domain binding site for p85-SH3. The obscurin, kalirin, or PLCγ1 PH-domain amino acid sequences were input into Alpha Fold along with the amino acid sequence of the PI3K-p85 SH3 domain. Select PH-domain residues at the PH/SH3 binding interface of each of the obscurin, kalirin, and PLCγ1 PH-domain/PI3K p85-SH3 complexes were "mutated" to ablate the interaction and subsequent molecular dynamics simulations were run to predict the critical PH-domain residues required for stable complex formation with p85-SH3; schematic created with Biorender.com (agreement number YC289RKFZU). **(B)** Equilibrated alpha-fold models of the obscurin (red), kalirin (blue), and PLCγ1(green) PH-domains in complex with the dynamin binding interface (magenta) of the PI3K p85-SH3 domain; the remaining structure of the p85-SH3 domain appears in gray. **(C-E)** Alpha-Fold Predicted Local Distance Difference Test (pLDDT) confidence scoring of equilibrated PH-domain/p85-SH3 alpha-fold models for obscurin-PH **(C)**, kalirin-PH **(D)** and PLCγ1-PH **(E)**; the color scheme key indicates pLDDT scores > 80 (orange and red) versus scores < 80 (blue, green, and yellow). **(F-H)** Zoomed images of the Alpha-Fold predicted PH-domain:SH3-domain binding interface for obscurin **(F)**, kalirin **(G)**, and PLCγ1 **(H)**; PH-domains (black) and critical residues (green); p85 SH3-domain (gray) and key residues (magenta); types of residue bonds are indicated underneath each panel as hydrophobic, electrostatic, or H-bonding. The shortest bond distance between residues is indicated with a dotted red line.

Molecular Dynamics Simulations

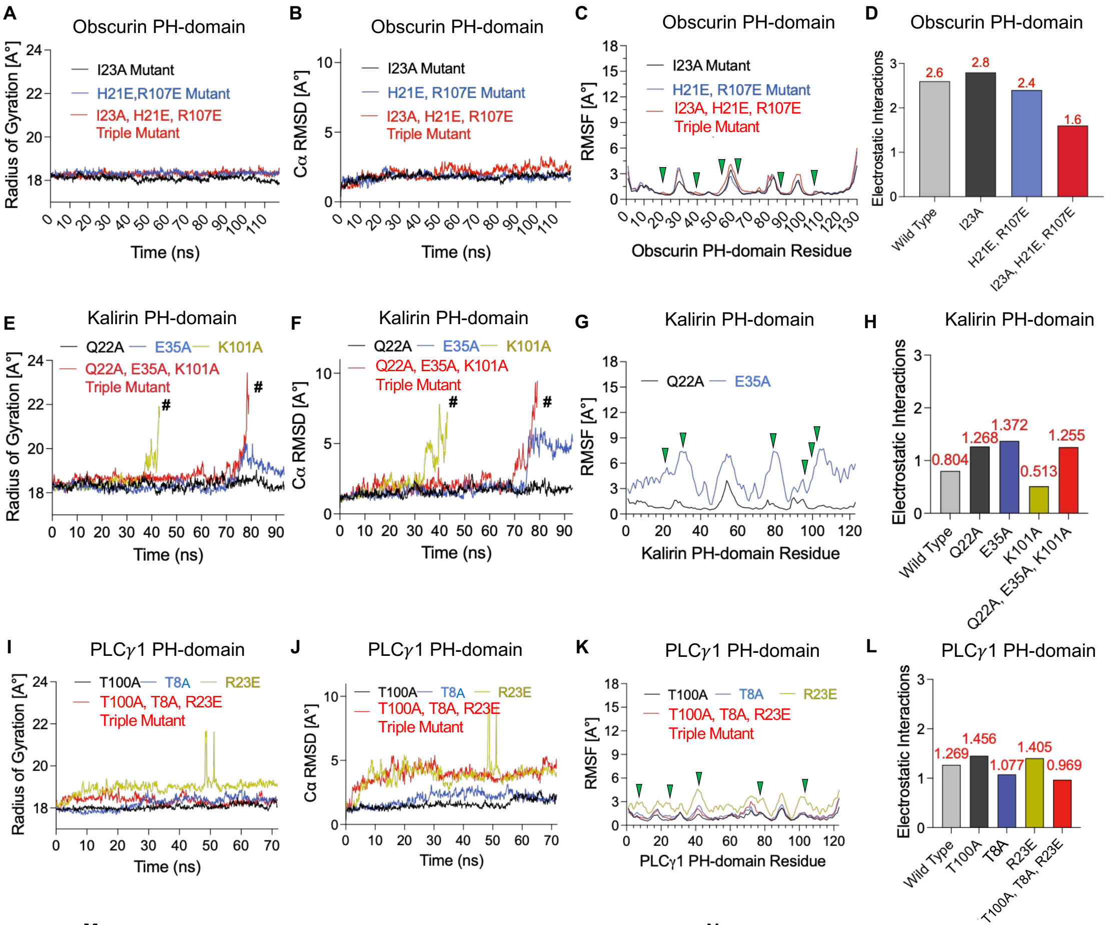

M **Summary of Binding Residues of PH-domain/p85-SH3 Complexes**

Gene/Protein	pLDDT	PH-domain Interacting Residues	p85-SH3 Domain Interacting Residues	Interaction Type
OBSCN/Obscurin	89.8	*β-Strand 1 (I23, H21), β-Strand 7/C-terminal α-helix Linker (R107)*	W50 and E13	Hydrophobic, Electrostatic
KALRN/Kalirin	85.2	*β-Strand 2 (E35), β-Strand 7/C-terminal α-helix Linker (K101)*	Y69 and I49	H-bond
PLCG1/PLCγ1	90.7	*β-Strand 1 (T8), β-Strand 2 (R23)*	Y69 and E47	H-bond, Electrostatic

N

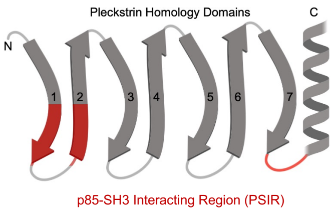

Supplementary Fig. 9

Figure S9: Simulated mutagenesis and molecular dynamics analysis of the obscurin, kalirin, and PLCγ1 PH-domains predict the *p*85-*S*H3 *I*nteracting *R*egion (PSIR). (A-D) Simulated molecular dynamics of the obscurin PH-domain I23A, H21E/R107E, and I23A/H21E/R107E mutants. Radius of gyration (A°; **A**), Cα RMSD (root mean square deviation; **B**), RMSF (root mean square fluctuation) with indicated PH:SH3 interacting sites (green arrowheads; **C**), and average electrostatic interactions (red values above bars) between the obscurin PH-domain mutants and p85-SH3 domain **(D)** are plotted. **(E-H)** Simulated molecular dynamics of the kalirin PH-domain Q22A, E35A, K101A, and Q22A/E35A/K101A mutants. Radius of gyration (A°; **E**), Cα RMSD (**F**), RMSF with indicated PH:SH3 interacting sites (green arrowheads; **G**), and average electrostatic interactions (red values above bars) between the kalirin PH-domain mutants and p85-SH3 domain **(H)** are plotted. **(I-L)** Simulated molecular dynamics of the PLCγ1 PH-domain T100A, T8A, R23E, and T100A/T8A/R23E mutants. Radius of gyration (A°; **I**), Cα RMSD **(J)**, RMSF with indicated PH:SH3 interacting sites (green arrowheads; **K**), and average electrostatic interactions (red values above bars) between the PLCγ1 PH-domain mutants and P85-SH3 domain **(L)** are plotted. **(M)** Summary table of the obscurin, kalirin, and PLCγ1 PH-domain binding residues predicted to be critical for the PH-domain/p85-SH3 complex formation, as determined by simulated mutagenesis and molecular dynamics analysis. The PH-domain regions harboring the p85-SH3 binding residues are highlighted in red. The predicted local distance difference test (pLDDT) score, PH-domain interacting residues, p85-SH3 domain interacting residues, and interaction types are indicated. **(N)** Schematic of the PH-domain region including the second half of β-strand 1, first half of β-strand 2, and the β-strand 7/C-terminal α-helix linker (β-7 linker) that comprises the predicted p85-SH3 Interacting Region (PSIR; red); schematic created with Biorender.com (agreement number BI289RK0DW).

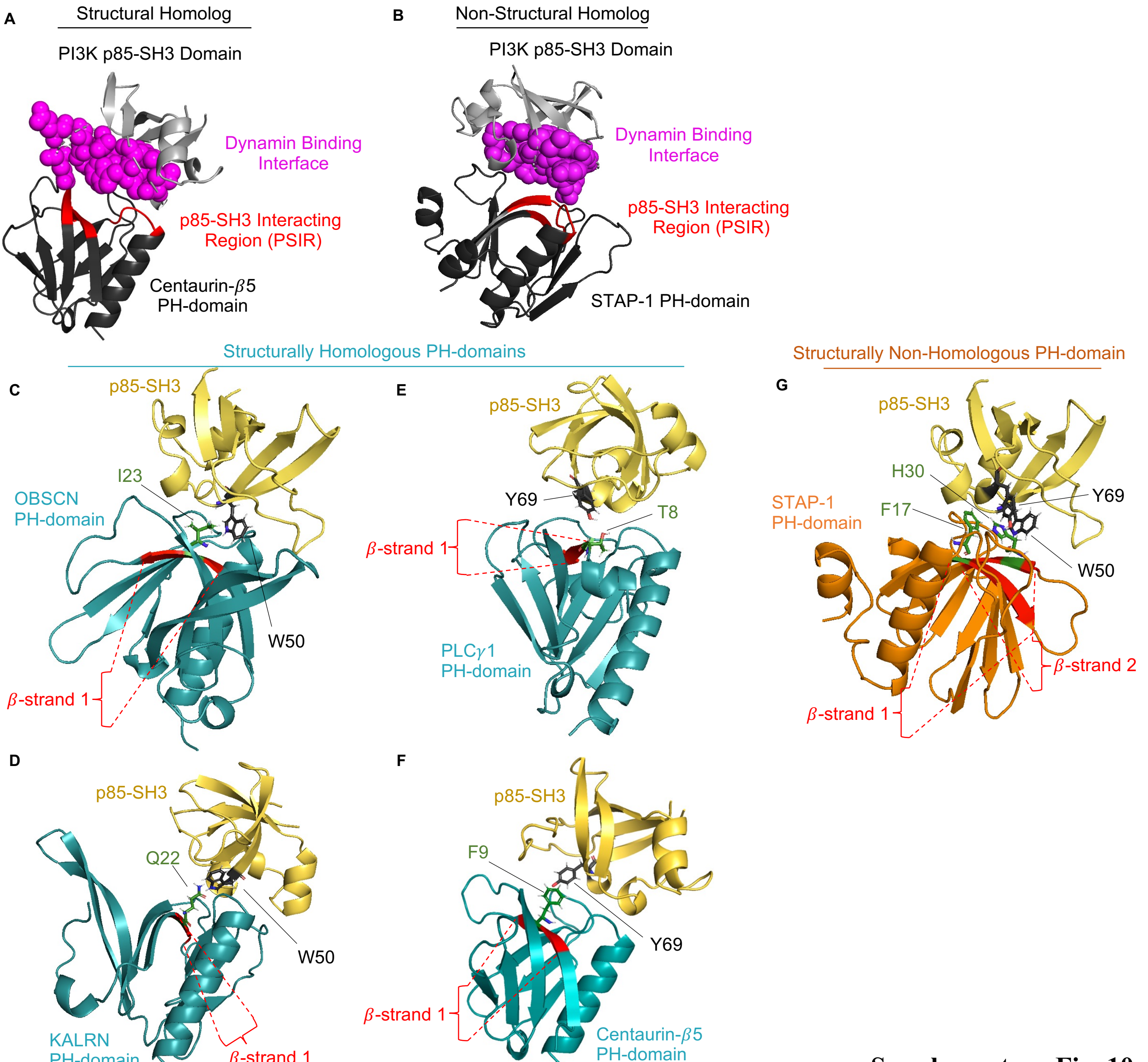

Supplementary Fig. 10

Figure S10: Alpha-fold modeling of structurally and non-structurally homologous PH-domains indicates a PSIR-conserved hydrophobic residue that anchors p85-sequestering PH-domains to p85-SH3. (A-B) Equilibrated alpha-fold models of the structurally homologous Centaurin-β5 **(A)** and non-structurally homologous STAP-1 **(B)** PH-domains (black) complexed with PI3K p85-SH3 (gray); the p85-SH3 dynamin binding interface (magenta spheres) and the p85-SH3 interacting region (PSIR, red) is indicated for each complex. **(C-G)** Equilibrated alpha-fold models of the obscurin **(C),** kalirin **(D)**, PLCγ1 **(E)**, Centaurin-β5 **(F)**, and STAP-1 **(G)** PH-domains (bottom subunit; cyan in C-F and orange in G) in complex with the PI3K p85-SH3 domain (top subunit, yellow). PSIR β-strands are denoted in red and PSIR anchoring hydrophobic residues are shown in green. All p85-SH3 recipient hydrophobic residues are depicted in black.

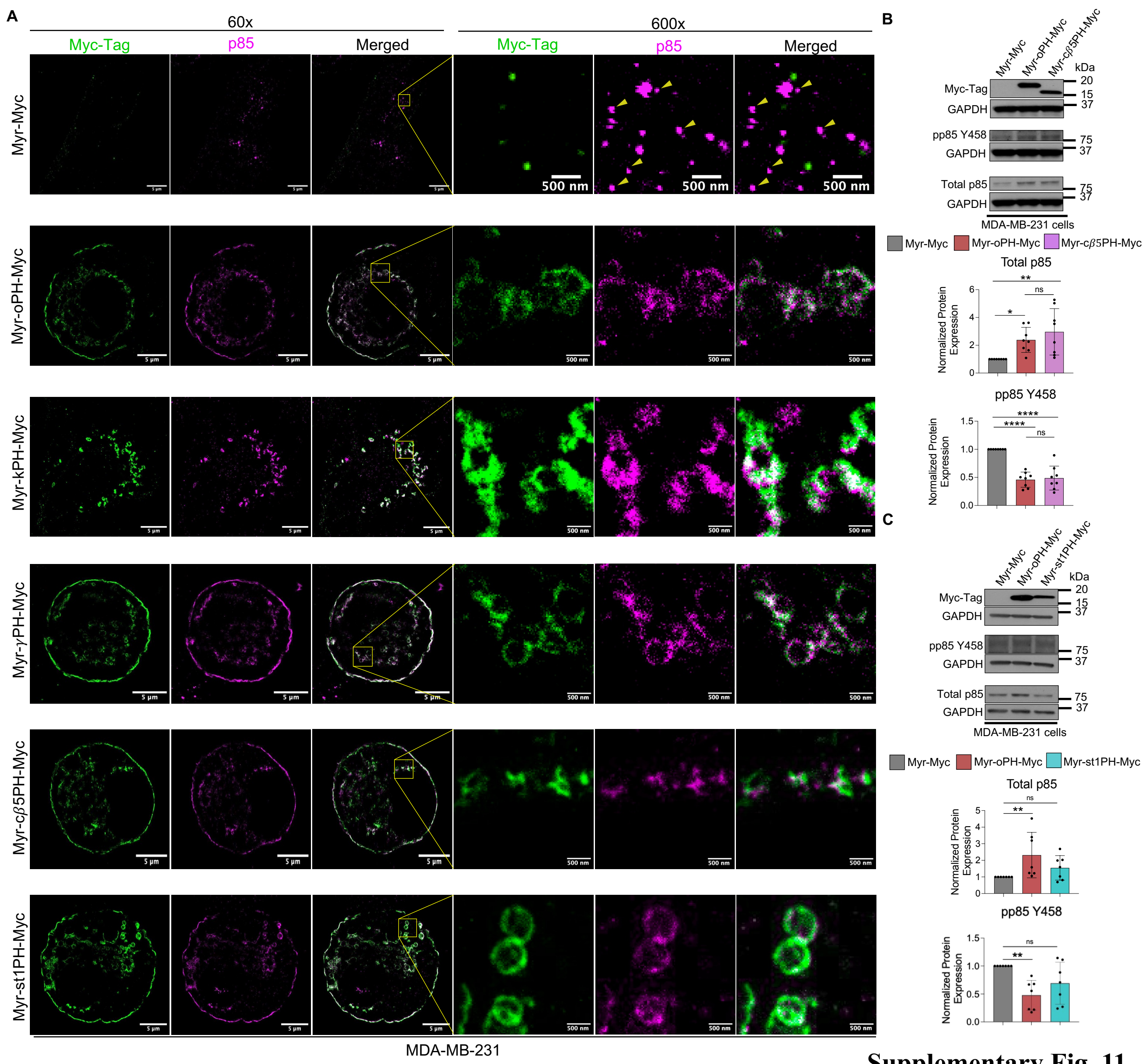

Supplementary Fig. 11

Figure S11: Structurally homologous PH-domains sequester PI3K-p85 to vesicle membranes and block PI3K-p85 activation. (A) Representative images of STED super resolution microscopy of MDA-MB-231 cells transiently transfected with either Myr-Myc, Myr-oPH-Myc, Myr-kPH-Myc, Myr-γPH-Myc, Myr-cβ5PH-Myc, or Myr-st1PH-Myc pDNAs. Single MDA-MB-231 cells (same cells imaged in Fig. 9) immunostained for Myc-tag (green) and PI3K-p85 (magenta) are shown at 60x, while zoom-in areas within the cell body marked with yellow boxes are shown at 600x with areas of colocalization between Myc-tag and PI3K-p85 appearing white; PI3K-p85 cytoplasmic puncta that do not colocalize with Myc-Tag are denoted with yellow arrowheads. **(B)** Western blots of MDA-MB-231 cells transiently transfected with Myr-Myc, Myr-oPH-Myc, or Myr-cβ5PH-Myc constructs, 2 h post-serum stimulus, probed for p85-Tyr458 phosphorylation and total p85 levels, followed by densitometric evaluation (n=8 independent experiments); one-way ANOVA with Tukey's multiple comparison test. **(C)** Western blots of MDA-MB-231 cells transiently transfected with Myr-Myc, Myr-oPH-Myc, or Myr-st1PH-Myc constructs, 2 h post-serum stimulus, probed for p85-Tyr458 phosphorylation and total p85 levels, followed by densitometric evaluation (n=7 independent experiments); one-way ANOVA with Tukey's multiple comparison test. All data are plotted as mean ± SD; *$p<0.05$; **$p<0.01$; ****$p<0.0001$.
