# Supplementary material for: Delivery of Pleckstrin‐Homology Domains Suppresses PI3K/Akt Signaling and Breast Cancer Metastasis
Source: Adv Sci (Weinh). 2026 Mar 30;13(30):e18339. doi: 10.1002/advs.202518339 (PMC13248768; doi:10.1002/advs.202518339)
Supplement: Supplementary file 2 — Supporting File 2: advs74936‐sup‐0002‐TableS1‐S4.zip. [file ADVS-13-e18339-s001.zip › SupplementaryTableS1.pdf]

Table S1: Summary of Myr-Myc and Myr-oPH-Myc Mouse Lung and Liver Pathology Report

LNP Treatment	Secondary Organ Site	Stain Type	Stain	H&E Morphology	IHC	Location	Tumor Grade	Severity Score
Myr-Myc	Lung	H&E	H&E	Multiple nodules, alveolar, tumor, poorly differentiated, anaplastic, abundant cytoplasm, mitoses	NA	Lung, alveoli, peribronchial, peribronchiolar	High	3
		IHC	Human Mitochondria	NA	Positive	Tumor, cytoplasm involving 20-30% of tissue	3+	
Myr-oPH-Myc	Lung	H&E	H&E	Extrinsic, epithelioid cells, large, anaplastic, amphophilic cytoplasm, mitoses, inflammation, scattered microclusters	NA	Lung, alveoli involving 5% of tissue	High	1
		IHC	Human Mitochondria	NA	Positive	Lung, alveoli involving 5% of tissue	3+	
Myr-Myc	Liver	H&E	H&E	Extrinsic, epithelioid cells with large signet ring cell features, EMH scattered, myeloid, erythroid, megakaryocytes	NA	Liver	High	2
		IHC	Human Mitochondria	NA	Positive	Sinusoids, blood, Liver; tumor, strong cytoplasmic involving 10-20% of tissue section	3+	
Myr-oPH-Myc	Liver	H&E	H&E	Extrinsic, epithelioid cells, large, anaplastic, amphophilic cytoplasm	NA	Liver extrinsic detached microclusters	High	1
		IHC	Human Mitochondria	NA	Positive	Tumor, cytoplasmic involving 5%of tissue	3+	
