# Supplementary material for: Delivery of Pleckstrin‐Homology Domains Suppresses PI3K/Akt Signaling and Breast Cancer Metastasis
Source: Adv Sci (Weinh). 2026 Mar 30;13(30):e18339. doi: 10.1002/advs.202518339 (PMC13248768; doi:10.1002/advs.202518339)
Supplement: Supplementary file 2 — Supporting File 2: advs74936‐sup‐0002‐TableS1‐S4.zip. [file ADVS-13-e18339-s001.zip › SupplementaryTableS2.pdf]

Table S2: A subgroup of nine structurally homologous PH-domains contains the PIMS motif

<table>
<tr><th>PH-domain</th><th>PSIR</th><th>PIMS Motif</th><th>p85 Sequestration</th><th>p85 Inhibition</th><th>Akt Suppression</th><th>Metastasis Inhibition</th></tr>
<tr><td>Obscurin</td><td rowspan="4">✓</td><td rowspan="4">✓</td><td rowspan="4">✓</td><td rowspan="4">✓</td><td rowspan="4">✓</td><td rowspan="4">✓</td></tr>
<tr><td>Kalirin</td></tr>
<tr><td>PLCγ1</td></tr>
<tr><td>Centaurin-β5</td></tr>
<tr><td>NF1</td><td rowspan="5">✓</td><td rowspan="5">✓</td><td rowspan="5">✓</td><td rowspan="5">✓</td><td rowspan="5">✓</td><td rowspan="5">✓</td></tr>
<tr><td>RASAL1</td></tr>
<tr><td>PLCB1</td></tr>
<tr><td>TBC1D2A</td></tr>
<tr><td>TBC12B</td></tr>
<tr><td>STAP1</td><td>✓</td><td>–</td><td>✓</td><td>–</td><td>–</td><td>–</td></tr>
</table>
