# Supplementary material for: Delivery of Pleckstrin‐Homology Domains Suppresses PI3K/Akt Signaling and Breast Cancer Metastasis
Source: Adv Sci (Weinh). 2026 Mar 30;13(30):e18339. doi: 10.1002/advs.202518339 (PMC13248768; doi:10.1002/advs.202518339)
Supplement: Supplementary file 2 — Supporting File 2: advs74936‐sup‐0002‐TableS1‐S4.zip. [file ADVS-13-e18339-s001.zip › SupplementaryTableS3.pdf]

Table S3: Pleckstrin Homology Domain Construct Primer Sequences for Cloning

Primer	Forward or Reverse Primer	Primer sequence (5'→3')
Myr-Myc-DDK	Forward	CGCGCCATGGGAAGCAGCAAGA GCAAGCCAAAGC
Myr-Myc DDK	Reverse	TCGAGCTTTGGCTTGCTCTTGCTG CTTCCCATGG
Myr-Myc	Forward	TAAGCAGGCGCGCCATGGGAAGC AGCAAGAGCAAGCCAAAG
Myr-Myc	Reverse	TGCTTAGTTTAAACTTACAGATCC TCTTCTGAGATGAGTTTCTGCTC
OBSCN PH-Myc-DDK	Forward	TAAGCAGGCGCGCCATGCTCATG GAGAACTACCCAGGCACC
OBSCN PH-Myc-DDK	Reverse	TGCTTACTCGAGCAGACGCTGCT GGATGCCACA
OBSCN PH-Myc	Forward	TAAGCAGGCGCGCCATGCTCATG GAGAACTACCCAGGCACC
OBSCN PH-Myc	Reverse	TGCTTAGTTTAAACTTACAGATCC TCTTCTGAGATGAGTTTCTGCTC
OBSCN Myr-PH-Myc	Forward 1	GCAGCAAGAGCAAGCCAAAGCT CATGGAGAACTACCCAGGCACC
OBSCN Myr-PH-Myc	Forward 2	TAAGCAGGCGCGCCATGGGAAGC AGCAAGAGCAAGCCAAAG
OBSCN Myr-PH-Myc	Reverse	TGCTTAGTTTAAACTTACAGATCC TCTTCTGAGATGAGTTTCTGCTC
KALRN PH-Myc DDK	Forward	CTAGATGGCGCGCCATGCTGGAA GGCTTTGATGAAAAC
KALRN PH-Myc DDK	Reverse	TAGCCTCTCGAGAATGCGTTCCT GAATCACTTCGCG
KALRN PH-Myc	Forward	CTAGATGGCGCGCCATGCTGGAA GGCTTTGATGAAAAC
KALRN PH-Myc	Reverse	TGCTCAGTTTAAACTTACAGATCC TCTTCTGAGATGAGTTTCTGCTC
KALRN Myr-PH-Myc	Forward 1	GCAGCAAGAGCAAGCCAAAGAT GCTGGAAGGCTTTGATGAAAAC
KALRN Myr-PH-Myc	Forward 2	TAAGCAGGCGCGCCATGGGAAGC AGCAAGAGCAAGCCAAAG
KALRN Myr-PH-Myc	Reverse	TGCTCAGTTTAAACTTACAGATCC TCTTCTGAGATGAGTTTCTGCTC
PLCG1 PH-Myc	Forward	CTAGATGGCGCGCCATGCTGGAA GTGGGCACCGTGATG
PLCG1 PH-Myc	Reverse	TGCTCAGTTTAAACTTACAGATCC TCTTCTGAGATGAGTTTCTGCTC
PLCG1 Myr-PH-Myc	Forward	TAAGCAGGCGCGCCATGGGAAGC AGCAAGAGCAAGCCAAAG

PLCG1 Myr-PH-Myc	Reverse	TGCTCAGTTTAAACTTACAGATCC TCTTCTGAGATGAGTTTCTGCTC
CENTB5 Myr-PH-Myc	Forward	TAAGCAGGCGCGCCATGGGAAGC AGCAAGAGCAAGCCAAAG
CENTB5 Myr-PH-Myc	Reverse	TGCTCAGTTTAAACTTACAGATCC TCTTCTGAGATGAGTTTCTGCTC
STAP1 Myr-PH-Myc	Forward	TAAGCAGGCGCGCCATGGGAAGC AGCAAGAGCAAGCCAAAG
STAP1 Myr-PH-Myc	Reverse	TGCTCAGTTTAAACTTACAGATCC TCTTCTGAGATGAGTTTCTGCTC
