# Supplementary material for: Delivery of Pleckstrin‐Homology Domains Suppresses PI3K/Akt Signaling and Breast Cancer Metastasis
Source: Adv Sci (Weinh). 2026 Mar 30;13(30):e18339. doi: 10.1002/advs.202518339 (PMC13248768; doi:10.1002/advs.202518339)
Supplement: Supplementary file 2 — Supporting File 2: advs74936‐sup‐0002‐TableS1‐S4.zip. [file ADVS-13-e18339-s001.zip › SupplementaryTableS4.pdf]

Table S4: Primers for qPCR and RT-qPCR of Mouse Tissues

Primer	Forward or Reverse Primer	Primer sequence (5'→3')
Myr-Myc	Forward	CAAGCCAAAGCTCGAGCAGA
Myr-Myc	Reverse	CTTGCCCCTTGCTCCATACC
Myr-oPH-Myc	Forward	AGCAAGCCAAAGCTCATGGA
Myr-oPH-Myc	Reverse	AGATTACCAGGTGGTTGCGG
hLINE	Forward	TCACTCAAAGCCGCTCAACTA
hLINE	Reverse	TCTGCCTTCATTTCGTTATGTACC
GAPDH	Forward	TCGGAGTCAACGGATTTG
GAPDH	Reverse	CAACAATATCCACTTTACCAGAG
