# Supplementary material for: Delivery of Pleckstrin‐Homology Domains Suppresses PI3K/Akt Signaling and Breast Cancer Metastasis
Source: Adv Sci (Weinh). 2026 Mar 30;13(30):e18339. doi: 10.1002/advs.202518339 (PMC13248768; doi:10.1002/advs.202518339)
Supplement: Supplementary file 3 — Supporting File 3: advs74936‐sup‐0003‐VideoS1‐S4.zip. [file ADVS-13-e18339-s003.zip › SupplementaryVideoLegends.pdf]

Supplementary Video Legends

Supplementary Video S1

Time-lapse microscopy of MDA-MB-231 Myr-Myc or Myr-oPH-Myc expressing single cells migrating (bottom to top) through microchannels. Time is indicated in hh:mm:ss.

Supplementary Video S2

Time-lapse microscopy of MDA-MB-231 Myr-Myc or Myr-oPH-Myc expressing spheroids disseminating into 3D collagen I. Time is indicated in hh:mm:ss.

Supplementary Video S3

Time-lapse microscopy of single MDA-MB-231 cells (yellow arrowheads) from Myr-Myc or Myr-oPH-Myc expressing spheroids, dissociating from the spheroid edge into 3D collagen I. Time is indicated in hh:mm:ss.

Supplementary Video S4

Time-lapse microscopy of MDA-MB-231 3D breast cancer spheroids treated with lipid nanoparticles delivering the Myr-Myc, Myr-oPH-Myc, Myr-kPH-Myc, or Myr-γ(g)PH-Myc plasmid DNA constructs and disseminating into 3D collagen I. Time is indicated in hh:mm:ss.
